# Supplementary material for: Testing for Sufficient Follow‐Up in Survival Data With a Cure Fraction
Source: Biom J. 2026 Mar 4;68(2):e70121. doi: 10.1002/bimj.70121 (PMC12961184; doi:10.1002/bimj.70121)
Supplement: Supplementary file 1 — Supporting File 1: bimj70121‐sup‐0001‐DataCode.zip. [file BIMJ-68-e70121-s001.zip › code and data/simulation/simulation_results.pdf]

Testing for sufficient follow-up in survival data with a cure fraction

Simulation results

Yuen, T. P. and Musta E.

2025-11-25

Setting 1

This section contains code for generating the figures in Section 4.2 of the main manuscript and in Section S1.2.1 of the Supplementary Material using the intermediate results.

```
lambda.all <- c(1)
jumpsize.tau.c.all <- c(0, 0.02, 0.05, 0.2)
p.all <- c(0.2, 0.4, 0.8) # cure fraction
size.all <- c(200, 500, 1000)
res.file.path <- "./intermediate_results/setting1"

melt.id.vars <- c("p.tau_c", "tau.c", "size")
melt.measure.vars <- c("sg.H0.is.reject",
                       "gren.H0.is.reject",
                       "alpha.H0.is.reject", "alpha.tilde.H0.is.reject",
                       "q.n.H0.is.reject")
legend.names <- expression(hat(f)[n]^SG, hat(f)[n]^G,
                           hat(alpha)[n], tilde(alpha)[n], Q[n])
jumpsize.labels <- paste('Delta*G(tau[G]) == ', jumpsize.tau.c.all, sep = "")
size.labels <- paste('Size == ', size.all, sep = "")

plots.list <- NULL
all.rej.df <- NULL
for (p in p.all) {
  for (lambda in lambda.all) {
    size.jumpsize.rej.df <- NULL
    for (jumpsize.tau.c in jumpsize.tau.c.all) {
      for (size in size.all) {
        rej.df <- NULL
        res.file.subdir <- sprintf("exp_%s_unif_%s_p_%s_n_%d",
                                   as.character(lambda),
                                   as.character(jumpsize.tau.c),
                                   as.character(p), size)
        rds.files <- list.files(file.path(res.file.path, res.file.subdir),
                                pattern = "results_grid_no_clip.rds$",
                                full.names = TRUE)
        if (length(rds.files) == 1L) {
```

```
      res <- readRDS(rds.files)
      has.no.tau.c <- all(
        !(res$rej.prop.df$tau.c %in%
            rej.df[rej.df$jumpsize.tau.c == jumpsize.tau.c, ]$tau.c))
      if (has.no.tau.c) {
        rej.df.tmp <- res$rej.prop.df
        rej.df.tmp$p.tau_c <- pexp(rej.df.tmp$tau.c, rej.df.tmp$lambda)
        rej.df <- rbind(rej.df, rej.df.tmp)
      }

      has.no.tau.c.size <- all(
        !(res$rej.prop.df$tau.c %in%
            size.jumpsize.rej.df[
              size.jumpsize.rej.df$jumpsize.tau.c == jumpsize.tau.c &
                size.jumpsize.rej.df$size == size, ]$tau.c))
      if (has.no.tau.c.size) {
        rej.df.tmp <- res$rej.prop.df
        rej.df.tmp$p.tau_c <- pexp(rej.df.tmp$tau.c, rej.df.tmp$lambda)
        size.jumpsize.rej.df <- rbind(size.jumpsize.rej.df, rej.df.tmp)
        all.rej.df <- rbind(all.rej.df, rej.df.tmp)
      }
    }
  }
}
plot.df <- as.data.table(size.jumpsize.rej.df)
plot.df <- melt(plot.df, id.vars = c(melt.id.vars, "jumpsize.tau.c"),
                measure.vars = melt.measure.vars,
                variable.factor = TRUE)
plot.df$jumpsize.tau.c <- factor(plot.df$jumpsize.tau.c,
                                 levels = jumpsize.tau.c.all,
                                 labels = jumpsize.labels)
plot.df$size <- factor(plot.df$size, levels = size.all, labels = size.labels)
plot.x.labels <- paste("q", seq(length(unique(plot.df$p.tau_c))), sep = "")
g1 <- ggplot(plot.df, aes(x = tau.c, y = value, group = variable,
                          color = variable, linetype = variable)) +
  geom_line(linewidth = 1) +
  geom_hline(yintercept = unique(size.jumpsize.rej.df$alpha),
             colour = 'red', linetype = '3313', linewidth = 1) +
  geom_vline(xintercept = unique(
    plot.df[plot.df$p.tau_c == 1 - unique(size.jumpsize.rej.df$eps), ][["tau.c"]]),
    colour = 'black', linetype = '3313', alpha = 0.35, linewidth = 1) +
  scale_x_continuous(breaks = sort(unique(plot.df$tau.c)),
                     labels = plot.x.labels) +
  scale_y_continuous(breaks = seq(0, 1, 0.1), limits = c(0, 1)) +
  theme_bw() +
  theme(legend.position="bottom",
        text = element_text(size = 30),
        axis.text.x = element_text(angle = 90, vjust = 0.5, hjust = 1),
        legend.text.align = 0,
        legend.key.width = unit(0.08, "npc"),
        legend.box.margin = margin(t = -10, r = 25, b = 0, l = 0, unit = "pt")) +
  facet_grid(row = vars(size), cols = vars(jumpsize.tau.c),
             labeller = label_parsed) +
  labs(color  = "Method", linetype = "Method") +
  xlab(~ paste(tau["G"])) + ylab("Rejecting proportion") +
  scale_color_hue(labels = legend.names) +
  scale_linetype_discrete(labels = legend.names)
```

```
    list.tmp <- list(g1)
    names(list.tmp) <- p
    plots.list <- c(plots.list, list.tmp)
  }
}
```

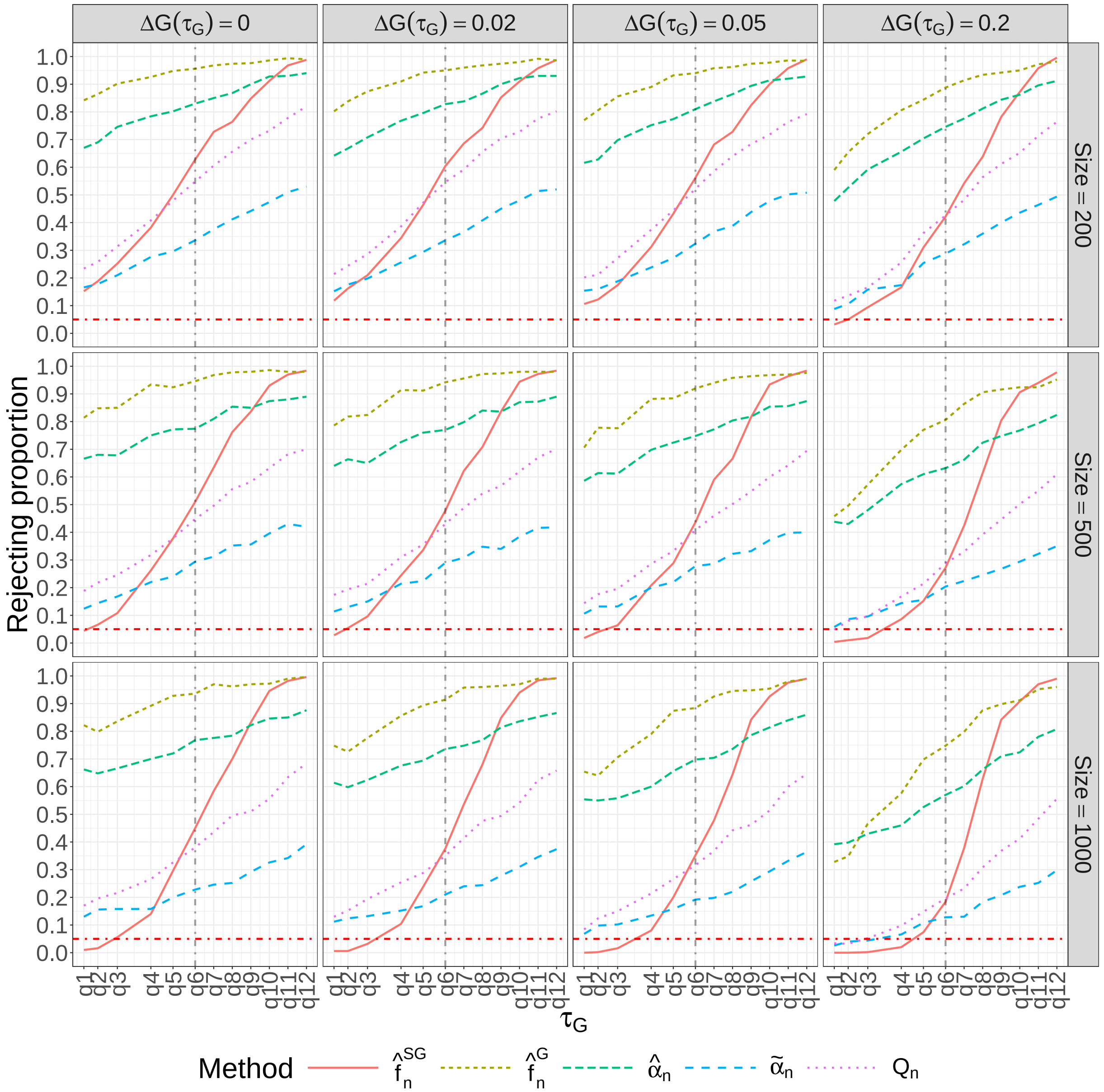

Figure 1: Rejection rate of the null hypothesis of insufficient follow-up for different methods in Setting 1 when p = 0.2 (uncured fraction). (Figure S1 in the Supplementary Material)

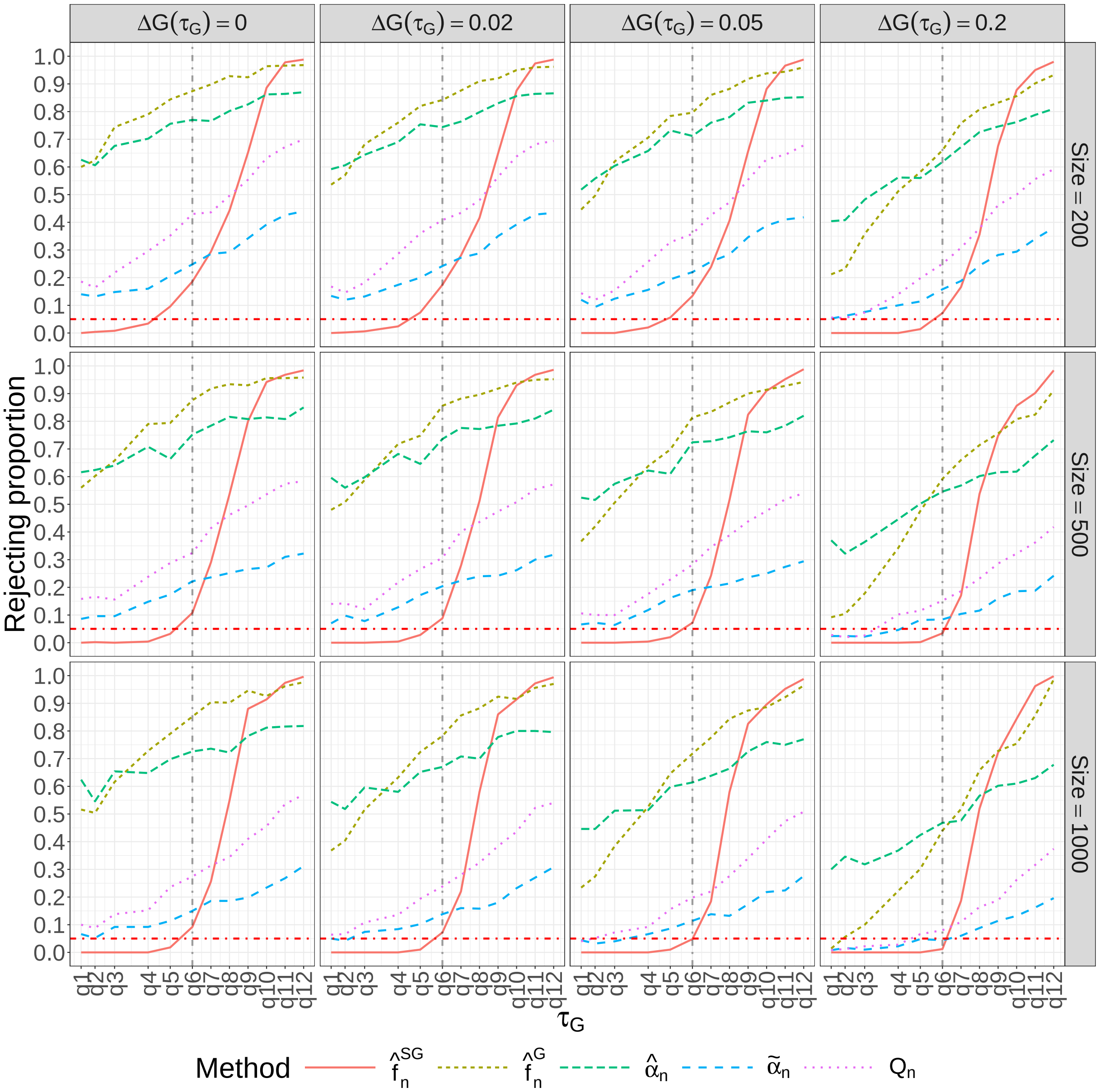

Figure 2: Rejection rate of the null hypothesis of insufficient follow-up for different methods in Setting 1 when p = 0.6 (uncured fraction). (Figure S2 in the Supplementary Material)

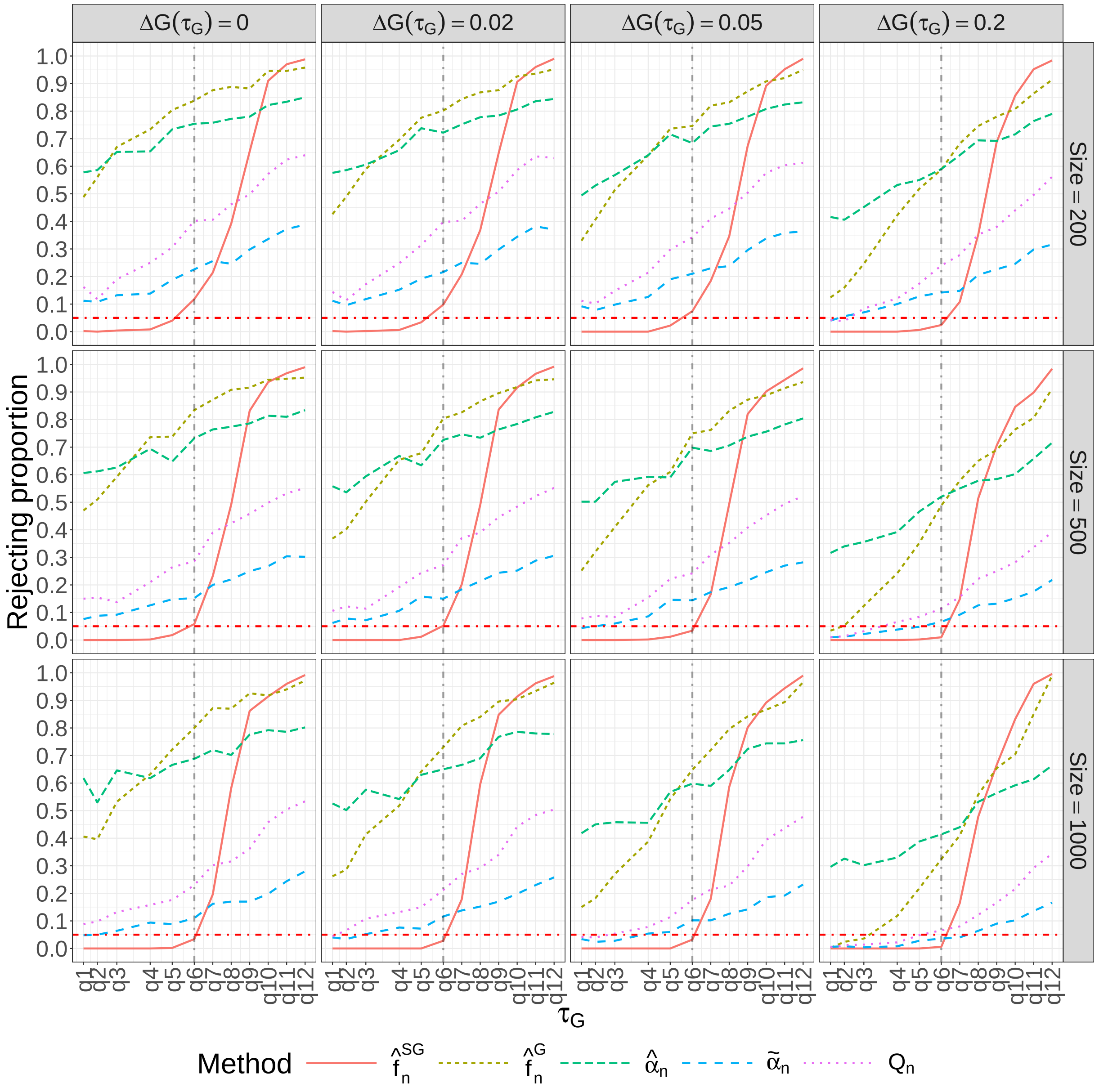

Figure 3: Rejection rate of the null hypothesis of insufficient follow-up for different methods in Setting 1 when p = 0.8 (uncured fraction). (Figure S3 in the Supplementary Material)

```
legend.names.bw <- legend.names[-c(3,4)]
col.sel <- c('lambda', 'tau.c', 'jumpsize.tau.c', 'p',
             'size', 'eps', 'alpha', 'tau', 'H0.insufficient',
             'gren.H0.is.reject', 'sg.H0.is.reject', 'q.n.H0.is.reject', 'p.tau_c')
plot.df <- as.data.table(all.rej.df[all.rej.df$size == 500 &
                                      all.rej.df$jumpsize.tau.c == 0.02, col.sel])
plot.df$t.n.H1.is.reject <- 1 - plot.df$t.n.H1.is.reject
plot.df$p <- 1 - plot.df$p
plot.df <- melt(plot.df, id.vars = c(melt.id.vars, "p", "jumpsize.tau.c"),
                measure.vars = melt.measure.vars[-c(3,4)],
                variable.factor = TRUE)
plot.df$jumpsize.tau.c <- factor(plot.df$jumpsize.tau.c, levels = jumpsize.tau.c.all,
                                 labels = jumpsize.labels)
plot.df$size <- factor(plot.df$size, levels = size.all, labels = size.labels)
```

```r
p.labels <- paste('p == ', rev(1 - p.all), sep = "")
plot.df$p <- factor(plot.df$p, levels = rev(1 - p.all), labels = p.labels)
plot.x.labels <- paste("q", seq(length(unique(plot.df$p.tau_c))), sep = "")
g1.bw <- ggplot(plot.df, aes(x = tau.c, y = value, group = variable,
                             color = variable, linetype = variable)) +
  geom_line(linewidth = 1.05) +
  geom_hline(yintercept = unique(size.jumpsize.rej.df$alpha), colour = 'black', linetype = '3313',
             linewidth = 1.05, alpha = 0.35) +
  geom_vline(xintercept = unique(plot.df[plot.df$p.tau_c == 1 - unique(size.jumpsize.rej.df$eps), ][["tau.c"]]),
             colour = 'black', linetype = '3313', alpha = 0.35, linewidth = 1.05) +
  scale_x_continuous(breaks = sort(unique(plot.df$tau.c)),
                     labels = plot.x.labels) +
  scale_y_continuous(breaks = seq(0, 1, 0.1), limits = c(0, 1)) +
  theme_bw() +
  theme(legend.position="bottom",
        text = element_text(size = 25),
        axis.text.x = element_text(angle = 90, vjust = 0.5, hjust = 1, size = 16),
        legend.text.align = 0,
        legend.key.width = unit(0.08, "npc"),
        legend.box.margin = margin(t = -15, r = 25, b = 0, l = 0, unit = "pt")) +
  facet_grid(cols = vars(p),
             labeller = label_parsed) +
  labs(color  = "Method", linetype = "Method") +
  xlab(~ paste(tau["G"])) + ylab("Rejecting proportion") +
  scale_color_grey(labels = legend.names.bw, start = 0.2, end = 0.2) +
  # scale_linetype_discrete(labels = legend.names) +
  scale_linetype_manual(labels = legend.names.bw,
                        values = c("solid", "dashed", "dotted", "twodash", "dotdash", "longdash"))

g1.bw <- g1.bw + theme(legend.position="blank")
g1.bw
```

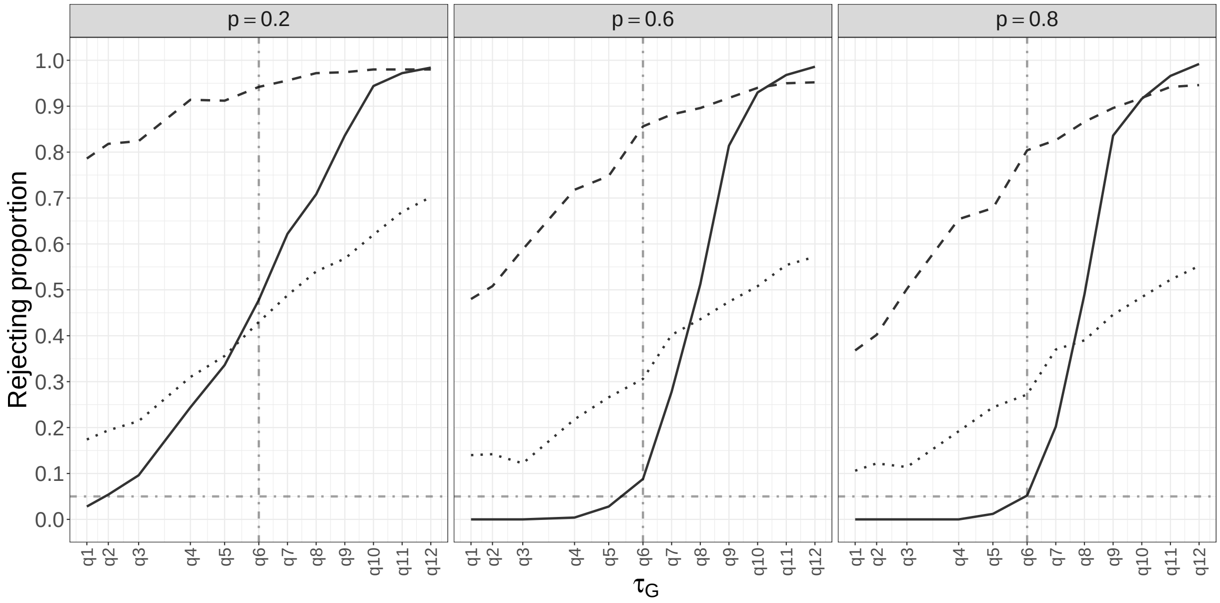

Figure 4: Rejection rate of the null hypothesis of insufficient follow-up for different methods (solid: $\hat{f}_{nh}^{SG}$, dashed: $\hat{f}_n^G$, dotted: Q_n) in Setting 1 with $n = 500$, $\Delta G(\tau_G) = 0.02$ and $p = 0.2$ (left), $p = 0.6$ (center), $p = 0.8$ (right). (Figure 1 in the main manuscript)

```r
legend.names.bw <- legend.names[-c(3,4)]
col.sel <- c('lambda', 'tau.c', 'jumpsize.tau.c', 'p',
             'size', 'eps', 'alpha', 'tau', 'H0.insufficient',
             'gren.H0.is.reject', 'sg.H0.is.reject', 'q.n.H0.is.reject', 'p.tau_c')
plot.df <- as.data.table(all.rej.df[all.rej.df$size == 500 &
                                      all.rej.df$p == 0.4 &
                                      all.rej.df$jumpsize.tau.c != 0.05, col.sel])
plot.df$t.n.H1.is.reject <- 1 - plot.df$t.n.H1.is.reject
plot.df <- melt(plot.df, id.vars = c(melt.id.vars, "p", "jumpsize.tau.c"),
                measure.vars = melt.measure.vars[-c(3,4)],
                variable.factor = TRUE)
plot.df$jumpsize.tau.c <- factor(plot.df$jumpsize.tau.c, levels = jumpsize.tau.c.all,
                                labels = jumpsize.labels)
plot.df$size <- factor(plot.df$size, levels = size.all, labels = size.labels)
p.labels <- paste('p == ', 1 - p.all, sep = "")
plot.df$p <- factor(plot.df$p, levels = p.all, labels = p.labels)
plot.x.labels <- paste("q", seq(length(unique(plot.df$p.tau_c))), sep = "")
plot.x.labels[c(2,5,7,9,11)] <- ""

g1.bw <- ggplot(plot.df, aes(x = tau.c, y = value, group = variable,
                             color = variable, linetype = variable)) +
  geom_line(linewidth = 1.05) +
  geom_hline(yintercept = unique(size.jumpsize.rej.df$alpha), colour = 'black', linetype = '3313',
             linewidth = 1.05, alpha = 0.35) +
  geom_vline(xintercept = unique(plot.df[plot.df$p.tau_c == 1 - unique(size.jumpsize.rej.df$eps), ][["tau.c"]]),
             colour = 'black', linetype = '3313', alpha = 0.35, linewidth = 1.05) +
  scale_x_continuous(breaks = sort(unique(plot.df$tau.c)),
                     labels = plot.x.labels) +
  scale_y_continuous(breaks = seq(0, 1, 0.1), limits = c(0, 1)) +
  theme_bw() +
  theme(legend.position="bottom",
        text = element_text(size = 25),
        axis.text.x = element_text(angle = 90, vjust = 0.5, hjust = 1, size = 16),
        legend.text.align = 0,
        legend.key.width = unit(0.08, "npc"),
        legend.box.margin = margin(t = -15, r = 25, b = 0, l = 0, unit = "pt")) +
  facet_grid(cols = vars(jumpsize.tau.c),
             labeller = label_parsed) +
  labs(color  = "Method", linetype = "Method") +
  xlab(~ paste(tau["G"])) + ylab("Rejecting proportion") +
  scale_color_grey(labels = legend.names.bw, start = 0.2, end = 0.2) +
  # scale_linetype_discrete(labels = legend.names.bw)
  scale_linetype_manual(labels = legend.names.bw,
                        values = c("solid", "dashed", "dotted", "twodash", "dotdash", "longdash"))

g1.bw <- g1.bw + theme(legend.position="blank")
g1.bw
```

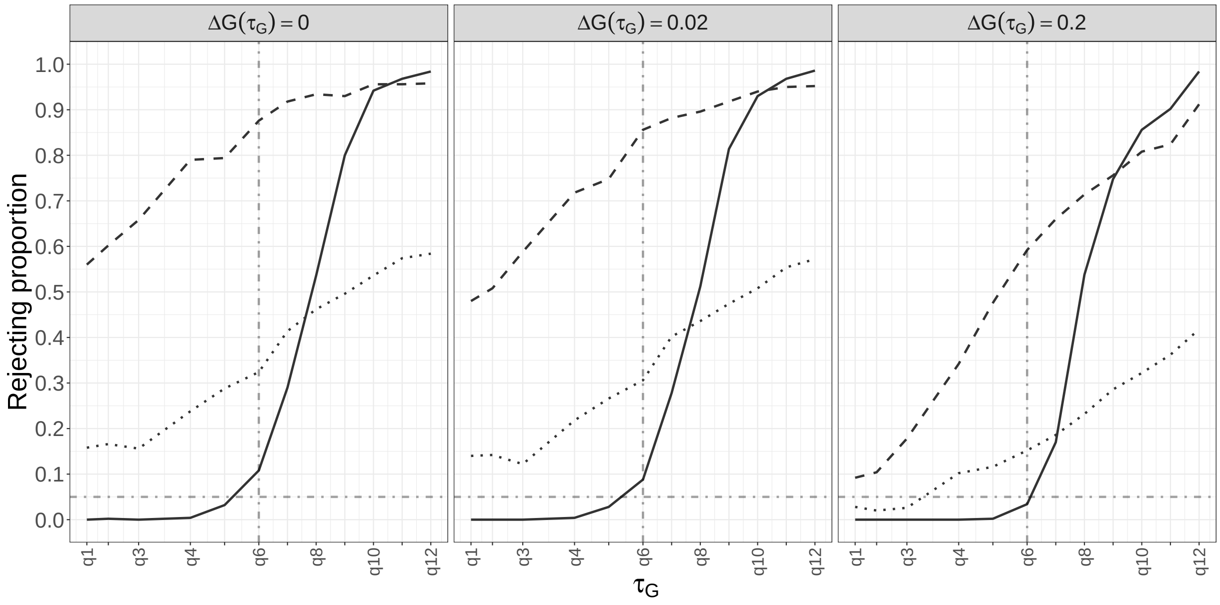

Figure 5: Rejection rate of the null hypothesis of insufficient follow-up for different methods (solid: $\hat{f}^{SG}_{nh}$, dashed: $\hat{f}^{G}_{n}$, dotted: Q_n) in Setting 1 with $n = 500$, $p = 0.6$ and $\Delta G(\tau_G) = 0$ (left), 0.02 (center), and 0.2 (right). (Figure 2 in the main manuscript)

```
legend.names.bw <- legend.names[-c(3,4)]
col.sel <- c('lambda', 'tau.c', 'jumpsize.tau.c', 'p',
             'size', 'eps', 'alpha', 'tau', 'H0.insufficient',
             'gren.H0.is.reject', 'sg.H0.is.reject', 'q.n.H0.is.reject', 'p.tau_c')
plot.df <- as.data.table(all.rej.df[all.rej.df$p == 0.4 &
                                      all.rej.df$jumpsize.tau.c == 0.02, col.sel])
plot.df$t.n.H1.is.reject <- 1 - plot.df$t.n.H1.is.reject
plot.df <- melt(plot.df, id.vars = c(melt.id.vars, "p", "jumpsize.tau.c"),
                measure.vars = melt.measure.vars[-c(3,4)],
                variable.factor = TRUE)
plot.df$jumpsize.tau.c <- factor(plot.df$jumpsize.tau.c, levels = jumpsize.tau.c.all,
                                 labels = jumpsize.labels)
plot.df$size <- factor(plot.df$size, levels = size.all, labels = size.labels)
p.labels <- paste('p == ', 1 - p.all, sep = "")
plot.df$p <- factor(plot.df$p, levels = p.all, labels = p.labels)
plot.x.labels <- paste("q", seq(length(unique(plot.df$p.tau_c))), sep = "")
plot.x.labels[c(2,5,7,9,11)] <- ""

g1.bw <- ggplot(plot.df, aes(x = tau.c, y = value, group = variable,
                          color = variable, linetype = variable)) +
  geom_line(linewidth = 1.05) +
  geom_hline(yintercept = unique(size.jumpsize.rej.df$alpha), colour = 'black', linetype = '3313',
             linewidth = 1.05, alpha = 0.35) +
  geom_vline(xintercept = unique(plot.df[plot.df$p.tau_c == 1 - unique(size.jumpsize.rej.df$eps), ][["tau.c"]]),
             colour = 'black', linetype = '3313', alpha = 0.35, linewidth = 1.05) +
  scale_x_continuous(breaks = sort(unique(plot.df$tau.c)),
                     labels = plot.x.labels) +
  scale_y_continuous(breaks = seq(0, 1, 0.1), limits = c(0, 1)) +
  theme_bw() +
  theme(legend.position="bottom",
        text = element_text(size = 25),
        axis.text.x = element_text(angle = 90, vjust = 0.5, hjust = 1, size = 16),
```

```
        legend.text.align = 0,
        legend.key.width = unit(0.08, "npc"),
        legend.box.margin = margin(t = -15, r = 25, b = 0, l = 0, unit = "pt")) +
  facet_grid(cols = vars(size),
             labeller = label_parsed) +
  labs(color  = "Method", linetype = "Method") +
  xlab(~ paste(tau["G"])) + ylab("Rejecting proportion") +
  scale_color_grey(labels = legend.names.bw, start = 0.2, end = 0.2) +
  # scale_linetype_discrete(labels = legend.names.bw)
  scale_linetype_manual(labels = legend.names.bw,
                        values = c("solid", "dashed", "dotted", "twodash", "dotdash", "longdash"))
g1.bw <- g1.bw + theme(legend.position="blank")
g1.bw
```

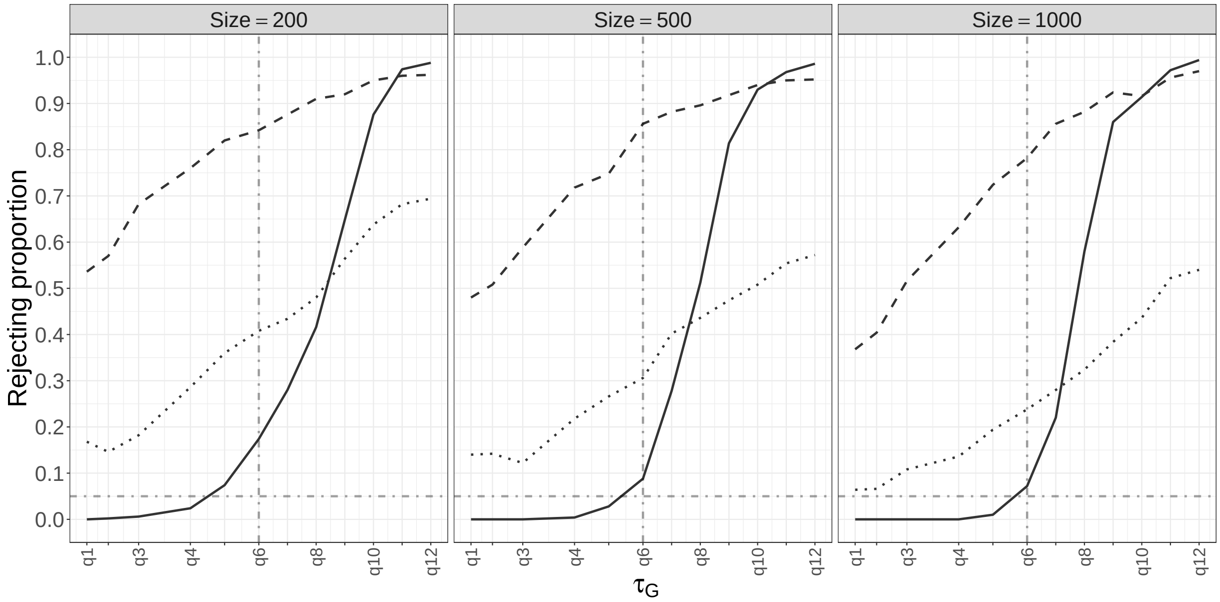

Figure 6: Rejection rate of the null hypothesis of insufficient follow-up for different methods (solid: $\hat{f}_{nh}^{SG}$, dashed: $\hat{f}_{n}^{G}$, dotted: Q_n) in Setting 1 with $p = 0.6$, $\Delta G(\tau_G) = 0.02$ and a sample size of 200 (left), 500 (center), and 1000 (right). (Figure 3 in the main manuscript)

Sensitivity of τ

This subsection contains code for generating the figures in Section S1.2.1 to study the sensitivity of τ.

```
lambda.all <- c(1)
jumpsize.tau.c.all <- c(0, 0.02, 0.05, 0.2)
p.all <- c(0.2, 0.4, 0.8) # cure fraction
size.all <- c(200, 500, 1000)
res.file.path <- "./intermediate_results/setting1"

melt.id.vars <- c("p.tau_c", "tau.c", "size", "tau")
melt.measure.vars <- c(
  "sg.H0.is.reject"
)
legend.names <- expression(hat(f)[n]^G, hat(f)[n]^SG,
                           hat(alpha)[n], tilde(alpha)[n], Q[n])
jumpsize.labels <- paste('Delta*G(tau[G]) == ', jumpsize.tau.c.all, sep = "")
size.labels <- paste('Size == ', size.all, sep = "")

plots.list <- NULL

for (p in p.all) {
  for (lambda in lambda.all) {
    size.jumpsize.rej.df <- NULL
    for (jumpsize.tau.c in jumpsize.tau.c.all) {
      for (size in size.all) {
        rej.df <- NULL
        res.file.subdir <- sprintf("exp_%s_unif_%s_p_%s_n_%d",
                                   as.character(lambda),
                                   as.character(jumpsize.tau.c),
                                   as.character(p), size)
        rds.files.tau1 <- list.files(file.path(res.file.path, res.file.subdir),
                                     pattern = "results_grid_no_clip_1.rds$",
                                     full.names = TRUE)
        rds.files.tau2 <- list.files(file.path(res.file.path, res.file.subdir),
                                     pattern = "results_grid_no_clip_2.rds$",
                                     full.names = TRUE)
        rds.files.tau3 <- list.files(file.path(res.file.path, res.file.subdir),
                                     pattern = "results_grid_no_clip_3.rds$",
                                     full.names = TRUE)
        for (rds.files in c(rds.files.tau1, rds.files.tau2, rds.files.tau3)) {
          if (length(rds.files) == 1L) {
            res <- readRDS(rds.files)
            has.no.tau.c <- all(!(res$rej.prop.df$tau.c %in%
                                    rej.df[rej.df$jumpsize.tau.c == jumpsize.tau.c, ]$tau.c))
            has.no.tau <- all(!(res$rej.prop.df$tau %in%
                                  rej.df[rej.df$jumpsize.tau.c == jumpsize.tau.c, ]$tau))
            if (has.no.tau.c || has.no.tau) {
              rej.df.tmp <- res$rej.prop.df
              rej.df.tmp$p.tau_c <- pexp(rej.df.tmp$tau.c, rej.df.tmp$lambda)
              rej.df <- rbind(rej.df, rej.df.tmp)
            }

            has.no.tau.c.size <- all(
              !(res$rej.prop.df$tau.c %in%
                  size.jumpsize.rej.df[
```

```
                  size.jumpsize.rej.df$jumpsize.tau.c == jumpsize.tau.c &
                    size.jumpsize.rej.df$size == size, ]$tau.c))
          has.no.tau.size <- all(
            !(res$rej.prop.df$tau %in%
                size.jumpsize.rej.df[
                  size.jumpsize.rej.df$jumpsize.tau.c == jumpsize.tau.c &
                    size.jumpsize.rej.df$size == size, ]$tau))
          if (has.no.tau.c.size || has.no.tau.size) {
            rej.df.tmp <- res$rej.prop.df
            rej.df.tmp$p.tau_c <- pexp(rej.df.tmp$tau.c, rej.df.tmp$lambda)
            size.jumpsize.rej.df <- rbind(size.jumpsize.rej.df, rej.df.tmp)
          }
        }
      }
    }
  }
  plot.df <- as.data.table(size.jumpsize.rej.df)
  plot.df <- melt(plot.df, id.vars = c(melt.id.vars, "jumpsize.tau.c"),
                  measure.vars = melt.measure.vars,
                  variable.factor = TRUE)
  plot.df$jumpsize.tau.c <- factor(plot.df$jumpsize.tau.c, levels = jumpsize.tau.c.all,
                                   labels = jumpsize.labels)
  plot.df$size <- factor(plot.df$size, levels = size.all, labels = size.labels)
  plot.df$tau <- as.factor(plot.df$tau)
  legend.names <- format(as.numeric(levels(plot.df$tau)), digits = 4, nsmall = 2)
  plot.x.labels <- paste("q", seq(length(unique(plot.df$p.tau_c))), sep = "")
  g1 <- ggplot(plot.df, aes(x = tau.c, y = value, group = tau,
                            color = tau, linetype = tau)) +
    geom_line(linewidth = 1) +
    geom_hline(yintercept = unique(size.jumpsize.rej.df$alpha),
               colour = 'red', linetype = '3313', linewidth = 1) +
    geom_vline(xintercept = unique(
      plot.df[plot.df$p.tau_c == 1 - unique(size.jumpsize.rej.df$eps), ][["tau.c"]]),
      colour = 'black', linetype = '3313', alpha = 0.35,
      linewidth = 1) +
    scale_x_continuous(breaks = sort(unique(plot.df$tau.c)),
                       labels = plot.x.labels) +
    scale_y_continuous(breaks = seq(0, 1, 0.1), limits = c(0, 1)) +
    theme_bw() +
    theme(legend.position="bottom",
          text = element_text(size = 30),
          axis.text.x = element_text(angle = 90, vjust = 0.5, hjust = 1),
          legend.text.align = 0,
          legend.key.width = unit(0.08, "npc"),
          legend.box.margin = margin(t = -10, r = 25, b = 0, l = 0, unit = "pt")) +
    facet_grid(row = vars(size), cols = vars(jumpsize.tau.c),
               labeller = label_parsed) +
    labs(color  = expression(tau), linetype = expression(tau)) +
    xlab(~ paste(tau["G"])) + ylab("Rejecting proportion") +
    scale_color_hue(labels = legend.names) +
    scale_linetype_discrete(labels = legend.names)

  list.tmp <- list(g1)
  names(list.tmp) <- p
  plots.list <- c(plots.list, list.tmp)
 }
}
```

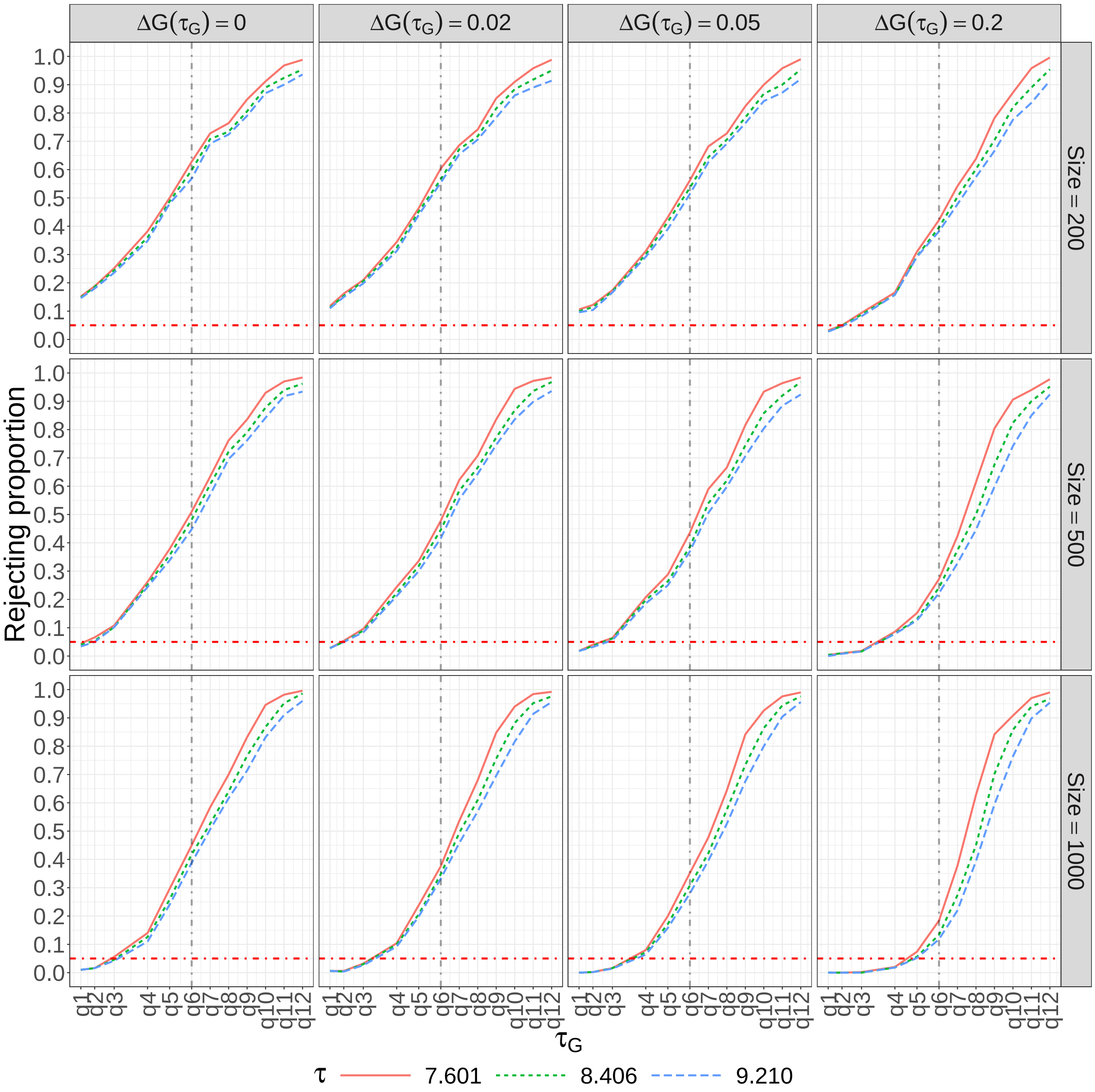

Figure 7: Rejection rate of the null hypothesis of insufficient follow-up for the test based on $\hat{f}_{nh}^{SG}$ with different τ in Setting 1 when $p = 0.2$ (uncured fraction)

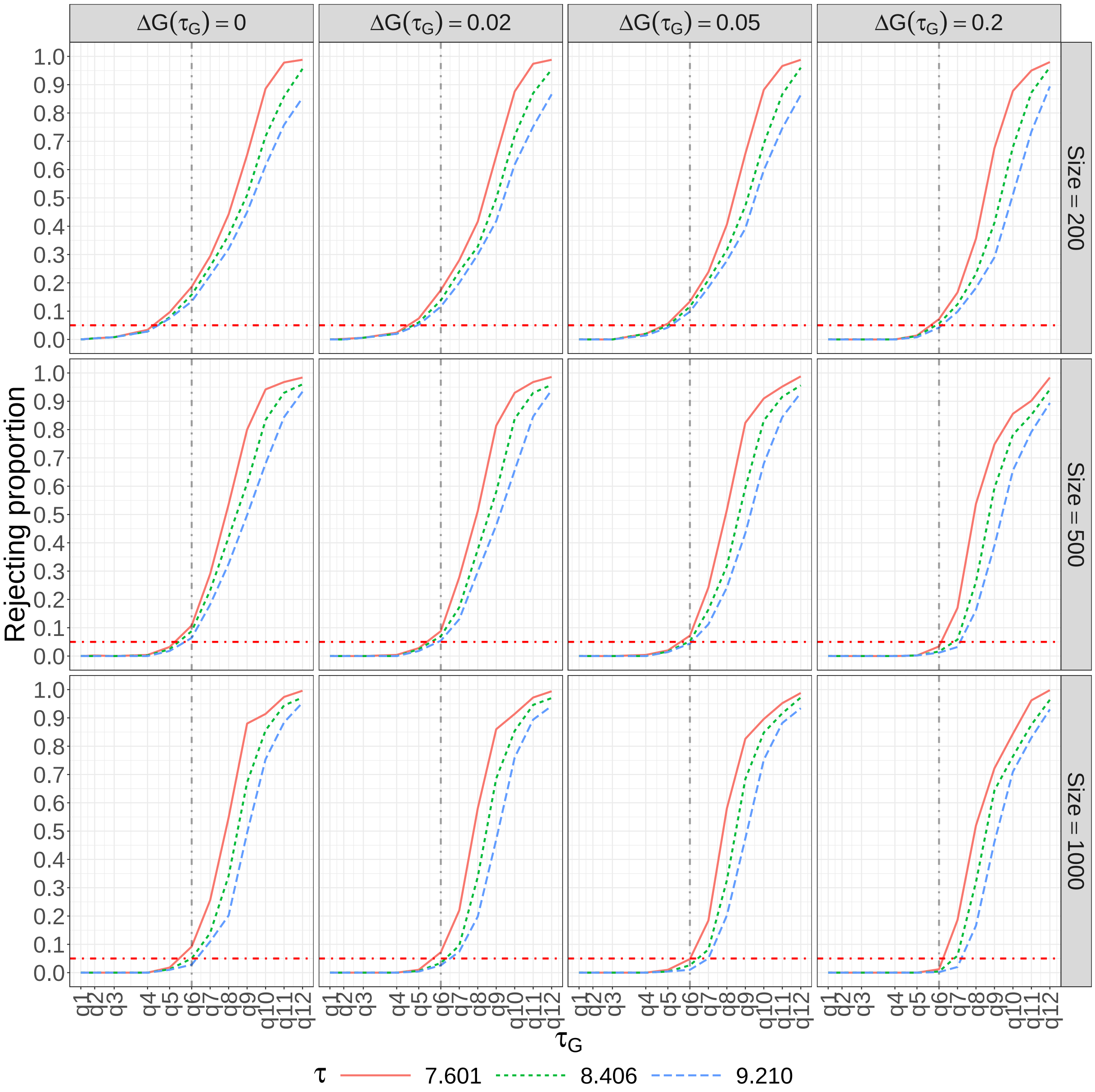

Figure 8: Rejection rate of the null hypothesis of insufficient follow-up for the test based on $\hat{f}_{nh}^{SG}$ with different τ in Setting 1 when $p = 0.6$ (uncured fraction). (Figure S4 in the Supplementary Material)

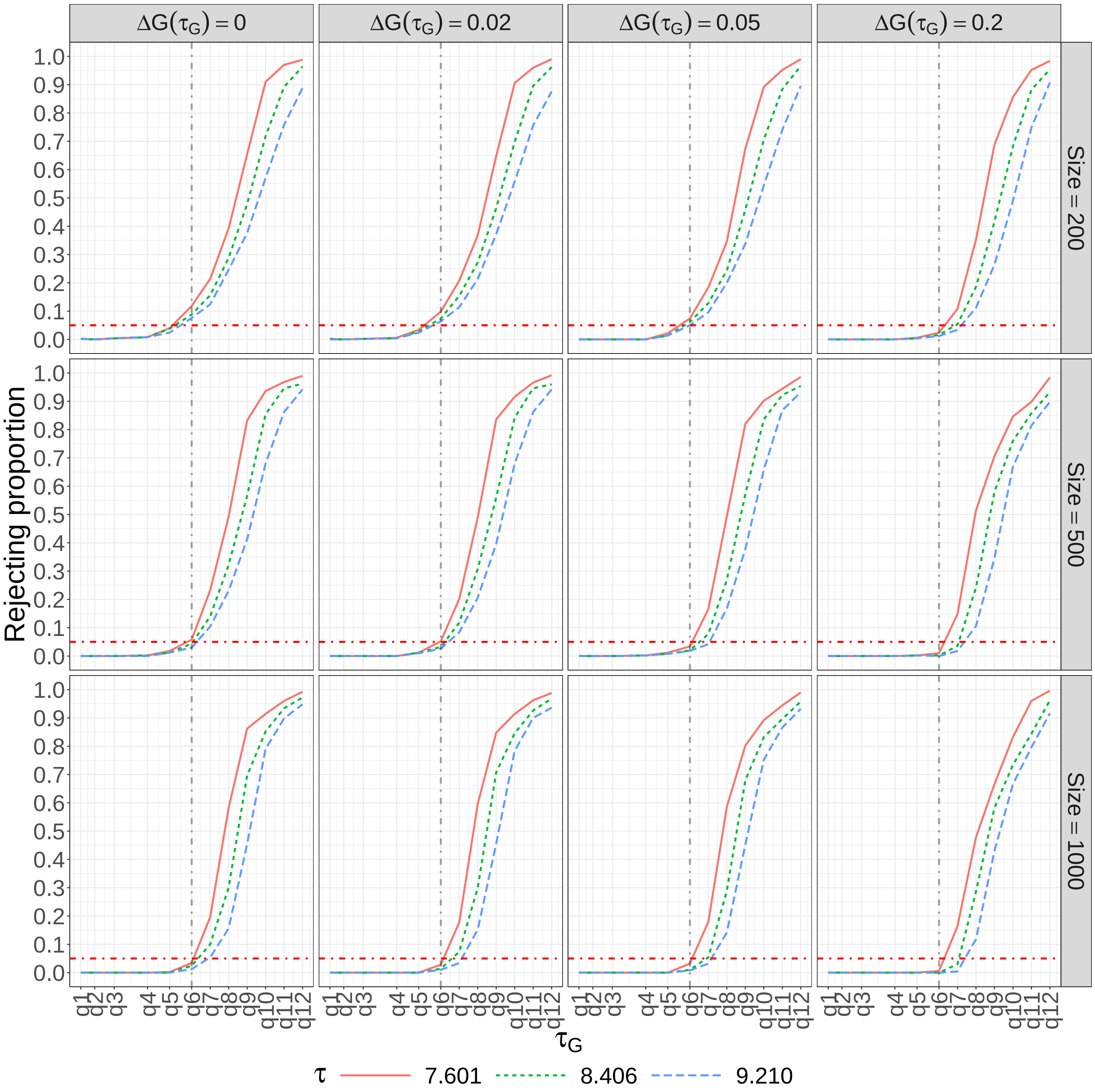

Figure 9: Rejection rate of the null hypothesis of insufficient follow-up for the test based on $\hat{f}_{nh}^{SG}$ with different τ in Setting 1 when $p = 0.8$ (uncured fraction)

Setting 2

This section contains code for generating the figures in Section S1.2.2 of the Supplementary Material using the intermediate results.

```
lambda.all <- c(0.4, 1, 5)
lambda.c.all <- c(0.5)
p.all <- c(0.2, 0.4, 0.8) # cure fraction
size.all <- c(200, 500, 1000)

res.file.path <- "./intermediate_results/setting2"

melt.id.vars <- c("p.tau_c", "tau.c", "size")
melt.measure.vars <- c("sg.H0.is.reject", "gren.H0.is.reject",
                       "alpha.H0.is.reject", "alpha.tilde.H0.is.reject",
                       "q.n.H0.is.reject")
legend.names <- expression(hat(f)[n]^SG, hat(f)[n]^G,
                           hat(alpha)[n], tilde(alpha)[n], Q[n])
lambda.c.labels <- paste('lambda[G] == ', lambda.c.all, sep = "")
lambda.labels <- paste('lambda == ', lambda.all, sep = "")
size.labels <- paste('Size == ', size.all, sep = "")

plots.list <- NULL

for (p in p.all) {
  size.jumpsize.rej.df <- NULL
  for (lambda in lambda.all) {
    for (lambda.c in lambda.c.all) {
      for (size in size.all) {
        rej.df <- NULL
        res.file.subdir <- sprintf("exp_%s_exp_trunc_%s_p_%s_n_%d",
                                   as.character(lambda),
                                   as.character(lambda.c),
                                   as.character(p), size)
        rds.files <- list.files(file.path(res.file.path, res.file.subdir),
                                pattern = "results_grid_no_clip.rds$",
                                full.names = TRUE)
        if (length(rds.files) == 1L) {
          res <- readRDS(rds.files)
          rej.df.tmp <- res$rej.prop.df
          rej.df.tmp$p.tau_c <- pexp(rej.df.tmp$tau.c, rej.df.tmp$lambda)
          rej.df <- rbind(rej.df, rej.df.tmp)
          size.jumpsize.rej.df <- rbind(size.jumpsize.rej.df, rej.df.tmp)
        }
      }
    }
  }
  plot.df <- as.data.table(size.jumpsize.rej.df)
  plot.df <- melt(plot.df, id.vars = c(melt.id.vars, "lambda"),
                  measure.vars = melt.measure.vars,
                  variable.factor = TRUE)
  tau.c.lv <- lapply(lambda.all, function(l) {
    tau.c.unique <- sort(unique(plot.df[plot.df$lambda == l, ]$tau.c))
    return(list("lambda" = l,
                "x.label" = paste("q", seq(length(tau.c.unique)), sep = ""),
                "x.value" = tau.c.unique))
  })
```

```r
  tau.c.labels <- unlist(lapply(tau.c.lv, function(x) x$x.label))
  tau.c.levels <- unlist(lapply(tau.c.lv, function(x) x$x.value))
  plot.df$tau.c <- factor(plot.df$tau.c, levels = tau.c.levels, labels = tau.c.labels)
  plot.df$lambda <- factor(plot.df$lambda, levels = lambda.all,
                           labels = lambda.labels)
  plot.df$size <- factor(plot.df$size, levels = size.all, labels = size.labels)

  g1 <- ggplot(plot.df, aes(x = tau.c, y = value, group = variable,
                            color = variable, linetype = variable)) +
    geom_line(linewidth = 1) +
    geom_hline(yintercept = unique(size.jumpsize.rej.df$alpha),
               colour = 'red', linetype = '3313', linewidth = 1) +
    geom_vline(xintercept = unique(plot.df[plot.df$p.tau_c == 1 - unique(size.jumpsize.rej.df$eps), ][["tau.c"]]),
               colour = 'black', linetype = '3313', alpha = 0.35,
               linewidth = 1) +
    scale_y_continuous(breaks = seq(0, 1, 0.2), limits = c(0, 1)) +
    theme_bw() +
    theme(legend.position="bottom",
          text = element_text(size = 30),
          axis.text.x = element_text(angle = 90, vjust = 0.5, hjust = 1),
          legend.text.align = 0,
          legend.key.width = unit(0.08, "npc"),
          legend.box.margin = margin(t = -10, r = 25, b = 0, l = 0, unit = "pt")) +
    facet_grid(row = vars(size), cols = vars(lambda),
               labeller = label_parsed, scales = "free_x") +
    labs(color  = "Method", linetype = "Method") +
    xlab(~ paste(tau["G"])) + ylab("Rejecting proportion") +
    scale_color_hue(labels = legend.names) +
    scale_linetype_discrete(labels = legend.names)

  list.tmp <- list(g1)
  names(list.tmp) <- p
  plots.list <- c(plots.list, list.tmp)
}
```

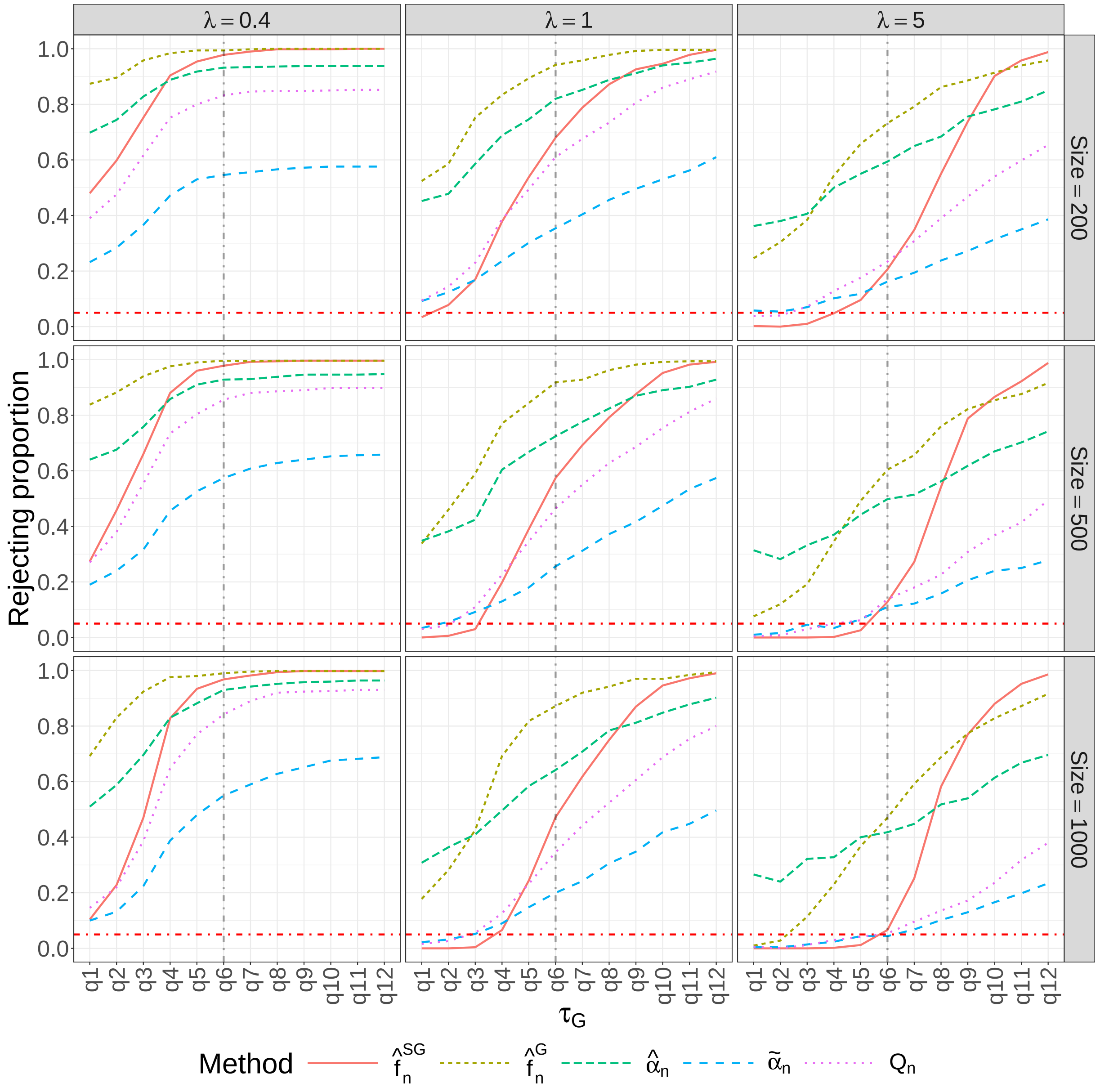

Figure 10: Rejection rate of the null hypothesis of insufficient follow-up for different methods in Setting 2 when p = 0.2 (uncured fraction). (Figure S6 in the Supplementary Material)

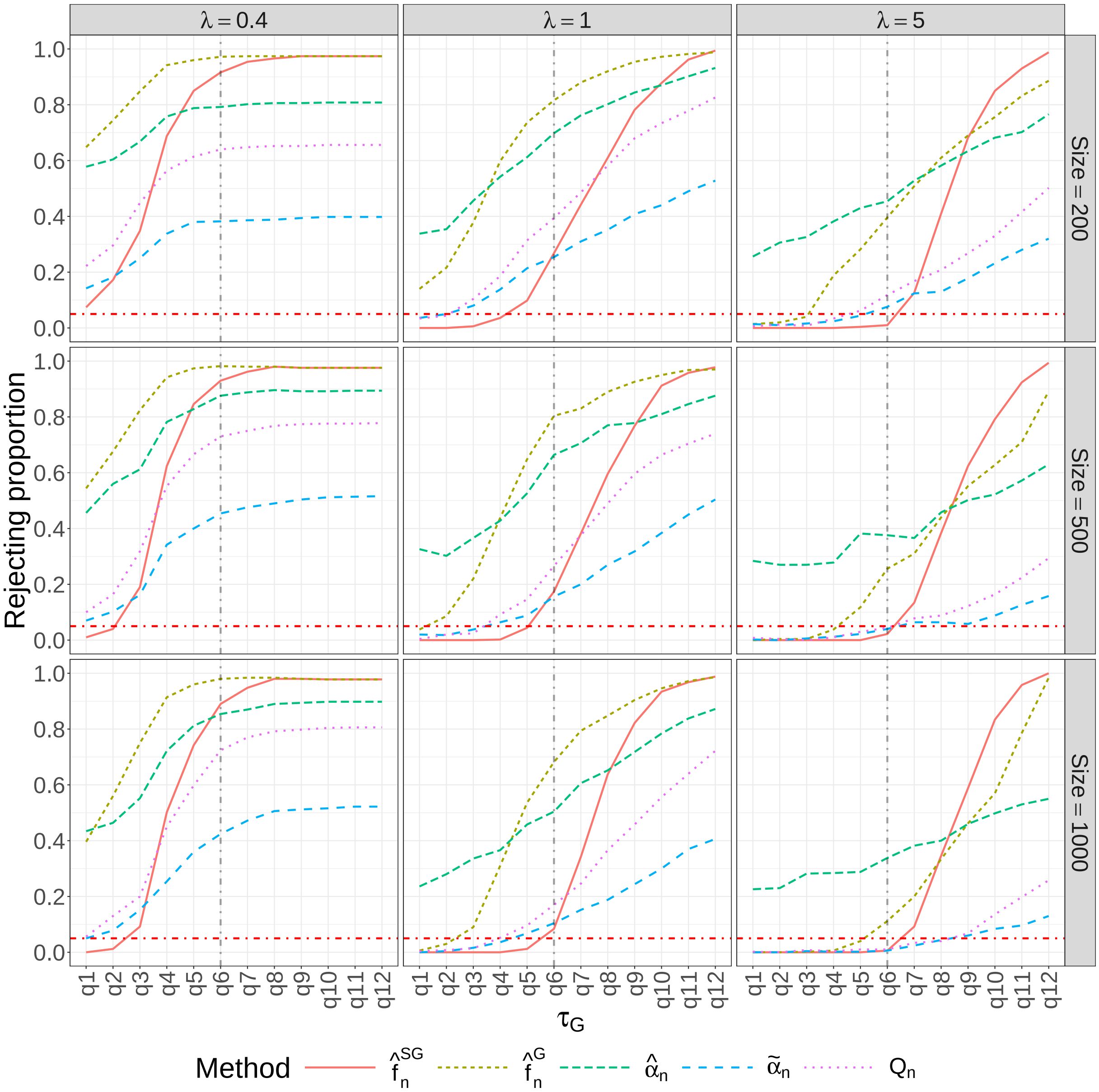

Figure 11: Rejection rate of the null hypothesis of insufficient follow-up for different methods in Setting 2 when p = 0.6 (uncured fraction). (Figure S7 in the Supplementary Material)

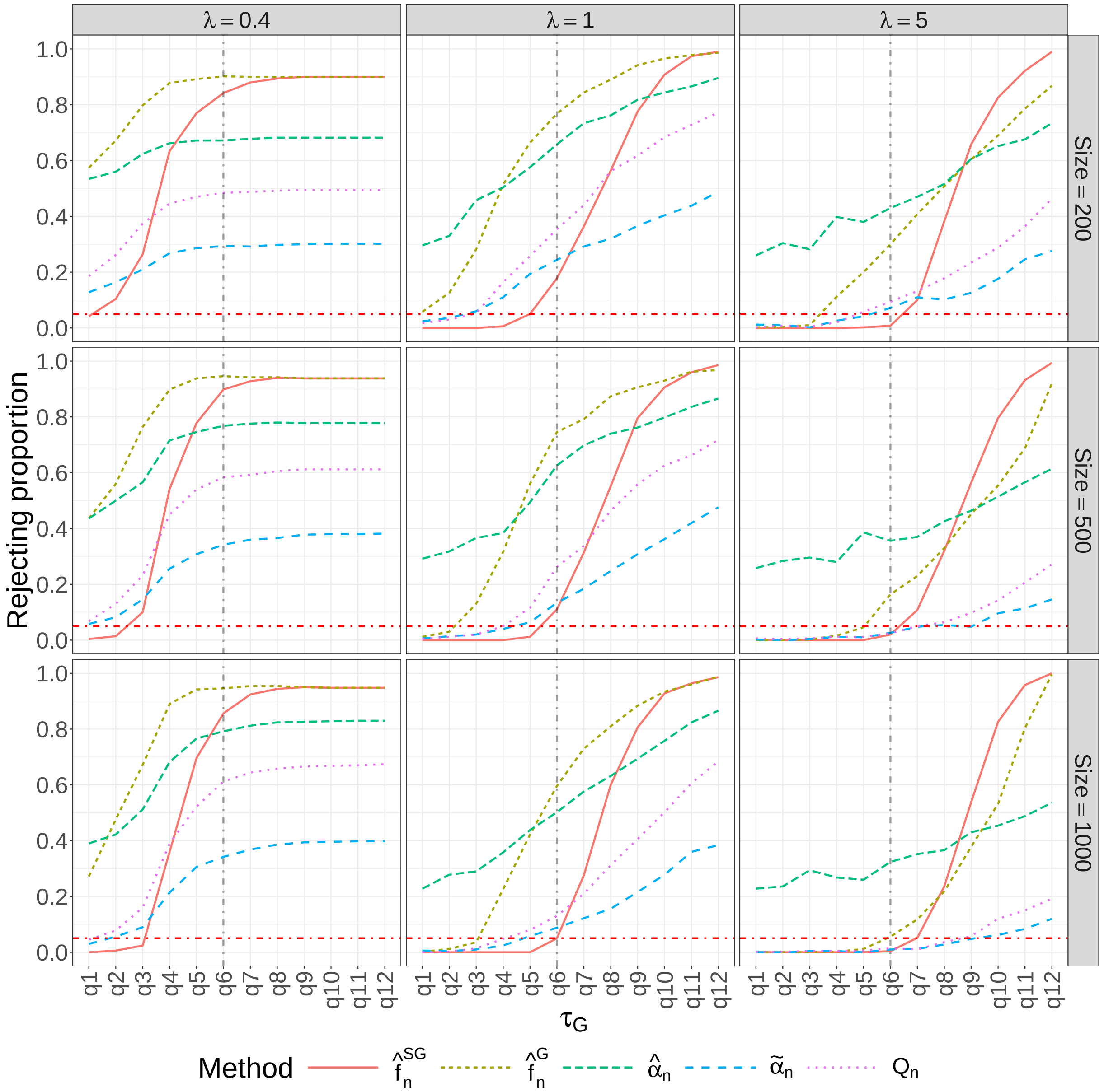

Figure 12: Rejection rate of the null hypothesis of insufficient follow-up for different methods in Setting 2 when p = 0.8 (uncured fraction). (Figure S8 in the Supplementary Material)

Setting 3

This section contains code for generating the figures in Section S1.2.3 of the Supplementary Material using the intermediate results.

```
shape.all <- c(0.5)
scale.all <- c(1.5)
jumpsize.tau.c.all <- c(0, 0.02, 0.05, 0.2)
p.all <- c(0.2, 0.4, 0.8)
size.all <- c(200, 500, 1000)
eps <- 0.01

res.file.path <- "./intermediate_results/setting3"

melt.id.vars <- c("p.tau_c", "tau.c", "size")
melt.measure.vars <- c("sg.H0.is.reject", "gren.H0.is.reject",
                       "alpha.H0.is.reject", "alpha.tilde.H0.is.reject",
                       "q.n.H0.is.reject")
legend.names <- expression(hat(f)[n]^SG, hat(f)[n]^G,
                           hat(alpha)[n], tilde(alpha)[n], Q[n])
lambda.c.labels <- paste('lambda[G] == ', lambda.c.all, sep = "")
lambda.labels <- paste('lambda == ', lambda.all, sep = "")
size.labels <- paste('Size == ', size.all, sep = "")

plots.list <- NULL

for (p in p.all) {
  for (wb.shape in shape.all) {
    for (wb.scale in scale.all) {
      size.jumpsize.rej.df <- NULL
      for (jumpsize.tau.c in jumpsize.tau.c.all) {
        for (size in size.all) {
          rej.df <- NULL
          res.file.subdir <- sprintf("weibull_%s_%s_unif_%s_p_%s_n_%d",
                                     as.character(wb.shape), as.character(wb.scale),
                                     as.character(jumpsize.tau.c),
                                     as.character(p), size)
          rds.files <- list.files(file.path(res.file.path, res.file.subdir),
                                  pattern = sprintf("results_grid_no_clip_%s.rds$", as.character(eps)),
                                  full.names = TRUE)
          if (length(rds.files) == 1L) {
            res <- readRDS(rds.files)
            has.no.tau.c <- all(!(res$rej.prop.df$tau.c %in%
                                    rej.df[rej.df$jumpsize.tau.c == jumpsize.tau.c, ]$tau.c))
            if (has.no.tau.c) {
              rej.df.tmp <- res$rej.prop.df
              rej.df.tmp$vline <- qweibull(1 - rej.df.tmp$eps, rej.df.tmp$shape, rej.df.tmp$scale)
              rej.df.tmp$p.tau_c <- pweibull(rej.df.tmp$tau.c, rej.df.tmp$shape, rej.df.tmp$scale)
              rej.df <- rbind(rej.df, rej.df.tmp)
            }

            has.no.tau.c.size <- all(
              !(res$rej.prop.df$tau.c %in%
                  size.jumpsize.rej.df[
                    size.jumpsize.rej.df$jumpsize.tau.c == jumpsize.tau.c &
                      size.jumpsize.rej.df$size == size, ]$tau.c))
            if (has.no.tau.c.size) {
```

```r
          rej.df.tmp <- res$rej.prop.df
          rej.df.tmp$vline <- qweibull(1 - rej.df.tmp$eps, rej.df.tmp$shape, rej.df.tmp$scale)
          rej.df.tmp$p.tau_c <- pweibull(rej.df.tmp$tau.c, rej.df.tmp$shape, rej.df.tmp$scale)
          size.jumpsize.rej.df <- rbind(size.jumpsize.rej.df, rej.df.tmp)
        }
      }

    }
  }
  plot.df <- as.data.table(size.jumpsize.rej.df)
  plot.df <- melt(plot.df, id.vars = c(melt.id.vars, "jumpsize.tau.c"),
                  measure.vars = melt.measure.vars,
                  variable.factor = TRUE)
  plot.df$jumpsize.tau.c <- factor(plot.df$jumpsize.tau.c, levels = jumpsize.tau.c.all,
                                   labels = jumpsize.labels)
  plot.df$size <- factor(plot.df$size, levels = size.all, labels = size.labels)
  plot.x.labels <- paste("q", seq(length(unique(plot.df$p.tau_c))), sep = "")
  plot.x.labels[plot.x.labels == "q2"] <- ""  # Overlapping labels
  g1 <- ggplot(plot.df, aes(x = tau.c, y = value, group = variable,
                            color = variable, linetype = variable)) +
    geom_line(linewidth = 1) +
    geom_hline(yintercept = unique(size.jumpsize.rej.df$alpha),
               colour = 'red', linetype = '3313', linewidth = 1) +
    geom_vline(xintercept = unique(plot.df$vline),
               colour = 'black', linetype = '3313', alpha = 0.35, linewidth = 1) +
    scale_x_continuous(breaks = sort(unique(plot.df$tau.c)),
                       labels = plot.x.labels) +
    scale_y_continuous(breaks = seq(0, 1, 0.2), limits = c(0, 1)) +
    theme_bw() +
    theme(legend.position="bottom",
          text = element_text(size = 30),
          axis.text.x = element_text(angle = 90, vjust = 0.5, hjust = 1),
          legend.text.align = 0,
          legend.key.width = unit(0.08, "npc"),
          legend.box.margin = margin(t = -10, r = 25, b = 0, l = 0, unit = "pt")) +
    facet_grid(row = vars(size), cols = vars(jumpsize.tau.c),
               labeller = label_parsed) +
    labs(color  = "Method", linetype = "Method") +
    xlab(~ paste(tau["G"])) + ylab("Rejecting proportion") +
    scale_color_hue(labels = legend.names) +
    scale_linetype_discrete(labels = legend.names)

  list.tmp <- list(g1)
  names(list.tmp) <- p
  plots.list <- c(plots.list, list.tmp)
    }
  }
}
```

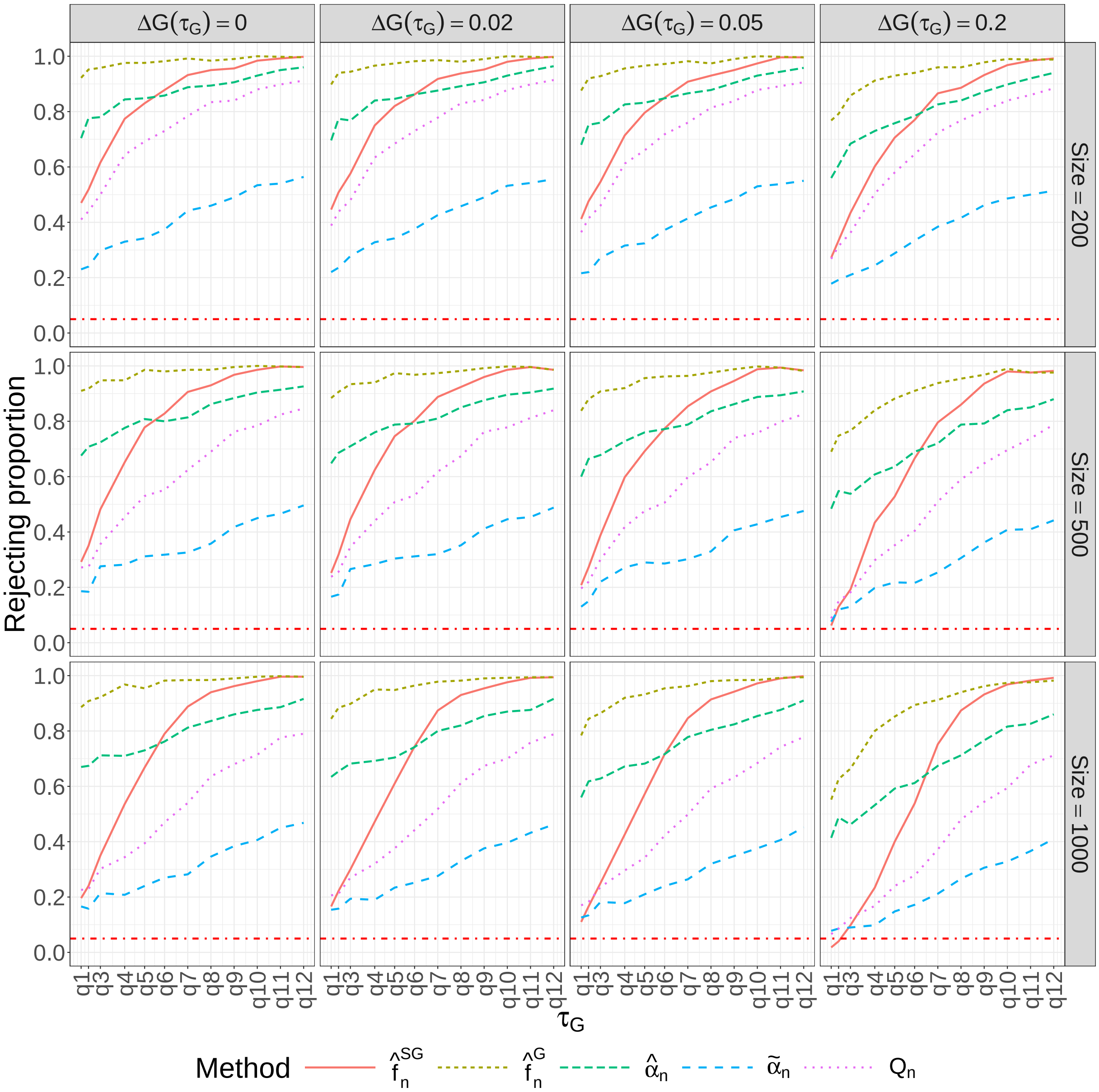

Figure 13: Rejection rate of the null hypothesis of insufficient follow-up for different methods in Setting 3 when p = 0.2 (uncured fraction). (Figure S9 in the Supplementary Material)

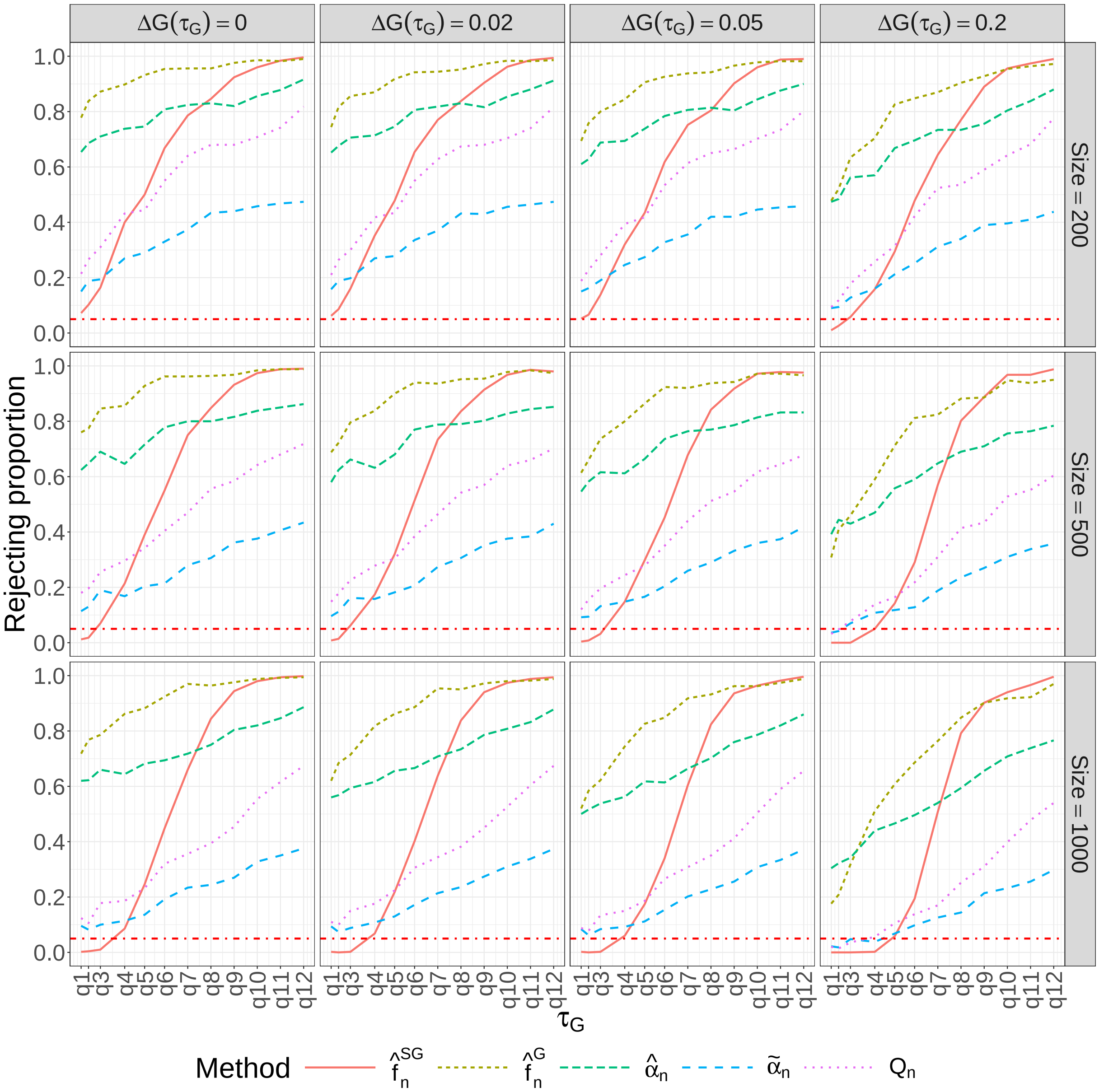

Figure 14: Rejection rate of the null hypothesis of insufficient follow-up for different methods in Setting 3 when p = 0.6 (uncured fraction). (Figure S10 in the Supplementary Material)

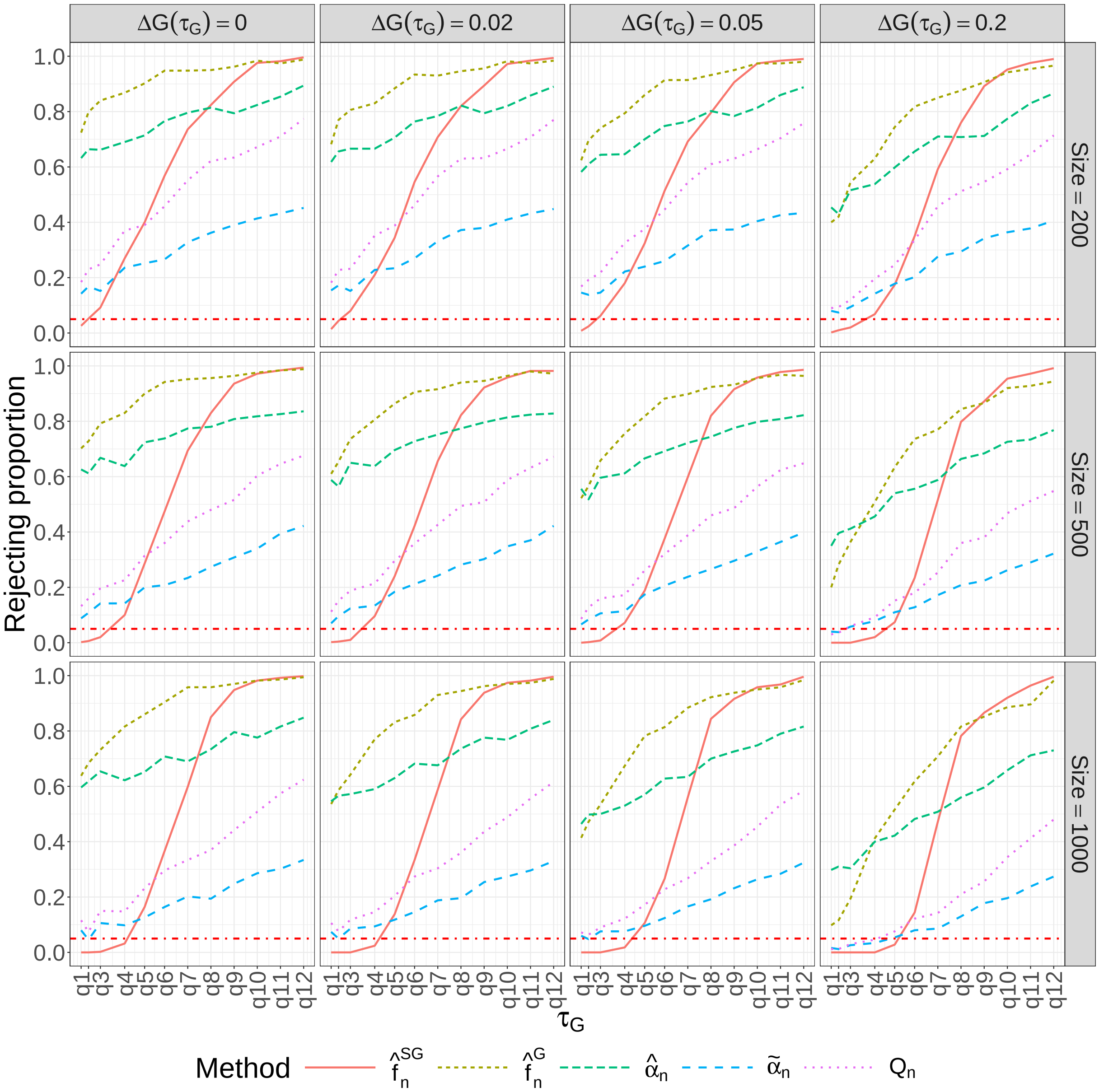

Figure 15: Rejection rate of the null hypothesis of insufficient follow-up for different methods in Setting 3 when p = 0.8 (uncured fraction). (Figure S11 in the Supplementary Material)

Changing ϵ from 0.01 to 0.005

This subsection contains code for generating the figures in Section S1.2.3 to study the choice of ϵ.

```
shape.all <- c(0.5)
scale.all <- c(1.5)

jumpsize.tau.c.all <- c(0, 0.02, 0.05, 0.2)
p.all <- c(0.2, 0.4, 0.8)
size.all <- c(200, 500, 1000)
eps.all <- c(0.01, 0.005)


res.file.path <- "./intermediate_results/setting3"

melt.id.vars <- c("p.tau_c", "tau.c", "size", "vline", "eps")
melt.measure.vars <- c(
  "sg.H0.is.reject"
)
legend.names <- expression(hat(f)[n]^G, hat(f)[n]^SG,
                           hat(alpha)[n], tilde(alpha)[n], Q[n])
jumpsize.labels <- paste('Delta*G(tau[G]) == ', jumpsize.tau.c.all, sep = "")
size.labels <- paste('Size == ', size.all, sep = "")

plots.list <- NULL

for (p in p.all) {
  for (wb.shape in shape.all) {
    for (wb.scale in scale.all) {
      size.jumpsize.rej.df <- NULL
      for (jumpsize.tau.c in jumpsize.tau.c.all) {
        for (size in size.all) {
          rej.df <- NULL
          res.file.subdir <- sprintf("weibull_%s_%s_unif_%s_p_%s_n_%d",
                                     as.character(wb.shape), as.character(wb.scale),
                                     as.character(jumpsize.tau.c),
                                     as.character(p), size)
          for (eps in eps.all) {
            rds.files <- list.files(
              file.path(res.file.path, res.file.subdir),
              pattern = sprintf("results_grid_no_clip_%s.rds$", as.character(eps)),
              full.names = TRUE)
            if (length(rds.files) == 1L) {
              res <- readRDS(rds.files)
              has.no.tau.c <- all(!(res$rej.prop.df$tau.c %in%
                                    rej.df[rej.df$jumpsize.tau.c == jumpsize.tau.c, ]$tau.c))
              has.no.eps <- all(!(res$rej.prop.df$eps %in%
                                  rej.df[rej.df$jumpsize.tau.c == jumpsize.tau.c, ]$eps))
              if (has.no.tau.c || has.no.eps) {
                rej.df.tmp <- res$rej.prop.df
                rej.df.tmp$vline <- qweibull(1 - rej.df.tmp$eps, rej.df.tmp$shape, rej.df.tmp$scale)
                rej.df.tmp$p.tau_c <- pweibull(rej.df.tmp$tau.c, rej.df.tmp$shape, rej.df.tmp$scale)
                rej.df <- rbind(rej.df, rej.df.tmp)
              }

              has.no.tau.c.size <- all(
```

```
              !(res$rej.prop.df$tau.c %in%
                  size.jumpsize.rej.df[
                    size.jumpsize.rej.df$jumpsize.tau.c == jumpsize.tau.c &
                      size.jumpsize.rej.df$size == size, ]$tau.c))
            has.no.eps.size <- all(
              !(res$rej.prop.df$eps %in%
                  size.jumpsize.rej.df[
                    size.jumpsize.rej.df$jumpsize.tau.c == jumpsize.tau.c &
                      size.jumpsize.rej.df$size == size, ]$eps))
            if (has.no.tau.c.size || has.no.eps.size) {
              rej.df.tmp <- res$rej.prop.df
              rej.df.tmp$vline <- qweibull(1 - rej.df.tmp$eps, rej.df.tmp$shape, rej.df.tmp$scale)
              rej.df.tmp$p.tau_c <- pweibull(rej.df.tmp$tau.c, rej.df.tmp$shape, rej.df.tmp$scale)
              size.jumpsize.rej.df <- rbind(size.jumpsize.rej.df, rej.df.tmp)
            }
          }
        }
      }
    }
    plot.df <- as.data.table(size.jumpsize.rej.df)
    plot.df <- melt(plot.df, id.vars = c(melt.id.vars, "jumpsize.tau.c"),
                    measure.vars = melt.measure.vars,
                    variable.factor = TRUE)
    plot.df$jumpsize.tau.c <- factor(plot.df$jumpsize.tau.c, levels = jumpsize.tau.c.all,
                                     labels = jumpsize.labels)
    plot.df$size <- factor(plot.df$size, levels = size.all, labels = size.labels)
    plot.df$eps <- as.factor(plot.df$eps)
    plot.x.labels <- paste("q", seq(length(unique(plot.df$p.tau_c))), sep = "")
    plot.x.labels[plot.x.labels == "q2"] <- ""  # Overlapping labels
    legend.names <- sapply(levels(plot.df$eps), function(e) bquote(epsilon==.(as.numeric(e))))
    g1 <- ggplot(plot.df, aes(x = tau.c, y = value, group = eps,
                              color = eps, linetype = eps)) +
      geom_line(linewidth = 1) +
      geom_hline(yintercept = unique(size.jumpsize.rej.df$alpha),
                 colour = 'red', linetype = '3313', linewidth = 1) +
      scale_x_continuous(breaks = sort(unique(plot.df$tau.c)),
                         labels = plot.x.labels) +
      scale_y_continuous(breaks = seq(0, 1, 0.2), limits = c(0, 1)) +
      theme_bw() +
      theme(legend.position="bottom",
            text = element_text(size = 30),
            axis.text.x = element_text(angle = 90, vjust = 0.5, hjust = 1),
            legend.text.align = 0,
            legend.key.width = unit(0.08, "npc"),
            legend.box.margin = margin(t = -10, r = 25, b = 0, l = 0, unit = "pt")) +
      facet_grid(row = vars(size), cols = vars(jumpsize.tau.c),
                 labeller = label_parsed) +
      labs(color  = "", linetype = "") +
      xlab(~ paste(tau["G"])) + ylab("Rejecting proportion") +
      scale_color_hue(labels = legend.names) +
      scale_linetype_discrete(labels = legend.names)

    list.tmp <- list(g1)
    names(list.tmp) <- p
    plots.list <- c(plots.list, list.tmp)
  }
}
```

```
}
```

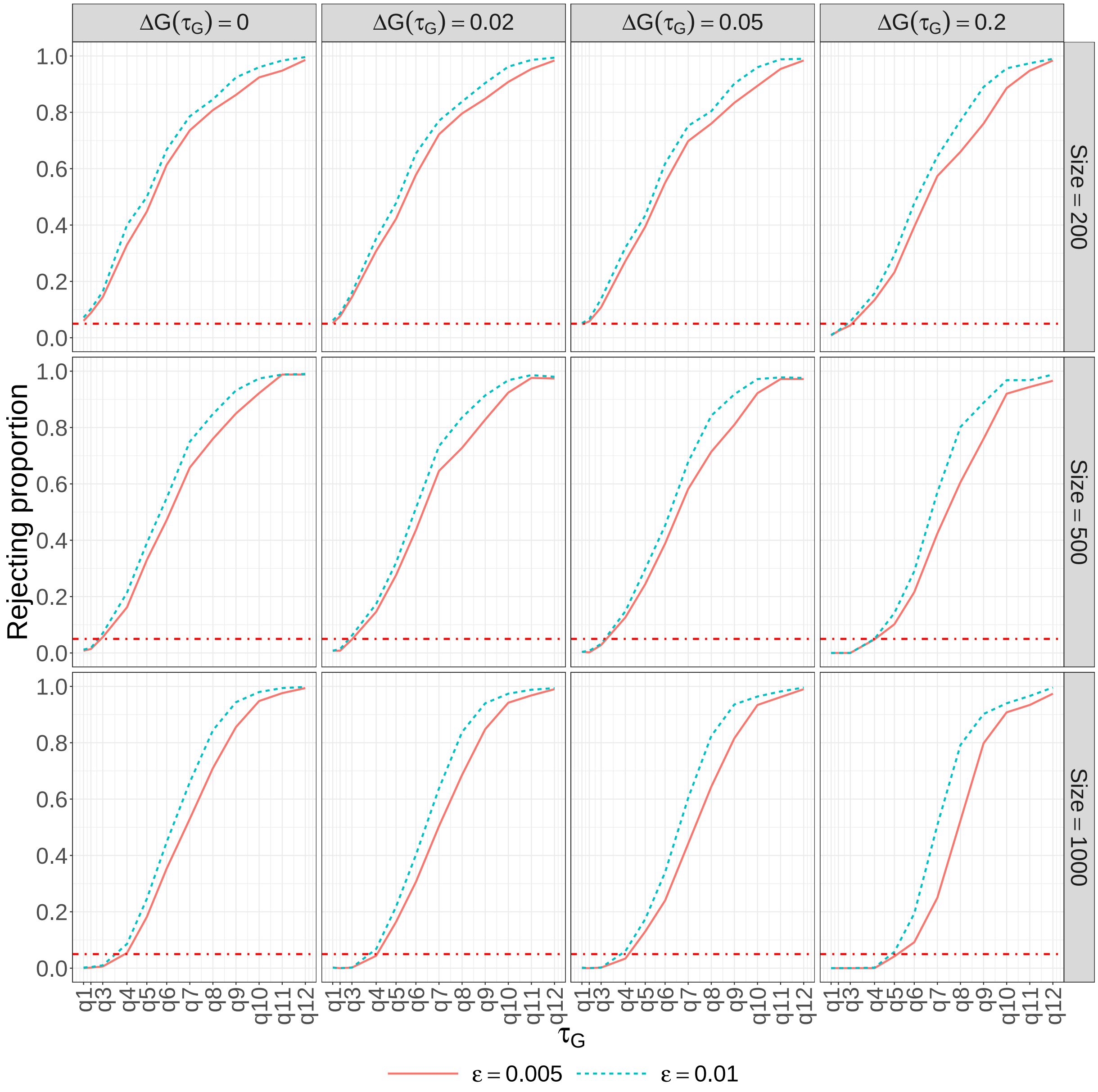

Figure 16: Rejection rate of insufficient follow-up for the test based on $\hat{f}_{nh}^{SG}$ in Setting 3 when $p = 0.6$ (uncured fraction). q_6 corresponds to $q_{0.99}$; and $q_{0.995}$ locates between q_7 and q_8. (Figure S12 in the Supplementary Material)

Setting 4

This section contains code for generating the figures in Section 4.2 of the main manuscript and in Section S1.2.4 of the Supplementary Material using the intermediate results.

```
lambda.all <- c(1)
p.tau0.all <- c(0.99)
jumpsize.tau.c.all <- c(0, 0.02, 0.05, 0.2)
p.all <- 0.4
size.all <- c(200, 500, 1000)

res.file.path <- "./intermediate_results/setting4"


melt.id.vars <- c("p.tau_c", "tau.c", "size")
melt.measure.vars <- c("sg.H0.is.reject", "gren.H0.is.reject",
                       "alpha.H0.is.reject", "alpha.tilde.H0.is.reject",
                       "q.n.H0.is.reject")
legend.names <- expression(hat(f)[n]^SG, hat(f)[n]^G,
                           hat(alpha)[n], tilde(alpha)[n], Q[n])
jumpsize.labels <- paste('Delta*G(tau[G]) == ', jumpsize.tau.c.all, sep = "")
size.labels <- paste('Size == ', size.all, sep = "")

plots.list <- NULL
all.rej.df <- NULL
for (p in p.all) {
  for (lambda in lambda.all) {
    for (p.tau0 in p.tau0.all) {
      size.jumpsize.rej.df <- NULL
      for (jumpsize.tau.c in jumpsize.tau.c.all) {
        for (size in size.all) {
          rej.df <- NULL
          res.file.subdir <- sprintf("texp_%s_%s_unif_%s_p_%s_n_%d",
                                     as.character(lambda), as.character(p.tau0),
                                     as.character(jumpsize.tau.c),
                                     as.character(p), size)
          rds.files <- list.files(file.path(res.file.path, res.file.subdir),
                                  pattern = "results_grid_no_clip.rds$",
                                  full.names = TRUE)
          if (length(rds.files) == 1L) {
            res <- readRDS(rds.files)
            has.no.tau.c <- all(!(res$rej.prop.df$tau.c %in%
                                    rej.df[rej.df$jumpsize.tau.c == jumpsize.tau.c, ]$tau.c))
            if (has.no.tau.c) {
              rej.df.tmp <- res$rej.prop.df
              p.tau_c.tmp <- 1 + pexp(rej.df.tmp$tau.c, rej.df.tmp$lambda)
              tau0.tmp <- qexp(rej.df.tmp$p.tau0, rej.df.tmp$lambda)
              p.tau_c.tmp[rej.df.tmp$tau.c < tau0.tmp] <- ptexp(
                rej.df.tmp[rej.df.tmp$tau.c < tau0.tmp, ]$tau.c,
                rej.df.tmp[rej.df.tmp$tau.c < tau0.tmp, ]$lambda,
                tau0.tmp[rej.df.tmp$tau.c < tau0.tmp])
              rej.df.tmp$p.tau_c <- p.tau_c.tmp

              rej.df <- rbind(rej.df, rej.df.tmp)
            }
```

```
        has.no.tau.c.size <- all(
          !(res$rej.prop.df$tau.c %in%
              size.jumpsize.rej.df[
                size.jumpsize.rej.df$jumpsize.tau.c == jumpsize.tau.c &
                  size.jumpsize.rej.df$size == size, ]$tau.c))
        if (has.no.tau.c.size) {
          rej.df.tmp <- res$rej.prop.df
          p.tau_c.tmp <- 1 + pexp(rej.df.tmp$tau.c, rej.df.tmp$lambda)
          tau0.tmp <- qexp(rej.df.tmp$p.tau0, rej.df.tmp$lambda)
          p.tau_c.tmp[rej.df.tmp$tau.c < tau0.tmp] <- ptexp(
            rej.df.tmp[rej.df.tmp$tau.c < tau0.tmp, ]$tau.c,
            rej.df.tmp[rej.df.tmp$tau.c < tau0.tmp, ]$lambda,
            tau0.tmp[rej.df.tmp$tau.c < tau0.tmp])
          rej.df.tmp$p.tau_c <- p.tau_c.tmp

          size.jumpsize.rej.df <- rbind(size.jumpsize.rej.df, rej.df.tmp)
          all.rej.df <- rbind(all.rej.df, rej.df.tmp)
        }
      }

    }
  }
plot.df <- as.data.table(size.jumpsize.rej.df)
plot.df <- melt(plot.df, id.vars = c(melt.id.vars, "jumpsize.tau.c"),
                measure.vars = melt.measure.vars,
                variable.factor = TRUE)
plot.df$jumpsize.tau.c <- factor(plot.df$jumpsize.tau.c, levels = jumpsize.tau.c.all,
                                 labels = jumpsize.labels)
plot.df$size <- factor(plot.df$size, levels = size.all, labels = size.labels)

plot.x.labels <- paste("q", seq(length(unique(plot.df$p.tau_c))), sep = "")
plot.x.labels[c(7, 9, 11)] <- ""
g1 <- ggplot(plot.df, aes(x = tau.c, y = value, group = variable,
                          color = variable, linetype = variable)) +
  geom_line(linewidth = 1) +
  geom_hline(yintercept = unique(size.jumpsize.rej.df$alpha),
             colour = 'red', linetype = '3313', linewidth = 1) +
  geom_vline(xintercept = unique(plot.df[plot.df$p.tau_c == 1 - unique(size.jumpsize.rej.df$eps), ][["tau.c"]]),
             colour = 'black', linetype = '3313', alpha = 0.35,
             linewidth = 1) +
  scale_x_continuous(breaks = sort(unique(plot.df$tau.c)),
                     labels = plot.x.labels) +
  scale_y_continuous(breaks = seq(0, 1, 0.1), limits = c(0, 1)) +
  theme_bw() +
  theme(legend.position="bottom",
        text = element_text(size = 30),
        axis.text.x = element_text(angle = 90, vjust = 0.5, hjust = 1),
        legend.text.align = 0,
        legend.key.width = unit(0.08, "npc"),
        legend.box.margin = margin(t = -10, r = 25, b = 0, l = 0, unit = "pt")) +
  facet_grid(row = vars(size), cols = vars(jumpsize.tau.c),
             labeller = label_parsed) +
  labs(color  = "Method", linetype = "Method") +
  xlab(~ paste(tau["G"])) + ylab("Rejecting proportion") +
  scale_color_hue(labels = legend.names) +
  scale_linetype_discrete(labels = legend.names)
```

```
      list.tmp <- list(g1)
      names(list.tmp) <- p
      plots.list <- c(plots.list, list.tmp)
    }
  }
}
```

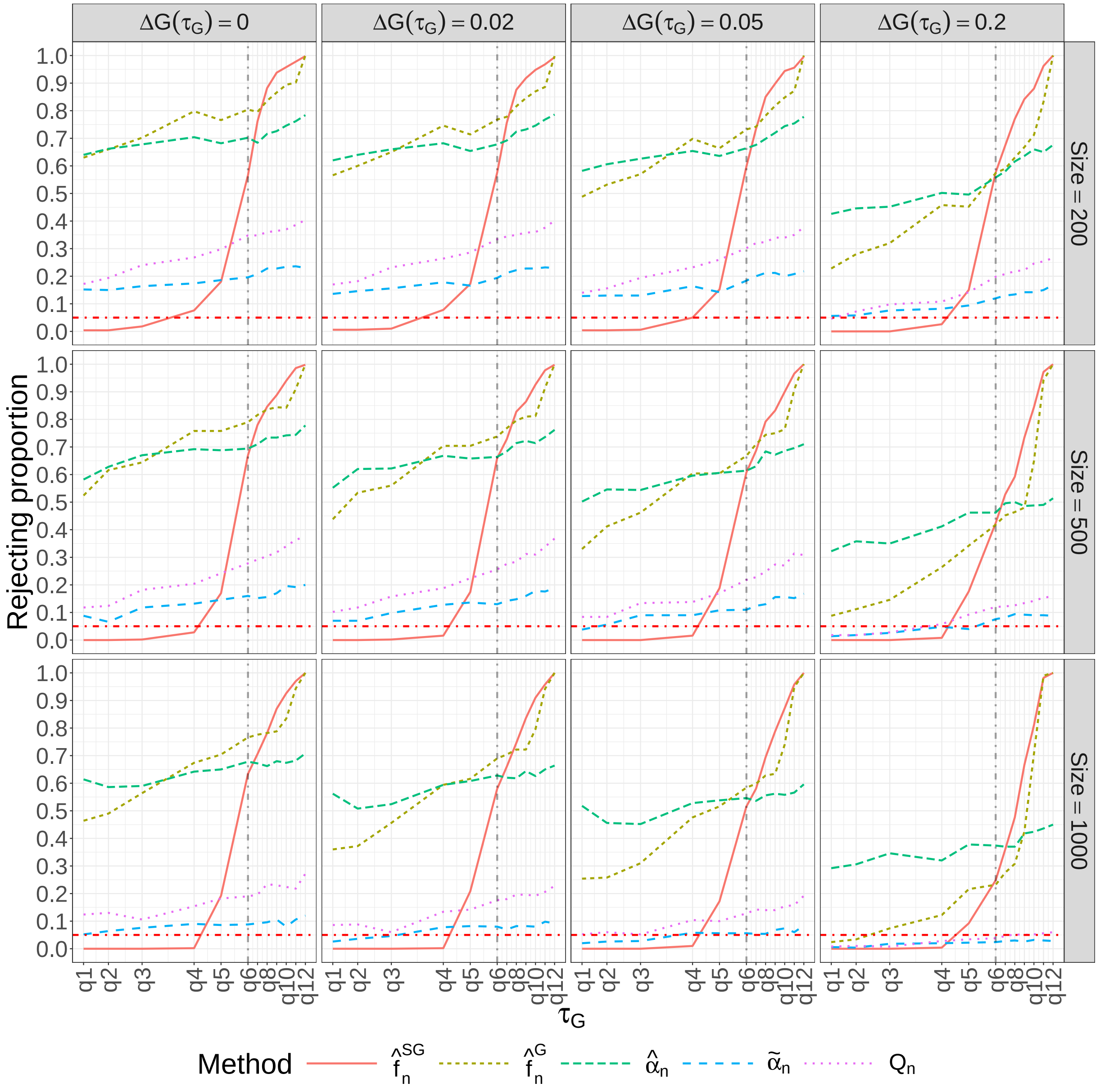

Figure 17: Rejection rate of the null hypothesis of insufficient follow-up for different methods in Setting 4 when p = 0.6 (uncured fraction). (Figure S13 in the Supplementary Material)

```
lambda.all <- c(1)
p.tau0.all <- c(0.99)
jumpsize.tau.c.all <- c(0, 0.02, 0.05, 0.2)
p.all <- 0.4
```

```r
size.all <- c(200, 500, 1000)

res.file.path <- "./intermediate_results/setting4"


melt.id.vars <- c("p.tau_c", "tau.c", "size")
melt.measure.vars <- c("sg.H0.is.reject", "gren.H0.is.reject",
                       "alpha.H0.is.reject", "alpha.tilde.H0.is.reject",
                       "q.n.H0.is.reject")
legend.names <- expression(hat(f)[n]^SG, hat(f)[n]^G,
                           hat(alpha)[n], tilde(alpha)[n], Q[n])
jumpsize.labels <- paste('Delta*G(tau[G]) == ', jumpsize.tau.c.all, sep = "")
size.labels <- paste('Size == ', size.all, sep = "")

plots.list <- NULL
all.rej.df <- NULL
for (p in p.all) {
  for (lambda in lambda.all) {
    for (p.tau0 in p.tau0.all) {
      size.jumpsize.rej.df <- NULL
      for (jumpsize.tau.c in jumpsize.tau.c.all) {
        for (size in size.all) {
          rej.df <- NULL
          res.file.subdir <- sprintf("texp_%s_%s_unif_%s_p_%s_n_%d",
                                     as.character(lambda), as.character(p.tau0),
                                     as.character(jumpsize.tau.c),
                                     as.character(p), size)
          rds.files.suff.tau1 <- list.files(file.path(res.file.path, res.file.subdir),
                                            pattern = "results_grid_no_clip_extra_1.rds$",
                                            full.names = TRUE)
          rds.files.suff.tau2 <- list.files(file.path(res.file.path, res.file.subdir),
                                            pattern = "results_grid_no_clip_extra_1.25.rds$",
                                            full.names = TRUE)
          rds.files.suff.tau3 <- list.files(file.path(res.file.path, res.file.subdir),
                                            pattern = "results_grid_no_clip_extra_1.5.rds$",
                                            full.names = TRUE)
          for (rds.files in c(rds.files.suff.tau1, rds.files.suff.tau2, rds.files.suff.tau3)) {
            if (length(rds.files) == 1L) {
              res <- readRDS(rds.files)
              has.no.tau.c <- all(!(res$rej.prop.df$tau.c %in%
                                      rej.df[rej.df$jumpsize.tau.c == jumpsize.tau.c, ]$tau.c))
              has.no.tau <- all(!(res$rej.prop.df$tau.suff %in%
                                    rej.df[rej.df$jumpsize.tau.c == jumpsize.tau.c, ]$tau))
              if (has.no.tau.c || has.no.tau) {
                rej.df.tmp <- res$rej.prop.df
                if ("tau.suff" %in% colnames(rej.df.tmp)) {
                  rej.df.tmp$tau <- rej.df.tmp$tau.suff
                  rej.df.tmp$tau.suff <- NULL
                }
                p.tau_c.tmp <- 1 + pexp(rej.df.tmp$tau.c, rej.df.tmp$lambda)
                tau0.tmp <- qexp(rej.df.tmp$p.tau0, rej.df.tmp$lambda)
                p.tau_c.tmp[rej.df.tmp$tau.c < tau0.tmp] <- ptexp(
                  rej.df.tmp[rej.df.tmp$tau.c < tau0.tmp, ]$tau.c,
                  rej.df.tmp[rej.df.tmp$tau.c < tau0.tmp, ]$lambda,
                  tau0.tmp[rej.df.tmp$tau.c < tau0.tmp])
                rej.df.tmp$p.tau_c <- p.tau_c.tmp
```

```r
        rej.df <- rbind(rej.df, rej.df.tmp)
      }

      has.no.tau.c.size <- all(
        !(res$rej.prop.df$tau.c %in%
            size.jumpsize.rej.df[
              size.jumpsize.rej.df$jumpsize.tau.c == jumpsize.tau.c &
                size.jumpsize.rej.df$size == size, ]$tau.c))
      has.no.tau.size <- all(
        !(res$rej.prop.df$tau.suff %in%
            size.jumpsize.rej.df[
              size.jumpsize.rej.df$jumpsize.tau.c == jumpsize.tau.c &
                size.jumpsize.rej.df$size == size, ]$tau))
      if (has.no.tau.c.size || has.no.tau.size) {
        rej.df.tmp <- res$rej.prop.df
        if ("tau.suff" %in% colnames(rej.df.tmp)) {
          rej.df.tmp$tau <- rej.df.tmp$tau.suff
          rej.df.tmp$tau.suff <- NULL
        }
        p.tau_c.tmp <- 1 + pexp(rej.df.tmp$tau.c, rej.df.tmp$lambda)
        tau0.tmp <- qexp(rej.df.tmp$p.tau0, rej.df.tmp$lambda)
        p.tau_c.tmp[rej.df.tmp$tau.c < tau0.tmp] <- ptexp(
          rej.df.tmp[rej.df.tmp$tau.c < tau0.tmp, ]$tau.c,
          rej.df.tmp[rej.df.tmp$tau.c < tau0.tmp, ]$lambda,
          tau0.tmp[rej.df.tmp$tau.c < tau0.tmp])
        rej.df.tmp$p.tau_c <- p.tau_c.tmp

        size.jumpsize.rej.df <- rbind(size.jumpsize.rej.df, rej.df.tmp)
      }
      has.no.tau.c.all <- all(!(res$rej.prop.df$tau.c %in%
                                 all.rej.df[all.rej.df$jumpsize.tau.c == jumpsize.tau.c &
                                              all.rej.df$size == size, ]$tau.c))
      has.no.tau.size.all <- all(
        !(res$rej.prop.df$tau.suff %in%
            all.rej.df[
              all.rej.df$jumpsize.tau.c == jumpsize.tau.c &
                all.rej.df$size == size, ]$tau))

      if (has.no.tau.c.all || has.no.tau.size.all) {
        rej.df.tmp <- res$rej.prop.df
        if ("tau.suff" %in% colnames(rej.df.tmp)) {
          rej.df.tmp$tau <- rej.df.tmp$tau.suff
          rej.df.tmp$tau.suff <- NULL
        }
        p.tau_c.tmp <- 1 + pexp(rej.df.tmp$tau.c, rej.df.tmp$lambda)
        tau0.tmp <- qexp(rej.df.tmp$p.tau0, rej.df.tmp$lambda)
        p.tau_c.tmp[rej.df.tmp$tau.c < tau0.tmp] <- ptexp(
          rej.df.tmp[rej.df.tmp$tau.c < tau0.tmp, ]$tau.c,
          rej.df.tmp[rej.df.tmp$tau.c < tau0.tmp, ]$lambda,
          tau0.tmp[rej.df.tmp$tau.c < tau0.tmp])
        rej.df.tmp$p.tau_c <- p.tau_c.tmp
        all.rej.df <- rbind(all.rej.df, rej.df.tmp)
      }
    }
  }

}
```

```
      }
    }
  }
}
```

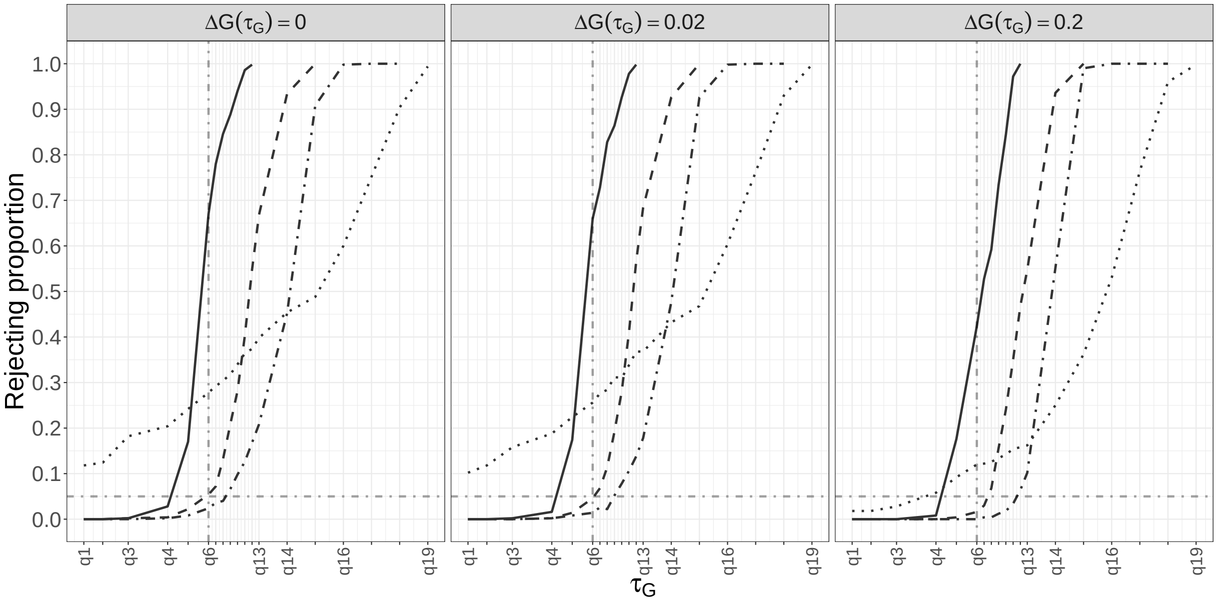

Figure 18: Rejection rate of the null hypothesis of insufficient follow-up for different methods (solid: $\hat{f}_{nh}^{SG}$ with $\tau_1 \approx 4.5569$, dashed: $\hat{f}_{nh}^{SG}$ with $\tau_2 = 1.25\tau_1$, dash-dotted: $\hat{f}_{nh}^{SG}$ with $\tau_3 = 1.5\tau_1$, dotted: Q_n) in Setting 4 with $n = 500$, $p = 0.6$ and $\Delta G(\tau_G) = 0$ (left), $\Delta G(\tau_G) = 0.02$ (center), and $\Delta G(\tau_G) = 0.2$ (right). (Figure 4 in the main manuscript)

Setting 5

This section contains code for generating the figures in Section S1.2.5 of the Supplementary Material using the intermediate results.

```
lambda.all <- c(5)
p.tau0.all <- c(0.99)
lambda.c.all <- c(0.5, 3)
p.all <- 0.4
size.all <- c(200, 500, 1000)

res.file.path <- "./intermediate_results/setting5"

melt.id.vars <- c("p.tau_c", "tau.c", "size")
melt.measure.vars <- c("sg.H0.is.reject", "gren.H0.is.reject",
                       "alpha.H0.is.reject", "alpha.tilde.H0.is.reject",
                       "q.n.H0.is.reject")
legend.names <- expression(hat(f)[n]^SG, hat(f)[n]^G,
                           hat(alpha)[n], tilde(alpha)[n], Q[n])
lambda.c.labels <- paste('lambda[C] == ', lambda.c.all, sep = "")
size.labels <- paste('Size == ', size.all, sep = "")

plots.list <- NULL
for (p in p.all) {
  for (lambda in lambda.all) {
    for (p.tau0 in p.tau0.all) {
      size.jumpsize.rej.df <- NULL
      for (lambda.c in lambda.c.all) {
        for (size in size.all) {
          rej.df <- NULL
          res.file.subdir <- sprintf("texp_%s_%s_exp_trunc_%s_p_%s_n_%d",
                                     as.character(lambda), as.character(p.tau0),
                                     as.character(lambda.c),
                                     as.character(p), size)
          rds.files <- list.files(file.path(res.file.path, res.file.subdir),
                                  pattern = "results_grid_no_clip.rds$",
                                  full.names = TRUE)
          if (length(rds.files) == 1L) {
            res <- readRDS(rds.files)
            rej.df.tmp <- res$rej.prop.df
            p.tau_c.tmp <- 1 + pexp(rej.df.tmp$tau.c, rej.df.tmp$lambda)
            tau0.tmp <- qexp(rej.df.tmp$p.tau0, rej.df.tmp$lambda)
            p.tau_c.tmp[rej.df.tmp$tau.c < tau0.tmp] <- ptexp(
              rej.df.tmp[rej.df.tmp$tau.c < tau0.tmp, ]$tau.c,
              rej.df.tmp[rej.df.tmp$tau.c < tau0.tmp, ]$lambda,
              tau0.tmp[rej.df.tmp$tau.c < tau0.tmp])
            rej.df.tmp$p.tau_c <- p.tau_c.tmp

            rej.df <- rbind(rej.df, rej.df.tmp)
            size.jumpsize.rej.df <- rbind(size.jumpsize.rej.df, rej.df.tmp)
          }

        }
      }
      plot.df <- as.data.table(size.jumpsize.rej.df)
      plot.df <- melt(plot.df, id.vars = c(melt.id.vars, "lambda.c"),
                      measure.vars = melt.measure.vars,
```

```r
                      variable.factor = TRUE)
      plot.df$lambda.c <- factor(plot.df$lambda.c, levels = lambda.c.all,
                                 labels = lambda.c.labels)
      plot.df$size <- factor(plot.df$size, levels = size.all, labels = size.labels)

      plot.x.labels <- paste("q", seq(length(unique(plot.df$p.tau_c))), sep = "")
      g1 <- ggplot(plot.df, aes(x = tau.c, y = value, group = variable,
                                color = variable, linetype = variable)) +
        geom_line(linewidth = 1) +
        geom_hline(yintercept = unique(size.jumpsize.rej.df$alpha),
                   colour = 'red', linetype = '3313', linewidth = 1) +
        geom_vline(xintercept = unique(plot.df[plot.df$p.tau_c == 1 - unique(size.jumpsize.rej.df$eps), ][["tau.c"]]),
                   colour = 'black', linetype = '3313', alpha = 0.35, linewidth = 1) +
        scale_x_continuous(breaks = sort(unique(plot.df$tau.c)),
                           labels = plot.x.labels) +
        scale_y_continuous(breaks = seq(0, 1, 0.1), limits = c(0, 1)) +
        theme_bw() +
        theme(legend.position="bottom",
              text = element_text(size = 30),
              axis.text.x = element_text(angle = 90, vjust = 0.5, hjust = 1),
              legend.text.align = 0,
              legend.key.width = unit(0.08, "npc"),
              legend.box.margin = margin(t = -10, r = 25, b = 0, l = 0, unit = "pt")) +
        facet_grid(row = vars(size), cols = vars(lambda.c),
                   labeller = label_parsed) +
        labs(color  = "Method", linetype = "Method") +
        xlab(~ paste(tau["G"])) + ylab("Rejecting proportion") +
        scale_color_hue(labels = legend.names) +
        scale_linetype_discrete(labels = legend.names)


      list.tmp <- list(g1)
      names(list.tmp) <- p
      plots.list <- c(plots.list, list.tmp)
    }
  }
}
```

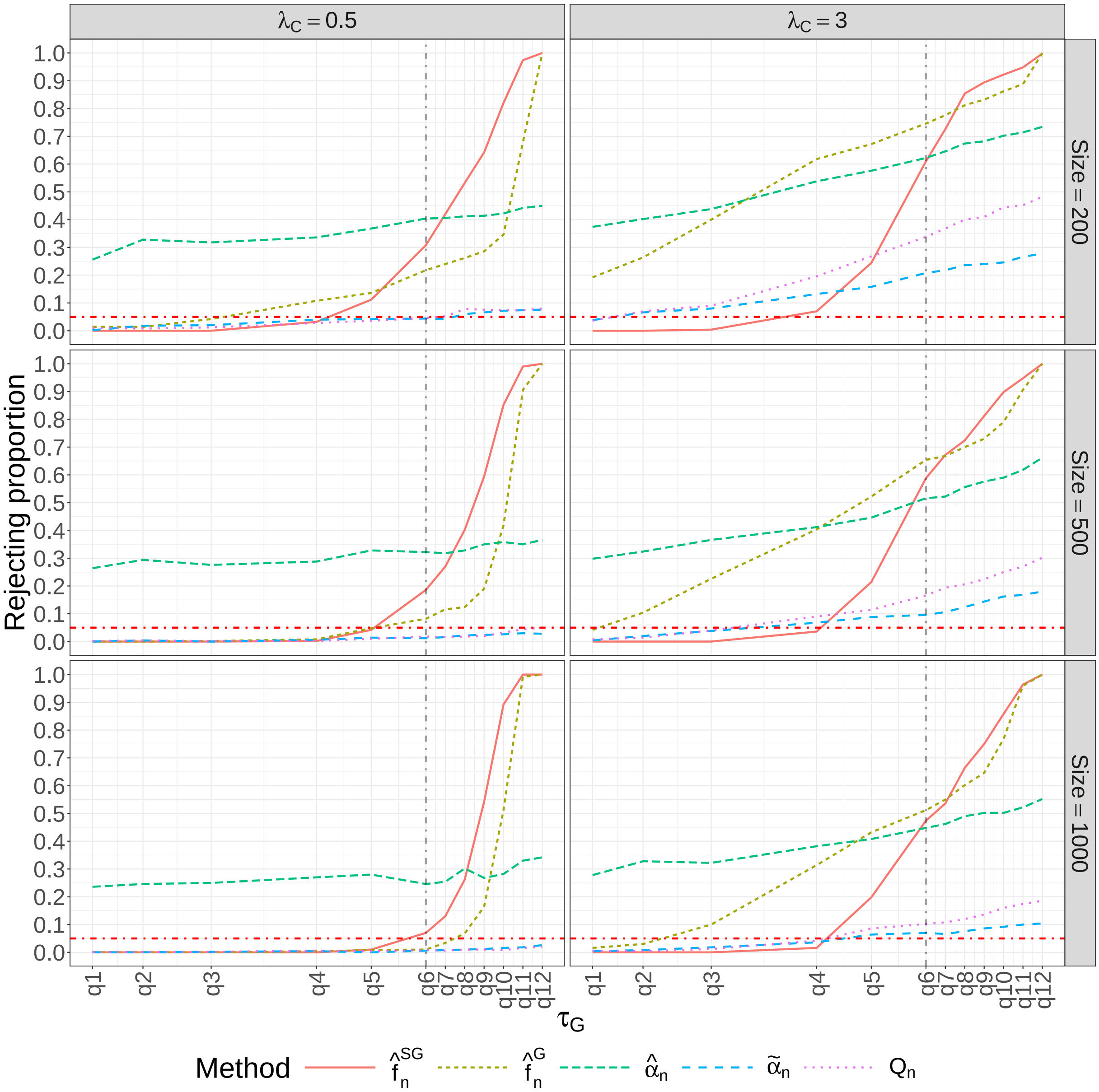

Figure 19: Rejection rate of the null hypothesis of insufficient follow-up for different methods in Setting 5 when p = 0.6 (uncured fraction). (Figure S14 in the Supplementary Material)

Setting 6

This section contains code for generating the table in Section S1.2.6 of the Supplementary Material using the intermediate results.

```
lambda.all <- c(1)
lambda.c.all <- c(0.5)
p.all <- c(0.2, 0.4, 0.8)
size.all <- c(200, 500, 1000)

res.file.path <- "./intermediate_results/setting6"

melt.id.vars <- c("p.tau_c", "tau.c", "size")
melt.measure.vars <- c("gren.H0.is.reject", "sg.H0.is.reject",
                       "alpha.H0.is.reject", "alpha.tilde.H0.is.reject",
                       "q.n.H0.is.reject")
legend.names <- expression(hat(f)[n]^G, hat(f)[n]^SG,
                           hat(alpha)[n], tilde(alpha)[n], Q[n])
lambda.c.labels <- paste('lambda[G] == ', lambda.c.all, sep = "")
size.labels <- paste('Size == ', size.all, sep = "")

y.max.tau.df <- NULL
size.jumpsize.rej.df <- NULL
for (p in p.all) {
  for (lambda in lambda.all) {
    for (lambda.c in lambda.c.all) {
      for (size in size.all) {
        rej.df <- NULL
        res.file.subdir <- sprintf("exp_%s_exp_%s_p_%s_n_%d",
                                   as.character(lambda),
                                   as.character(lambda.c),
                                   as.character(p), size)
        rds.files.tau1 <- list.files(file.path(res.file.path, res.file.subdir),
                                     pattern = "results_grid_no_clip_1.rds$",
                                     full.names = TRUE)
        rds.files.tau2 <- list.files(file.path(res.file.path, res.file.subdir),
                                     pattern = "results_grid_no_clip_2.rds$",
                                     full.names = TRUE)
        t.n.res <- readRDS(file.path(res.file.path, res.file.subdir,
                                     sprintf('p_%g_n_%d.rds', p, size)))
        # t.n.res contains a data.frame with the following information
        # Tn-test considers follow-up as sufficient if
        # (2 * (y.max - y.uncens.max) >= y.max)

        for (rds.files in c(rds.files.tau1, rds.files.tau2)) {
          if (length(rds.files) == 1L) {

            res <- readRDS(rds.files)
            rej.prop.df <- NULL
            tau.c.grid.size <- length(res$res.df)
            rej.df.list <- res$rej.df
            res.df.list <- res$res.df
            for (ii in seq(tau.c.grid.size)) {
              # Tn-test considers follow-up as sufficient if
              # (2 * (y.max - y.uncens.max) >= y.max)
              res.df.list[[ii]]$t.n.H1.is.reject.new <-
                (!t.n.res & res.df.list[[ii]]$t.n.H1.is.reject)
```

```
    rej.df.list[[ii]] <-
      res.df.list[[ii]][, c("rep", "H0.insufficient",
                            "gren.H0.is.reject", "sg.H0.is.reject",
                            "alpha.H0.is.reject", "alpha.tilde.H0.is.reject",
                            "q.n.H0.is.reject", "t.n.H1.is.reject", "t.n.H1.is.reject.new")]
    rej.prop.df.tmp <- cbind(
      res$rej.prop.df[ii, c("lambda", "tau.c", "lambda.c",
                            "p", "size", "eps", "alpha",
                            "tau", "tau.delta")],
      data.frame(t(colMeans(rej.df.list[[ii]][, c(
        "H0.insufficient",
        "gren.H0.is.reject", "sg.H0.is.reject",
        "alpha.H0.is.reject", "alpha.tilde.H0.is.reject",
        "q.n.H0.is.reject", "t.n.H1.is.reject", "t.n.H1.is.reject.new")]))))
    rej.prop.df <- rbind(rej.prop.df, rej.prop.df.tmp)
  }

  has.no.tau.c <- all(!(rej.prop.df$tau.c %in%
                          rej.df[rej.df$lambda.c == lambda.c &
                                   rej.df$p == p &
                                   rej.df$size == size, ]$tau.c))
  has.no.tau <- all(!(rej.prop.df$tau %in%
                        rej.df[rej.df$lambda.c == lambda.c &
                                 rej.df$p == p &
                                 rej.df$size == size, ]$tau))
  if (has.no.tau.c || has.no.tau) {
    rej.df.tmp <- rej.prop.df
    if ("tau.delta" %in% colnames(rej.df.tmp)) {
      rej.df.tmp$tau <- rej.df.tmp$tau.delta
      rej.df.tmp$tau.delta <- NULL
    }
    rej.df.tmp$p.tau_c <- pexp(rej.df.tmp$tau.c, rej.df.tmp$lambda)
    rej.df <- rbind(rej.df, rej.df.tmp)
  }

  has.no.tau.c <- all(!(rej.prop.df$tau.c %in%
                          size.jumpsize.rej.df[
                            size.jumpsize.rej.df$lambda.c == lambda.c &
                              size.jumpsize.rej.df$p == p &
                              size.jumpsize.rej.df$size == size, ]$tau.c))
  has.no.tau <- all(!(rej.prop.df$tau %in%
                        size.jumpsize.rej.df[
                          size.jumpsize.rej.df$lambda.c == lambda.c &
                            size.jumpsize.rej.df$p == p &
                            size.jumpsize.rej.df$size == size, ]$tau))
  if (has.no.tau.c || has.no.tau) {
    rej.df.tmp <- rej.prop.df
    if ("tau.delta" %in% colnames(rej.df.tmp)) {
      rej.df.tmp$tau <- rej.df.tmp$tau.delta
      rej.df.tmp$tau.delta <- NULL
    }
    rej.df.tmp$p.tau_c <- pexp(rej.df.tmp$tau.c, rej.df.tmp$lambda)
    size.jumpsize.rej.df <- rbind(size.jumpsize.rej.df, rej.df.tmp)
  }

  df.tmp <- res$res.df[[length(res$res.df)]][
```

```r
            , c("rep", "y.max", "tau.star")]
          y.max.tau.df.tmp <- data.frame(
            "p" = rep(p, nrow(df.tmp)),
            "lambda" = rep(lambda, nrow(df.tmp)),
            "lambda.c" = rep(lambda.c, nrow(df.tmp)),
            "size" = rep(size, nrow(df.tmp)),
            "rep" = df.tmp$rep,
            "tau" = df.tmp$tau.star,
            "tau.delta" = rep(unique(rej.prop.df$tau.delta), nrow(df.tmp)),
            "y.max" = df.tmp$y.max)
          y.max.tau.df <- rbind(y.max.tau.df, y.max.tau.df.tmp)
        }
      }
    }
  }
 }
}
rej.df.all <- size.jumpsize.rej.df[
  size.jumpsize.rej.df$tau.c == max(size.jumpsize.rej.df$tau.c), ]
rej.df.all$p <- 1 - rej.df.all$p
rej.df.all <- rej.df.all[, c("p", "size", "tau",
                             "gren.H0.is.reject", "sg.H0.is.reject",
                             "alpha.H0.is.reject", "alpha.tilde.H0.is.reject",
                             "q.n.H0.is.reject", "t.n.H1.is.reject.new")]
setorderv(rej.df.all, c("p", "size", "tau"))
rej.df.all$size <- as.character(rej.df.all$size)
rej.df.all$p <- format(rej.df.all$p, nsmall = 1)
```

p	size	δ	$\hat{f}^{G}_{nh}$	$\hat{f}^{SG}_{nh}$	α_n	$\tilde{\alpha}_n$	Q_n	T_n
0.2	200	1.000	1.000	1.000	0.998	0.814	0.996	0.000
0.2	200	2.000	1.000	1.000	0.998	0.814	0.996	0.000
0.2	500	1.000	1.000	1.000	0.994	0.854	0.994	0.002
0.2	500	2.000	1.000	1.000	0.994	0.854	0.994	0.002
0.2	1000	1.000	1.000	1.000	1.000	0.898	1.000	0.002
0.2	1000	2.000	1.000	1.000	1.000	0.898	1.000	0.002
0.6	200	1.000	0.992	0.988	0.976	0.734	0.956	0.000
0.6	200	2.000	0.992	0.984	0.976	0.734	0.956	0.000
0.6	500	1.000	1.000	1.000	0.992	0.790	0.980	0.000
0.6	500	2.000	1.000	0.998	0.992	0.790	0.980	0.000
0.6	1000	1.000	1.000	1.000	0.994	0.876	0.990	0.000
0.6	1000	2.000	1.000	1.000	0.994	0.876	0.990	0.000
0.8	200	1.000	0.974	0.942	0.948	0.624	0.876	0.008
0.8	200	2.000	0.974	0.928	0.948	0.624	0.876	0.008
0.8	500	1.000	0.996	0.994	0.970	0.728	0.940	0.014
0.8	500	2.000	0.996	0.986	0.970	0.728	0.940	0.014
0.8	1000	1.000	0.998	0.998	0.980	0.810	0.966	0.008
0.8	1000	2.000	0.998	0.998	0.980	0.810	0.966	0.008

Table 1: Simulation result (in terms of rejection rate of the null hypotheses) for Setting 6. τ is set to $y_{(n)} + \delta$ for the tests based on $\hat{f}^{G}_{nh}$ and $\hat{f}^{SG}_{nh}$. (Table S7)

Setting 7

This section contains code for generating the table in Section S1.2.7 of the Supplementary Material using the intermediate results.

```
res.file.path <- "./intermediate_results/setting7"
res.df <- readRDS(file.path(res.file.path, "mixlnorm_unif.rds"))

rej.df <- res.df[, c("rep", "H0.insufficient",
                     "gren.H0.is.reject", "sg.H0.is.reject",
                     "alpha.H0.is.reject", "alpha.tilde.H0.is.reject",
                     "q.n.H0.is.reject")]
rej.prop.df <- colMeans(rej.df[, c("gren.H0.is.reject", "sg.H0.is.reject",
                                   "alpha.H0.is.reject", "alpha.tilde.H0.is.reject",
                                   "q.n.H0.is.reject")])
rej.prop.df <- as.data.frame(rej.prop.df)
```

$\hat{f}_{nh}^{G}$	0.390
$\hat{f}_{nh}^{SG}$	0.008
α_n	0.426
$\tilde{\alpha}_n$	0.056
Q_n	0.038

Table 2: Rejection rate of insufficient follow-up for different methods in Setting 7. (Table S8)
