# Supplementary material for: Testing for Sufficient Follow‐Up in Survival Data With a Cure Fraction
Source: Biom J. 2026 Mar 4;68(2):e70121. doi: 10.1002/bimj.70121 (PMC12961184; doi:10.1002/bimj.70121)
Supplement: Supplementary file 1 — Supporting File 1: bimj70121‐sup‐0001‐DataCode.zip. [file BIMJ-68-e70121-s001.zip › code and data/applications/real_data_application.pdf]

Testing for sufficient follow-up in survival data with a cure fraction

Real data application

Yuen, T. P. and Musta E.

2025-11-25

Breast Cancer Study I

This section contains code for generating the results shown in Section 5.1 of the main manuscript. The data is obtained using the “curatedBreastData” package under Bioconductor, see the R code portion below.

Read dataset

```
if (!require("BiocManager", quietly = TRUE))
  install.packages("BiocManager")
BiocManager::install("curatedBreastData")
library(curatedBreastData)

data(curatedBreastDataExprSetList)
dt <- curatedBreastDataExprSetList$study_2034_GPL96_all
df <- data.frame(Y = dt$RFS_months_or_MIN_months_of_RFS,
                 status = 1 - dt$RFS)
df <- as.data.table(df)
```

Kaplan–Meier estimate (Figure 5 in the main manuscript)

```
km.fit <- survfit(Surv(df$Y, df$status) ~ 1)
time.grid <- km.fit$time
surv.all <- km.fit$surv
km.plot.df <- data.frame("time" = time.grid, "surv" = surv.all)

censored.plot.df <- unique(df[df$status == 0, c("Y", "status")])
```

```r
setnames(censored.plot.df, c("Y", "status"), c("time", "event"))
censored.plot.df$event <- 1L
censored.plot.df$plateau <- 0L
censored.plot.df[censored.plot.df$time > max(
  censored.plot.df[censored.plot.df$event == 1, ]$time), ]$plateau <- 1L
censored.plot.df <- merge(km.plot.df, censored.plot.df,
                          by.x = "time", all.x = TRUE)
censored.plot.df$surv <- censored.plot.df$surv - 0.5 *
  diff(c(1,censored.plot.df$surv))
censored.plot.df <- censored.plot.df[!is.na(censored.plot.df$event), ]

# LCM fit
lcm.fit <- gcmlcm(c(0, km.fit$time), c(0, 1 - km.fit$surv), type = "lcm")
lcm.df <- data.frame("x.knots" = lcm.fit$x.knots,
                     "y.knots" = lcm.fit$y.knots)
lcm.df[1:2, ]$y.knots <- NA
g.lcm <- ggplot() +
  geom_step(mapping = aes(x = time, y = 1 - surv),
            data = km.plot.df, color = "blue", linewidth = 1.05) +
  geom_point(aes(x = time, y = 1 - surv),
             data = censored.plot.df, shape = "+",
             color = "blue", size = 4.5, alpha = 0.85) +
  geom_path(mapping = aes(x = x.knots, y = y.knots), data = lcm.df,
            linewidth = 1.05, color = "red", linetype = "dashed") +
  ylim(0, 0.5) + theme_bw() + theme(text = element_text(size = 25)) +
  xlim(0, max(df$Y)) +
  xlab("Time to event (months)") + ylab("Cumulative probability")
plot(g.lcm)
```

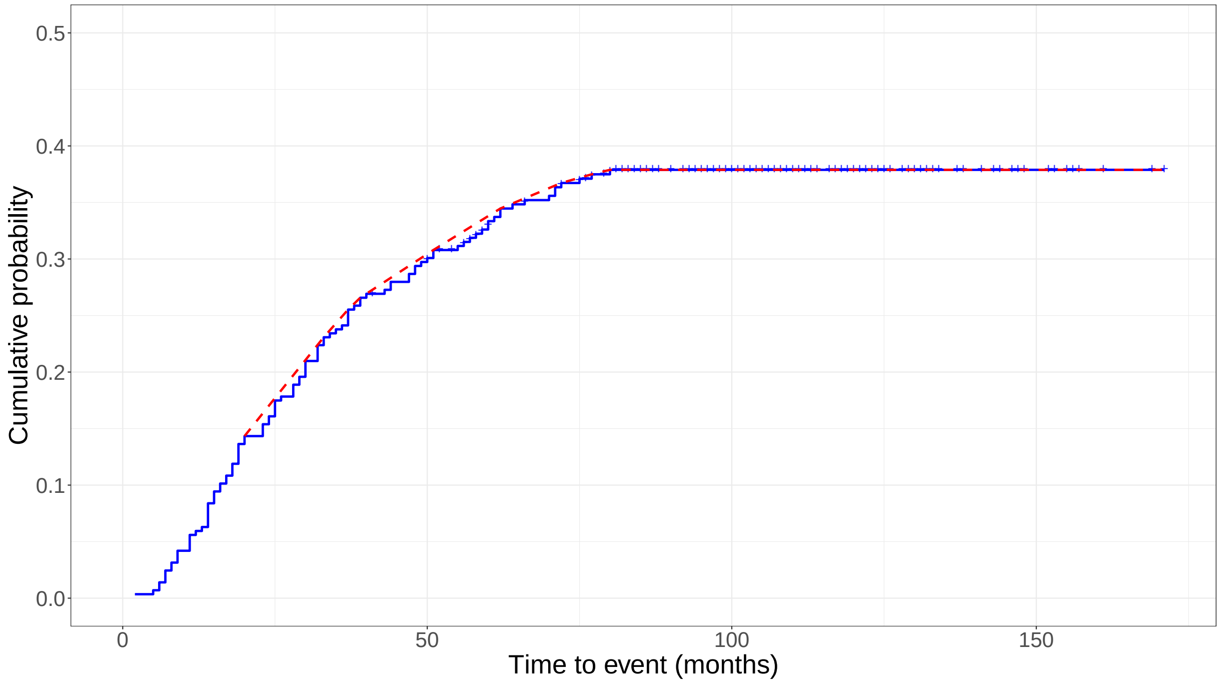

Figure 1: KME (solid) and its least concave majorant (dotted) for breast cancer study I data (Figure 5 in the main manuscript)

Test procedure parameters:

```
a <- 0.34
n.boot <- 1000L
alpha <- 0.05

time.cutoff <- c(90, 110, 130, 150, 171)
eps.all <- c(0.01, 0.025)
tau.all <- c(240, 360)
```

R code for running test procedures

```
res.g.sg.df <- data.table::CJ(time.cutoff, eps.all, tau.all)
colnames(res.g.sg.df) <- c("cutoff", "eps", "tau")
res.g.sg.df$sg.tau.c <- rep(NA_real_, nrow(res.g.sg.df))
res.g.sg.df$sg.test.crit.val <- rep(NA_real_, nrow(res.g.sg.df))
res.g.sg.df$sg.test.p.val <- rep(NA_real_, nrow(res.g.sg.df))
res.g.sg.df$H0.is.rej.sg <- rep(NA, nrow(res.g.sg.df))
res.g.sg.df$g.tau.c <- rep(NA_real_, nrow(res.g.sg.df))
res.g.sg.df$g.test.crit.val <- rep(NA_real_, nrow(res.g.sg.df))
res.g.sg.df$g.test.p.val <- rep(NA_real_, nrow(res.g.sg.df))
```

```r
res.g.sg.df$H0.is.rej.g <- rep(NA, nrow(res.g.sg.df))

res.method.other.df <- data.frame(
  "cutoff" = sort(time.cutoff),
  "alpha.n" = rep(NA, length(time.cutoff)),
  "H0.is.rej.alpha" = rep(NA, length(time.cutoff)),
  "alpha.tilde.n" = rep(NA, length(time.cutoff)),
  "H0.is.rej.alpha.tilde" = rep(NA, length(time.cutoff)),
  "q.n" = rep(NA, length(time.cutoff)),
  "q.n.test.p.val" = rep(NA, length(time.cutoff)),
  "q.n.test.crit.val" = rep(NA, length(time.cutoff)),
  "H0.is.rej.q.n" = rep(NA, length(time.cutoff)),
  "t.n" = rep(NA, length(time.cutoff)),
  "t.n.test.p.val" = rep(NA, length(time.cutoff)),
  "t.n.test.crit.val" = rep(NA, length(time.cutoff)),
  "H.suff.is.rej.t.n" = rep(NA, length(time.cutoff))
)

for (tt in time.cutoff) {
  data.temp <- copy(df)
  data.temp[data.temp$Y > tt, ]$status <- 0
  data.temp[data.temp$Y > tt, ]$Y <- tt

  for (eps in eps.all) {
    for (tau in tau.all) {
      i <- which(res.g.sg.df$cutoff == tt & res.g.sg.df$eps == eps &
                   res.g.sg.df$tau == tau)
      set.seed(7L)
      # Testing insuff. vs suff
      ## Smoothed Grenander
      sg.test.res <- cureSFUTest::sfu.test(
        data.temp$Y, data.temp$status, tau = tau, tau.g = max(data.temp$Y),
        eps = eps, method = "sg", alpha = alpha, n.boot = n.boot)
      res.g.sg.df[i, "sg.tau.c"] <- sg.test.stat <- sg.test.res$statistic
      res.g.sg.df[i, "sg.test.crit.val"] <- sg.test.crit.val <-
        unname(sg.test.res$crit.val)
      res.g.sg.df[i, "sg.test.p.val"] <- sg.test.p.val <-
        unname(sg.test.res$p.value)
      res.g.sg.df[i, "H0.is.rej.sg"] <- H0.is.rej.sg <-
        (sg.test.stat <= sg.test.crit.val)
```

```r
    ## Grenander
    g.test.res <- cureSFUTest::sfu.test(
      data.temp$Y, data.temp$status, tau = tau, tau.g = max(data.temp$Y),
      eps = eps, method = "g", alpha = alpha)
    res.g.sg.df[i, "g.tau.c"] <- g.test.stat <-
      unname(g.test.res$statistic)
    res.g.sg.df[i, "g.test.crit.val"] <- g.test.crit.val <-
      unname(g.test.res$crit.val)
    res.g.sg.df[i, "g.test.p.val"] <- g.test.p.val <-
      unname(g.test.res$p.value)
    res.g.sg.df[i, "H0.is.rej.g"] <- H0.is.rej.g <-
      g.test.stat <= g.test.crit.val
  }
}

i <- which(res.method.other.df$cutoff == tt)
## alpha-type test
### alpha test
res.method.other.df[i, "alpha.n"] <- alpha.res <-
  alpha.test(data.temp$Y, data.temp$status)
res.method.other.df[i, "H0.is.rej.alpha"] <- H0.is.rej.alpha <-
  alpha.res < alpha

### alpha tilde test
res.method.other.df[i, "alpha.tilde.n"] <- alpha.tilde.res <-
  alpha.tilde.test(data.temp$Y, data.temp$status)
res.method.other.df[i, "H0.is.rej.alpha.tilde"] <- H0.is.rej.alpha.tilde <-
  alpha.tilde.res < alpha

### Qn test
res.method.other.df[i, "q.n"] <- q.n.res <-
  q.n.test(data.temp$Y, data.temp$status)
res.method.other.df[i, "q.n.test.crit.val"] <-
  q.n.crit.val(alpha)
res.method.other.df[i, "q.n.test.p.val"] <-
  q.n.p.val(q.n.res)
res.method.other.df[i, "H0.is.rej.q.n"] <- H0.is.rej.q.n <-
  q.n.res > res.method.other.df[i, "q.n.test.crit.val"]
```

```r
  # Testing suff vs insuff.
  if (tt < max(time.cutoff)) {
    t.n.res <- t.n.test(data.temp$Y, data.temp$status, n.boot = n.boot)
    res.method.other.df[i, "t.n"] <- t.n.res$t.n
    res.method.other.df[i, "t.n.test.crit.val"] <- t.n.crit.val(t.n.res, alpha)
    res.method.other.df[i, "t.n.test.p.val"] <- t.n.p.val(t.n.res)
    res.method.other.df[i, "H.suff.is.rej.t.n"] <- H.suff.is.rej.t.n <-
      t.n.res$t.n > res.method.other.df[i, "t.n.test.crit.val"]
  }
}
```

Test result (Table 1):

- $\tilde{H}_0 : q_{1-\epsilon} \geq \tau_G$
- $H_0 : \tau_{F_u} \geq \tau_G$
- $\check{H}_0 : \tau_{F_u} \leq \tau_G$

	$\epsilon = 0.01$, $\tau = 240$		$\epsilon = 0.01$, $\tau = 360$		$\epsilon = 0.025$, $\tau = 240$		$\epsilon = 0.025$, $\tau = 360$					
cutoff	$\hat{f}^{SG}_{nh}$	$\hat{f}^{G}_{nh}$	$\hat{f}^{SG}_{nh}$	$\hat{f}^{G}_{nh}$	$\hat{f}^{SG}_{nh}$	$\hat{f}^{G}_{nh}$	$\hat{f}^{SG}_{nh}$	$\hat{f}^{G}_{nh}$	α_n	$\tilde{\alpha}_n$	Q_n	T_n
90	0.538	1.000	0.542	1.000	0.503	1.000	0.530	1.000	0.002	0.367	0.133	0.082
110	0.588	0.000	0.649	0.000	0.380	0.000	0.542	0.000	0.000	0.049	0.002	0.103
130	0.144	0.000	0.234	0.000	0.035	0.000	0.131	0.000	0.000	0.001	0.000	0.160
150	0.007	0.000	0.015	0.000	0.001	0.000	0.006	0.000	0.000	0.000	0.000	0.307
171	0.000	0.000	0.000	0.000	0.000	0.000	0.000	0.000	0.000	0.000	0.000	

Table 1: p-values of testing $\tilde{H}_0$, H_0 or $\check{H}_0$ at different follow-up cutoffs for breast cancer study I (rounded to three decimal places). (Table 1 in the main manuscript)

Breast Cancer Study II (using a mock dataset)

This section contains code for generating the results shown in Section 5.2 of the main manuscript. We run the analysis on a mock dataset that mimics the original Breast Cancer Study II dataset to demonstrate the replicability of the code. The results may not be identical to the actual Breast Cancer Study II in the main manuscript.

Read dataset

```
df <- fread("./breast_cancer_II_data.csv")
```

Kaplan–Meier estimate (Figure 6 in the main manuscript)

```
km.fit <- survfit(Surv(df$Y, df$status) ~ 1)
time.grid <- km.fit$time
surv.all <- km.fit$surv
km.plot.df <- data.frame("time" = time.grid, "surv" = surv.all)

censored.plot.df <- unique(df[df$status == 0, c("Y", "status")])
setnames(censored.plot.df, c("Y", "status"), c("time", "event"))
censored.plot.df$event <- 1L
censored.plot.df$plateau <- 0L
censored.plot.df[censored.plot.df$time > max(
  censored.plot.df[censored.plot.df$event == 1, ]$time), ]$plateau <- 1L
censored.plot.df <- merge(km.plot.df, censored.plot.df,
                          by.x = "time", all.x = TRUE)
censored.plot.df$surv <- censored.plot.df$surv - 0.5 *
  diff(c(1,censored.plot.df$surv))
censored.plot.df <- censored.plot.df[!is.na(censored.plot.df$event), ]

# LCM fit
lcm.fit <- gcmlcm(c(0, km.fit$time), c(0, 1 - km.fit$surv), type = "lcm")
lcm.df <- data.frame("x.knots" = lcm.fit$x.knots,
                     "y.knots" = lcm.fit$y.knots)
lcm.df[1:2, ]$y.knots <- NA
g.lcm <- ggplot() +
  geom_step(mapping = aes(x = time, y = 1 - surv),
            data = km.plot.df, color = "blue", linewidth = 1.05) +
  geom_point(aes(x = time, y = 1 - surv),
             data = censored.plot.df, shape = "+",
```

```
             color = "blue", size = 4.5, alpha = 0.85) +
  geom_path(mapping = aes(x = x.knots, y = y.knots), data = lcm.df,
            linewidth = 1.05, color = "red", linetype = "dashed") +
  ylim(0, 0.5) + theme_bw() + theme(text = element_text(size = 25)) +
  xlim(0, max(df$Y)) +
  xlab("Time to event (months)") + ylab("Cumulative probability")
plot(g.lcm)
```

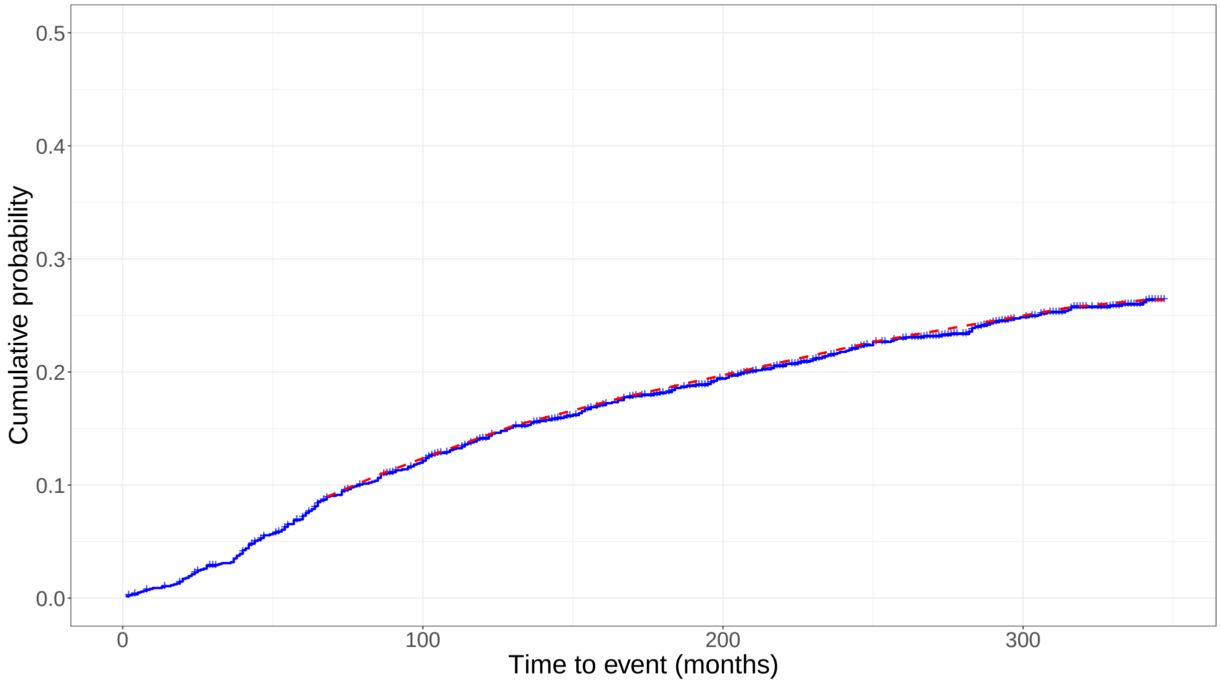

Figure 2: KME (solid) and its least concave majorant (dotted) for breast cancer study II data (Figure 6 in the main manuscript)

Test procedure parameters:

```
a <- 0.34
n.boot <- 1000L
alpha <- 0.05

time.cutoff <- c(seq(180, 340, by = 20), 347)
eps.all <- c(0.01, 0.025)
tau.all <- c(360, 480)
```

R code for running test procedures

```
res.g.sg.df <- data.table::CJ(time.cutoff, eps.all, tau.all)
colnames(res.g.sg.df) <- c("cutoff", "eps", "tau")
res.g.sg.df$sg.tau.c <- rep(NA_real_, nrow(res.g.sg.df))
res.g.sg.df$sg.test.crit.val <- rep(NA_real_, nrow(res.g.sg.df))
res.g.sg.df$sg.test.p.val <- rep(NA_real_, nrow(res.g.sg.df))
res.g.sg.df$H0.is.rej.sg <- rep(NA, nrow(res.g.sg.df))
res.g.sg.df$g.tau.c <- rep(NA_real_, nrow(res.g.sg.df))
res.g.sg.df$g.test.crit.val <- rep(NA_real_, nrow(res.g.sg.df))
res.g.sg.df$g.test.p.val <- rep(NA_real_, nrow(res.g.sg.df))
res.g.sg.df$H0.is.rej.g <- rep(NA, nrow(res.g.sg.df))

res.method.other.df <- data.frame(
  "cutoff" = sort(time.cutoff),
  "alpha.n" = rep(NA, length(time.cutoff)),
  "H0.is.rej.alpha" = rep(NA, length(time.cutoff)),
  "alpha.tilde.n" = rep(NA, length(time.cutoff)),
  "H0.is.rej.alpha.tilde" = rep(NA, length(time.cutoff)),
  "q.n" = rep(NA, length(time.cutoff)),
  "q.n.test.p.val" = rep(NA, length(time.cutoff)),
  "q.n.test.crit.val" = rep(NA, length(time.cutoff)),
  "H0.is.rej.q.n" = rep(NA, length(time.cutoff))
)

for (tt in time.cutoff) {
  data.temp <- copy(df)
  data.temp[data.temp$Y > tt, ]$status <- 0
  data.temp[data.temp$Y > tt, ]$Y <- tt

  for (eps in eps.all) {
    for (tau in tau.all) {
      i <- which(res.g.sg.df$cutoff == tt & res.g.sg.df$eps == eps &
                 res.g.sg.df$tau == tau)
      set.seed(7L)
      # Testing insuff. vs suff
      ## Smoothed Grenander
      sg.test.res <- cureSFUTest::sfu.test(
        data.temp$Y, data.temp$status, tau = tau, tau.g = max(data.temp$Y),
        eps = eps, method = "sg", alpha = alpha, n.boot = n.boot)
```

```r
    res.g.sg.df[i, "sg.tau.c"] <- sg.test.stat <- sg.test.res$statistic
    res.g.sg.df[i, "sg.test.crit.val"] <- sg.test.crit.val <-
      unname(sg.test.res$crit.val)
    res.g.sg.df[i, "sg.test.p.val"] <- sg.test.p.val <-
      unname(sg.test.res$p.value)
    res.g.sg.df[i, "H0.is.rej.sg"] <- H0.is.rej.sg <-
      (sg.test.stat <= sg.test.crit.val)

    ## Grenander
    g.test.res <- cureSFUTest::sfu.test(
      data.temp$Y, data.temp$status, tau = tau, tau.g = max(data.temp$Y),
      eps = eps, method = "g", alpha = alpha)
    res.g.sg.df[i, "g.tau.c"] <- g.test.stat <-
      unname(g.test.res$statistic)
    res.g.sg.df[i, "g.test.crit.val"] <- g.test.crit.val <-
      unname(g.test.res$crit.val)
    res.g.sg.df[i, "g.test.p.val"] <- g.test.p.val <-
      unname(g.test.res$p.value)
    res.g.sg.df[i, "H0.is.rej.g"] <- H0.is.rej.g <-
      g.test.stat <= g.test.crit.val
  }
}

i <- which(res.method.other.df$cutoff == tt)
## alpha-type test
### alpha test
res.method.other.df[i, "alpha.n"] <- alpha.res <-
  alpha.test(data.temp$Y, data.temp$status)
res.method.other.df[i, "H0.is.rej.alpha"] <- H0.is.rej.alpha <-
  alpha.res < alpha

### alpha tilde test
res.method.other.df[i, "alpha.tilde.n"] <- alpha.tilde.res <-
  alpha.tilde.test(data.temp$Y, data.temp$status)
res.method.other.df[i, "H0.is.rej.alpha.tilde"] <- H0.is.rej.alpha.tilde <-
  alpha.tilde.res < alpha

### Qn test
res.method.other.df[i, "q.n"] <- q.n.res <-
  q.n.test(data.temp$Y, data.temp$status)
```

```r
  res.method.other.df[i, "q.n.test.crit.val"] <-
    q.n.crit.val(alpha)
  res.method.other.df[i, "q.n.test.p.val"] <-
    q.n.p.val(q.n.res)
  res.method.other.df[i, "H0.is.rej.q.n"] <- H0.is.rej.q.n <-
    q.n.res > res.method.other.df[i, "q.n.test.crit.val"]
}
```

Test result (Table 2):

- $\tilde{H}_0 : q_{1-\epsilon} \geq \tau_G$
- $H_0 : \tau_{F_u} \geq \tau_G$

	$\epsilon = 0.01$ $\tau = 240$		$\epsilon = 0.01$ $\tau = 360$		$\epsilon = 0.025$ $\tau = 240$		$\epsilon = 0.025$ $\tau = 360$				
cutoff	$\hat{f}_{nh}^{SG}$	$\hat{f}_{nh}^{G}$	$\hat{f}_{nh}^{SG}$	$\hat{f}_{nh}^{G}$	$\hat{f}_{nh}^{SG}$	$\hat{f}_{nh}^{G}$	$\hat{f}_{nh}^{SG}$	$\hat{f}_{nh}^{G}$	α_n	$\tilde{\alpha}_n$	Q_n
180	0.991	1.000	0.992	1.000	0.988	1.000	0.990	1.000	0.368	0.368	0.750
200	1.000	1.000	1.000	1.000	1.000	1.000	1.000	1.000	0.050	0.135	0.316
220	1.000	1.000	1.000	1.000	1.000	1.000	1.000	1.000	0.050	0.135	0.562
240	1.000	1.000	1.000	1.000	1.000	1.000	1.000	1.000	0.368	0.368	0.562
260	1.000	1.000	1.000	1.000	1.000	1.000	1.000	1.000	0.135	0.368	0.422
280	0.881	1.000	0.906	1.000	0.788	1.000	0.881	1.000	0.135	0.368	0.562
300	1.000	1.000	1.000	1.000	1.000	1.000	1.000	1.000	0.368	0.368	0.750
320	1.000	1.000	1.000	1.000	0.998	1.000	1.000	1.000	0.018	0.135	0.422
340	0.875	1.000	0.983	1.000	0.305	1.000	0.970	1.000	1.000	0.368	0.750
347	0.739	1.000	0.947	1.000	0.007	0.000	0.931	1.000	0.135	0.368	0.562

Table 2: p-values of testing $\tilde{H}_0$ or H_0 at different follow-up cutoffs for the breast cancer study II (rounded to three decimal places). (Table 2 in the main manuscript)

Session info

```r
sessionInfo()
```

```
## R version 4.3.2 (2023-10-31)
## Platform: aarch64-apple-darwin20 (64-bit)
## Running under: macOS Sonoma 14.7.5
##
## Matrix products: default
## BLAS:   /Library/Frameworks/R.framework/Versions/4.3-arm64/Resources/lib/libRblas.0.dylib
## LAPACK: /Library/Frameworks/R.framework/Versions/4.3-arm64/Resources/lib/libRlapack.dylib;
##
## locale:
## [1] en_US.UTF-8/en_US.UTF-8/en_US.UTF-8/C/en_US.UTF-8/en_US.UTF-8
##
## time zone: Europe/Amsterdam
```

```
## tzcode source: internal
##
## attached base packages:
## [1] stats     graphics  grDevices utils     datasets  methods   base
##
## other attached packages:
##  [1] curatedBreastData_2.30.0 BiocStyle_2.30.0         Biobase_2.62.0
##  [4] BiocGenerics_0.48.1      impute_1.76.0            XML_3.99-0.16.1
##  [7] BiocManager_1.30.22      cureSFUTest_2.0.0        fdrtool_1.2.17
## [10] survival_3.5-7           ggplot2_3.5.2            data.table_1.14.10
## [13] xtable_1.8-4             devtools_2.4.5           usethis_2.2.3
## [16] knitr_1.45
##
## loaded via a namespace (and not attached):
##  [1] utf8_1.2.4        generics_0.1.3    lattice_0.21-9    digest_0.6.34
##  [5] magrittr_2.0.3    evaluate_0.23     grid_4.3.2        pkgload_1.3.4
##  [9] fastmap_1.1.1     Matrix_1.6-1.1    pkgbuild_1.4.3    sessioninfo_1.2.3
## [13] urlchecker_1.0.1  promises_1.3.0    purrr_1.0.2       fansi_1.0.6
## [17] scales_1.3.0      cli_3.6.2         shiny_1.8.1.1     rlang_1.1.3
## [21] splines_4.3.2     ellipsis_0.3.2    munsell_0.5.0     remotes_2.5.0
## [25] withr_3.0.0       cachem_1.0.8      yaml_2.3.8        tools_4.3.2
## [29] memoise_2.0.1     dplyr_1.1.4       colorspace_2.1-0  httpuv_1.6.15
## [33] vctrs_0.6.5       R6_2.5.1          mime_0.12         lifecycle_1.0.4
## [37] fs_1.6.3          htmlwidgets_1.6.4 miniUI_0.1.1.1    pkgconfig_2.0.3
## [41] pillar_1.9.0      later_1.3.2       gtable_0.3.4      glue_1.7.0
## [45] profvis_0.4.0     Rcpp_1.0.12       xfun_0.42         tibble_3.2.1
## [49] tidyselect_1.2.0  rstudioapi_0.15.0 farver_2.1.1      htmltools_0.5.7
## [53] labeling_0.4.3    rmarkdown_2.29    compiler_4.3.2
```
