# Supplementary material for: Testing for Sufficient Follow‐Up in Survival Data With a Cure Fraction
Source: Biom J. 2026 Mar 4;68(2):e70121. doi: 10.1002/bimj.70121 (PMC12961184; doi:10.1002/bimj.70121)
Supplement: Supplementary file 2 — Supporting File 2: bimj70121‐sup‐0002‐SuppMat.pdf. [file BIMJ-68-e70121-s002.pdf]

Supplementary Material:
Testing for sufficient follow-up in survival data with a cure fraction

Tsz Pang Yuen and Eni Musta

S1 Simulation study

S1.1 Simulation settings

Tables S1–S5 report the censoring rates over different values of τ_G for Settings 1–5.

Table S1: Censoring rates for Setting 1.

p	$\Delta G(\tau_G)$	Censoring rate
0.2	0	82.91% – 87.84%
	0.02	82.85% – 87.73%
	0.05	82.76% – 87.56%
	0.2	82.34% – 86.69%
0.6	0	48.74% – 63.52%
	0.02	48.56% – 63.16%
	0.05	48.29% – 62.64%
	0.2	47.02% – 60.04%
0.8	0	31.64% – 51.35%
	0.02	31.39% – 50.87%
	0.05	31.05% – 50.18%
	0.2	29.33% – 46.72%

Table S2: Censoring rates for Setting 2.

p	λ	Censoring rate
0.2	0.4	91.13% – 91.18%
	1	86.69% – 87.12%
	5	81.86% – 83.30%
0.6	0.4	73.33% – 73.49%
	1	60.02% – 61.29%
	5	45.56% – 49.87%
0.8	0.4	64.46% – 64.66%
	1	46.65% – 48.36%
	5	27.34% – 33.09%

Table S3: Censoring rates for Setting 3.

p	$\Delta G(\tau_G)$	Censoring rate
0.2	0	80.83% – 85.08%
	0.02	80.82% – 85.02%
	0.05	80.80% – 84.92%
	0.2	80.68% – 84.46%
0.6	0	42.57% – 55.26%
	0.02	42.53% – 55.08%
	0.05	42.45% – 54.80%
	0.2	42.08% – 53.41%
0.8	0	23.39% – 40.28%
	0.02	23.32% – 40.03%
	0.05	23.22% – 39.66%
	0.2	22.73% – 37.81%

Table S4: Censoring rates for Setting 4.

p	$\Delta G(\tau_G)$	Censoring rate
0.6	0	48.38% – 63.85%
	0.02	48.22% – 63.48%
	0.05	47.96% – 62.94%
	0.2	46.71% – 60.28%

Table S5: Censoring rates for Setting 5.

p	λ_C	Censoring rate
0.6	0.5	45.24% – 49.75%
	3	62.14% – 63.23%

S1.2 Simulation results

q_1, q_2, q_3, q_4, q_6, and q_{12} are the 90%, 92.5%, 95%, 97.5%, 99%, and 99.9% quantiles of F_u, resp.; q_5 is the mid-point between q_4 and q_6; $q_7, \cdots, q_{11}$ are 5 evenly separated points between q_6 and q_{12} in the figures in this section. Table S6 reports their actual values for Settings 1–5.

Table S6: Actual values of q_1, ..., q_{12} for Settings 1–5.

τ_G	Setting				
	1	2	3	4	5
q_1	2.3026	0.4605	7.9528	2.2164	0.4433
q_2	2.5903	0.5181	10.0642	2.4740	0.4948
q_3	2.9957	0.5991	13.4616	2.8218	0.5644
q_4	3.6889	0.7378	20.4117	3.3596	0.6719
q_5	4.1470	0.8294	26.1116	3.6383	0.7277
q_6	4.6052	0.9210	31.8114	3.9170	0.7834
q_7	4.9889	0.9978	38.4388	4.0160	0.8032
q_8	5.3727	1.0745	45.0661	4.1149	0.8230
q_9	5.7565	1.1513	51.6935	4.2139	0.8428
q_{10}	6.1402	1.2280	58.3209	4.3129	0.8626
q_{11}	6.5240	1.3048	64.9483	4.4118	0.8824
q_{12}	6.9078	1.3816	71.5756	4.5108	0.9022

Remark The rejection rate for the α_n test is computed using the decision rule: H_0 is rejected if $\alpha_n < 0.05$ as originally proposed in Maller and Zhou (1992, 1994) as such decision rule is still being widely used in practice. As mentioned in Maller and Zhou (1996, Page 85), it is more appropriate to compare α_n with the 5% quantile of its (limiting) distribution. Therefore the type I error rate of the α_n test reported in this section is inflated. The results for the α_n test here are provided to increase awareness within the community of the risks associated with the use of this decision rule.

S1.2.1 Setting 1

Figures S1–S3 show the rejection rate of insufficient follow-up against τ_G for Setting 1, each figure with different p. To study the effect of the choice of τ on the rejection rate, we considered 3 different τ's, namely $\tau_1 \approx 7.601$ (the 99.95% quantile of F_u), $\tau_2 \approx 9.210$ (the 99.99% quantile of F_u), and $\tau_3 = (\tau_1 + \tau_2)/2 \approx 8.406$. The rejection rate of $\hat{f}_{nh}^{SG}$ using τ_1, τ_2, and τ_3 are rendered by solid, long dashed, and dashed lines, respectively, in Figure S4. In summary, larger τ results in a more conservative procedure, i.e. better control on the empirical level but with some loss of empirical power.

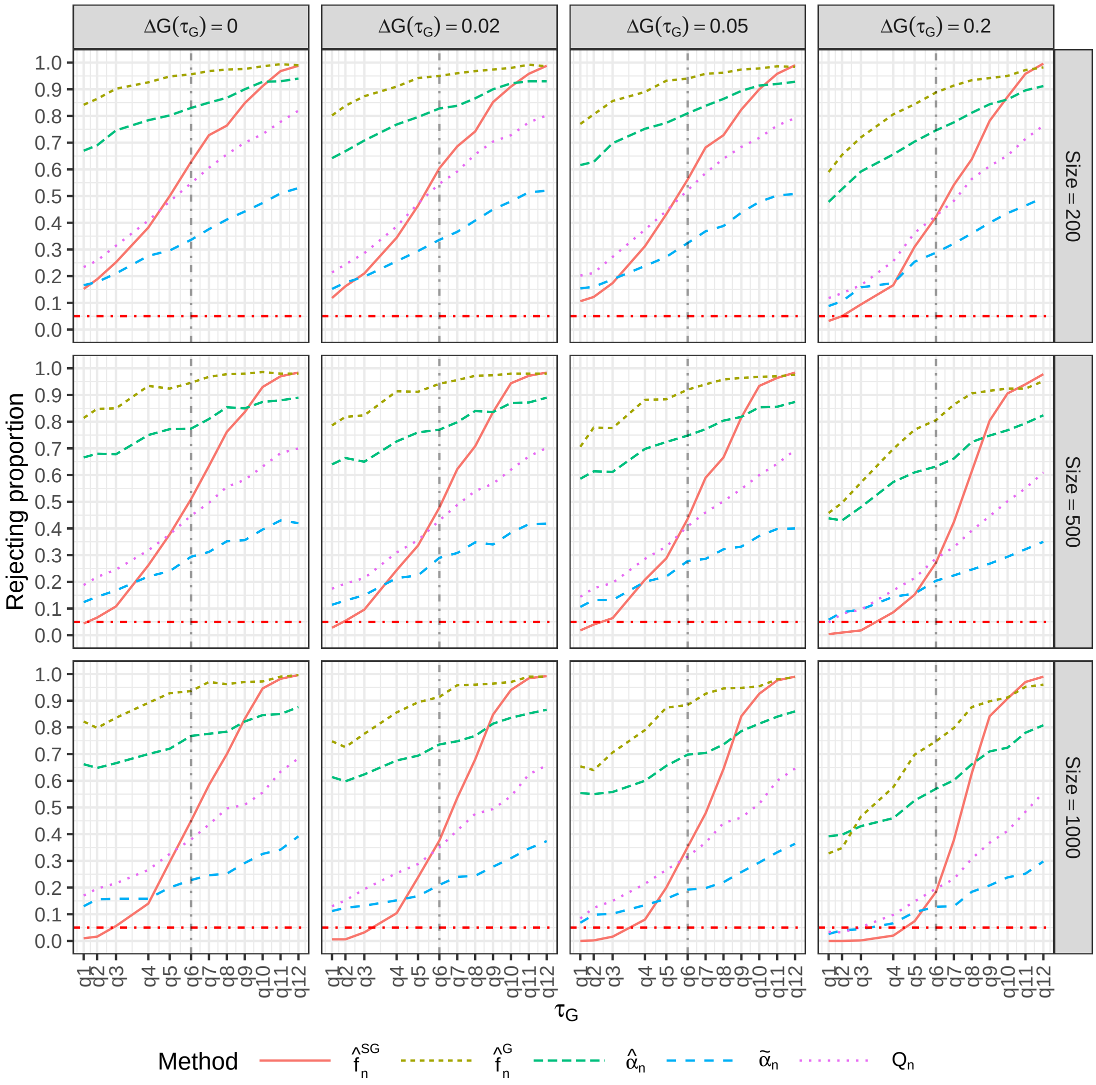

Figure S1: Rejection rate of the null hypothesis of insufficient follow-up for different methods in Setting 1 when $p = 0.2$ (uncured fraction).

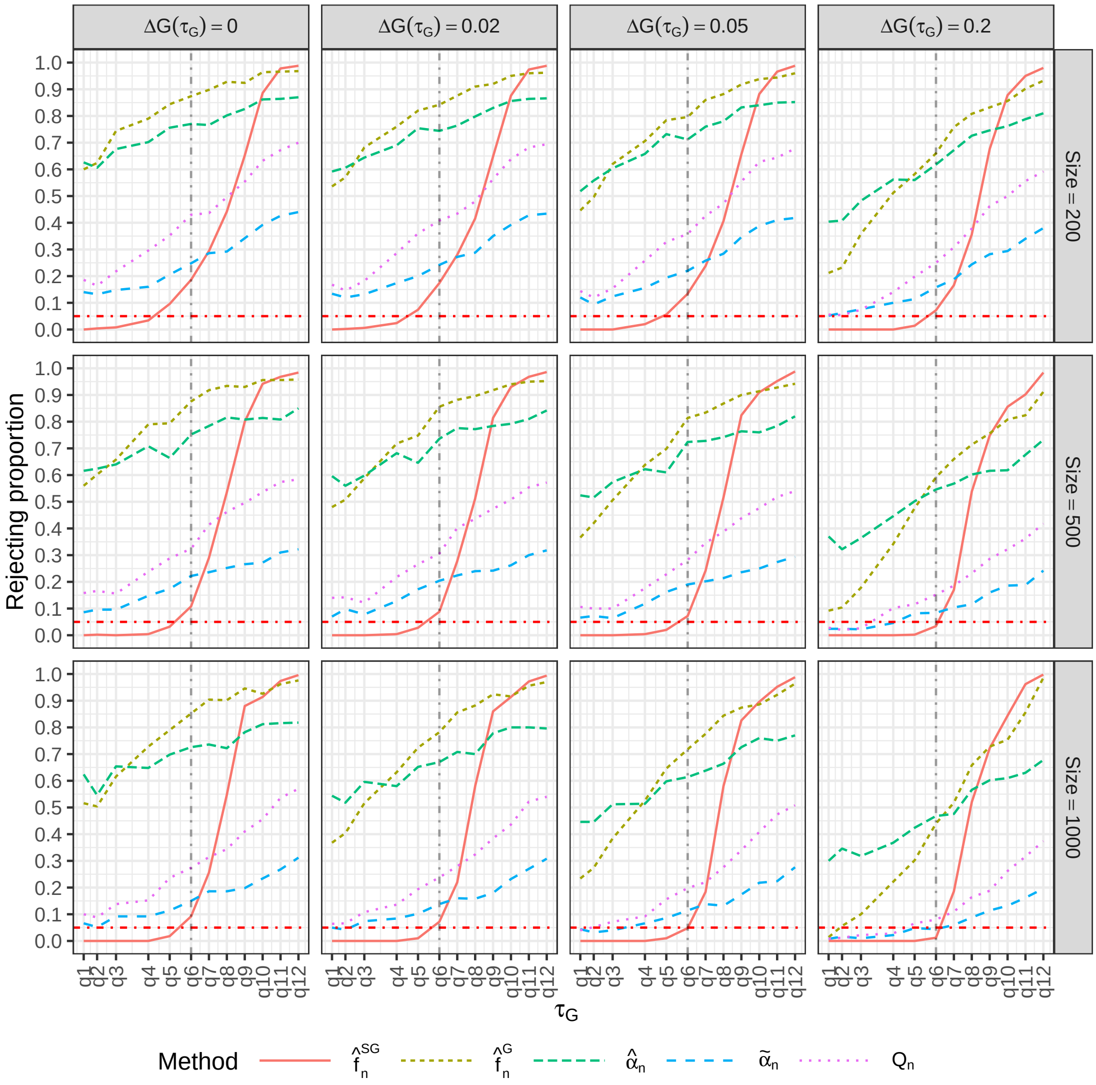

Figure S2: Rejection rate of the null hypothesis of insufficient follow-up for different methods in Setting 1 when $p = 0.6$ (uncured fraction).

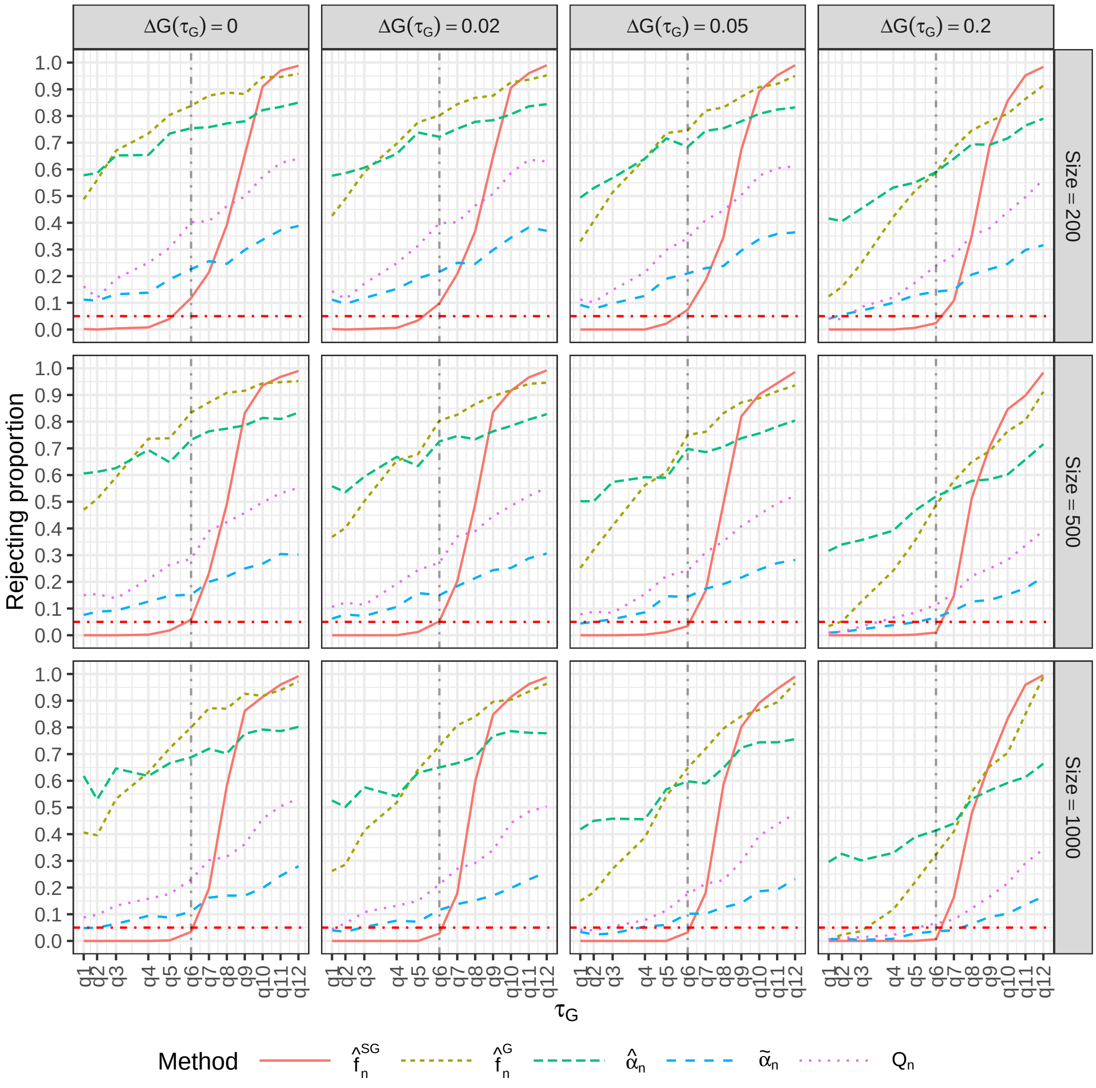

Figure S3: Rejection rate of the null hypothesis of insufficient follow-up for different methods in Setting 1 when $p = 0.8$ (uncured fraction).

Sensitivity of τ

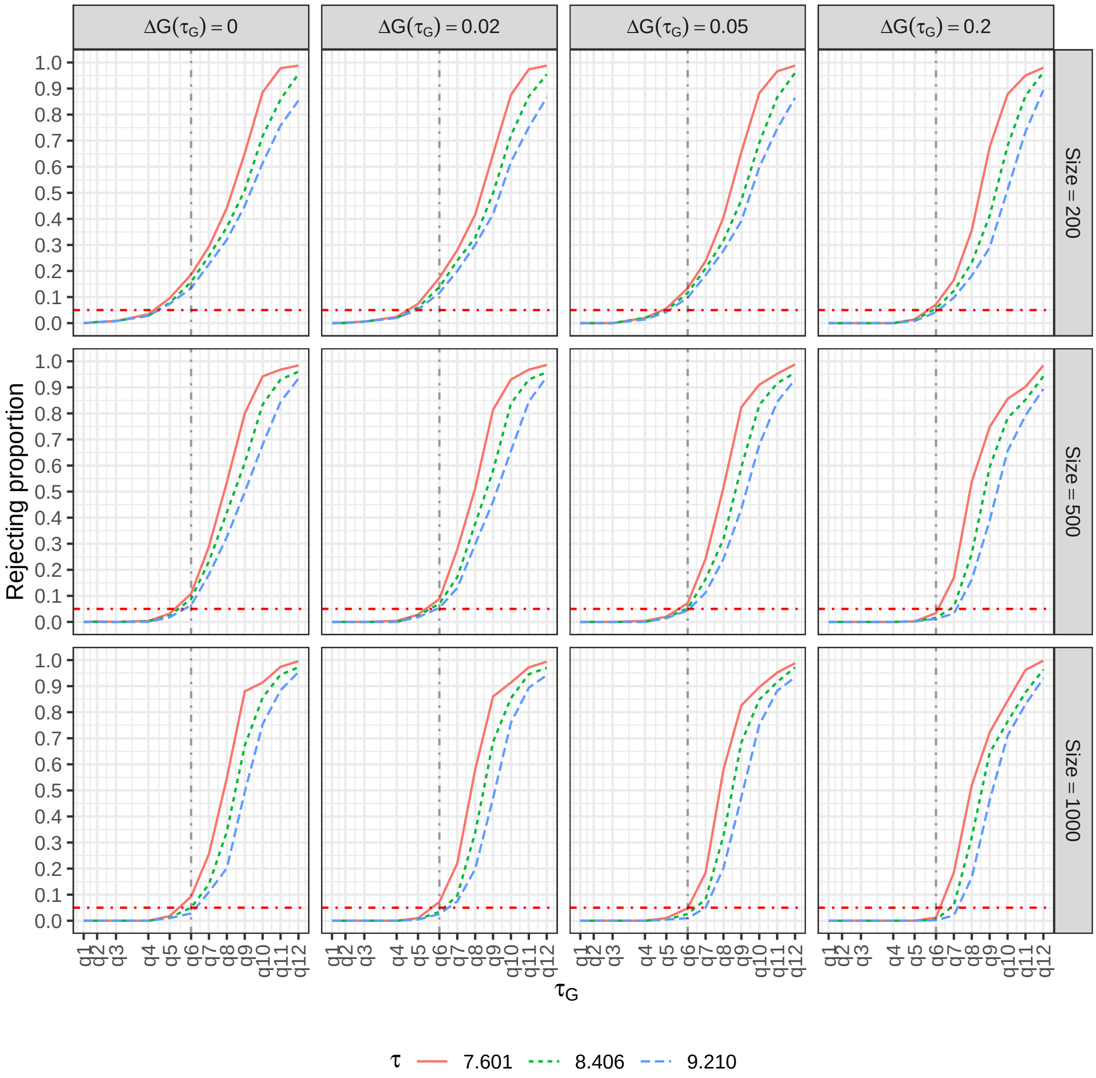

Figure S4: Rejection rate of the null hypothesis of insufficient follow-up for the test based on $\hat{f}_{nh}^{SG}$ with different τ in Setting 1 when $p = 0.6$ (uncured fraction).

Comparison with RECeUS

Figure S5 shows the rejection rate of insufficient follow-up against τ_G for Setting 1, when $n = 500$, $p = 0.6$, and $\Delta G(\tau_G) = 0.02$. The method RECeUS-AIC by Selukar and Othus (2023) is included for comparison in addition to $\hat{f}_{nh}^{SG}$, $\hat{f}_n^G$, Q_n. For the RECeUS-AIC procedure, we use the same thresholds specified in Section 2.1 of Selukar and Othus (2023) (2.5% for $\hat{\pi}_n$ and 5% for $\hat{r}_n$). In summary, $\hat{f}_{nh}^{SG}$ performs better in terms of empirical level, while the RECeUS-AIC procedure demonstrates higher power in the region where τ_G lies between q_6 and q_{10}. When comparing the Q_n test with RECeUS-AIC, the latter controls the level more effectively when τ_G is between q_1 and q_3, but not in the region from q_3 to q_6, although RECeUS-AIC exhibits higher empirical power than the Q_n test.

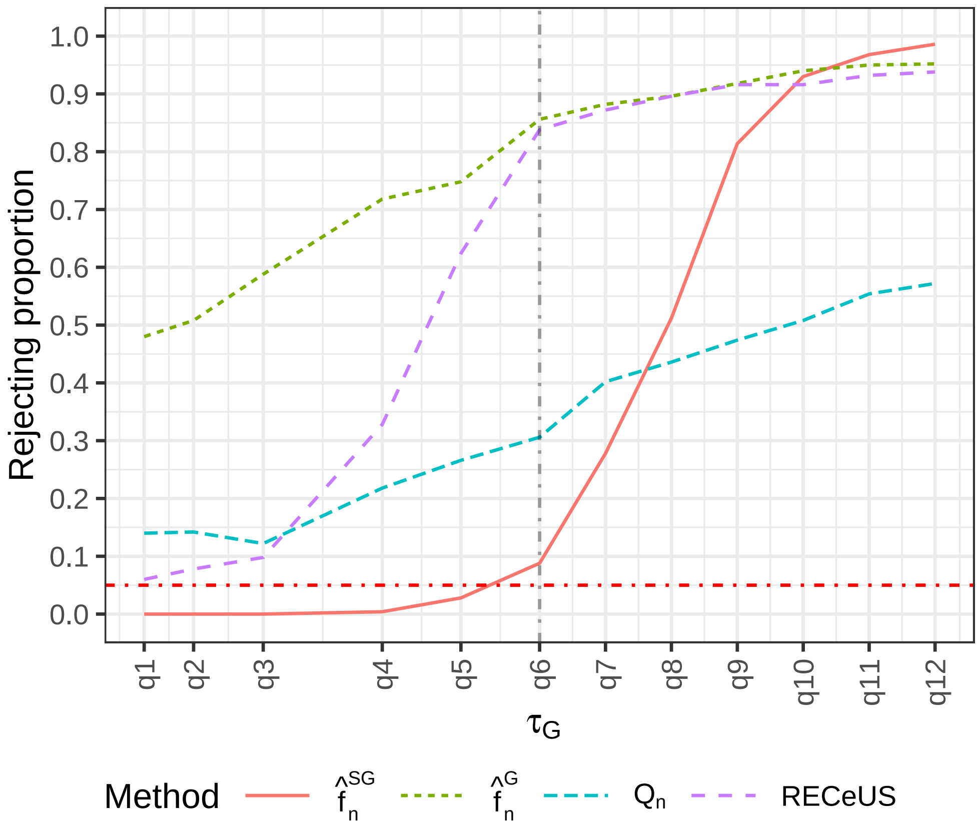

Figure S5: Rejection rate of the null hypothesis of insufficient follow-up for different methods ($\hat{f}_{nh}^{SG}$, $\hat{f}_n^G$, Q_n and RECeUS-AIC) in Setting 1 when $n = 500$, $\Delta G(\tau_G) = 0.02$, and $p = 0.6$ (uncured fraction).

S1.2.2 Setting 2

Figures S6–S8 depict the rejection rate of insufficient follow-up against τ_G for Setting 2, each figure with different p. In Setting 2, the uncured subjects have an exponential distribution with rate $\lambda \in \{0.4, 1, 5\}$, while the censoring time has an exponential distribution with the rate fixed at 0.5. The censoring rate decreases as λ increases, which affects the performance of the testing procedure. In particular, $\lambda = 0.4$ corresponds to a very high censoring rate since the density of the censoring variable decreases quicker than that of the event times and all methods have problems in controlling the level of the test. We observe that each method performs better, in terms of empirical level and power, when λ is larger.

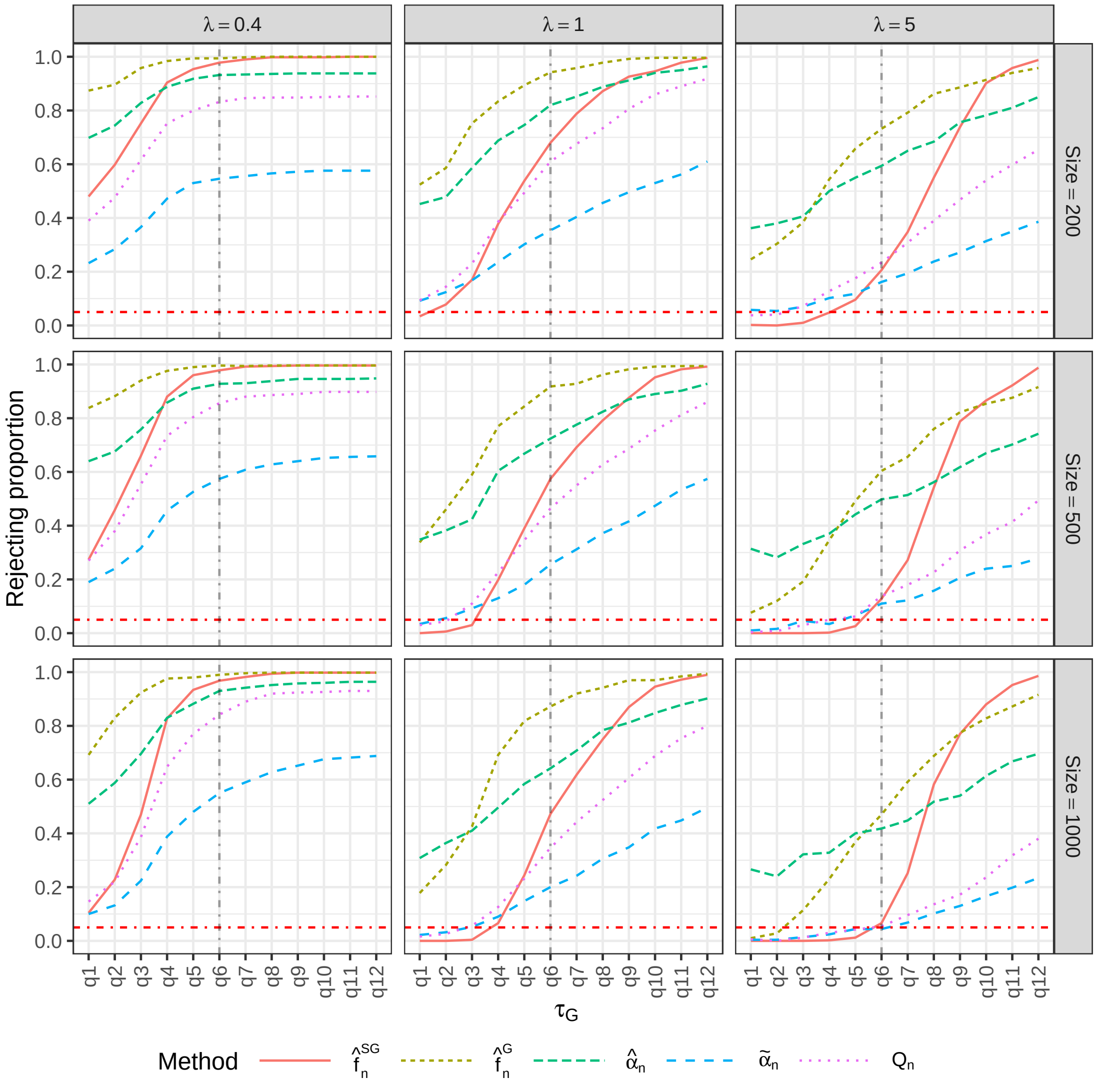

Figure S6: Rejection rate for Setting 2 when $p = 0.2$ (uncured fraction).

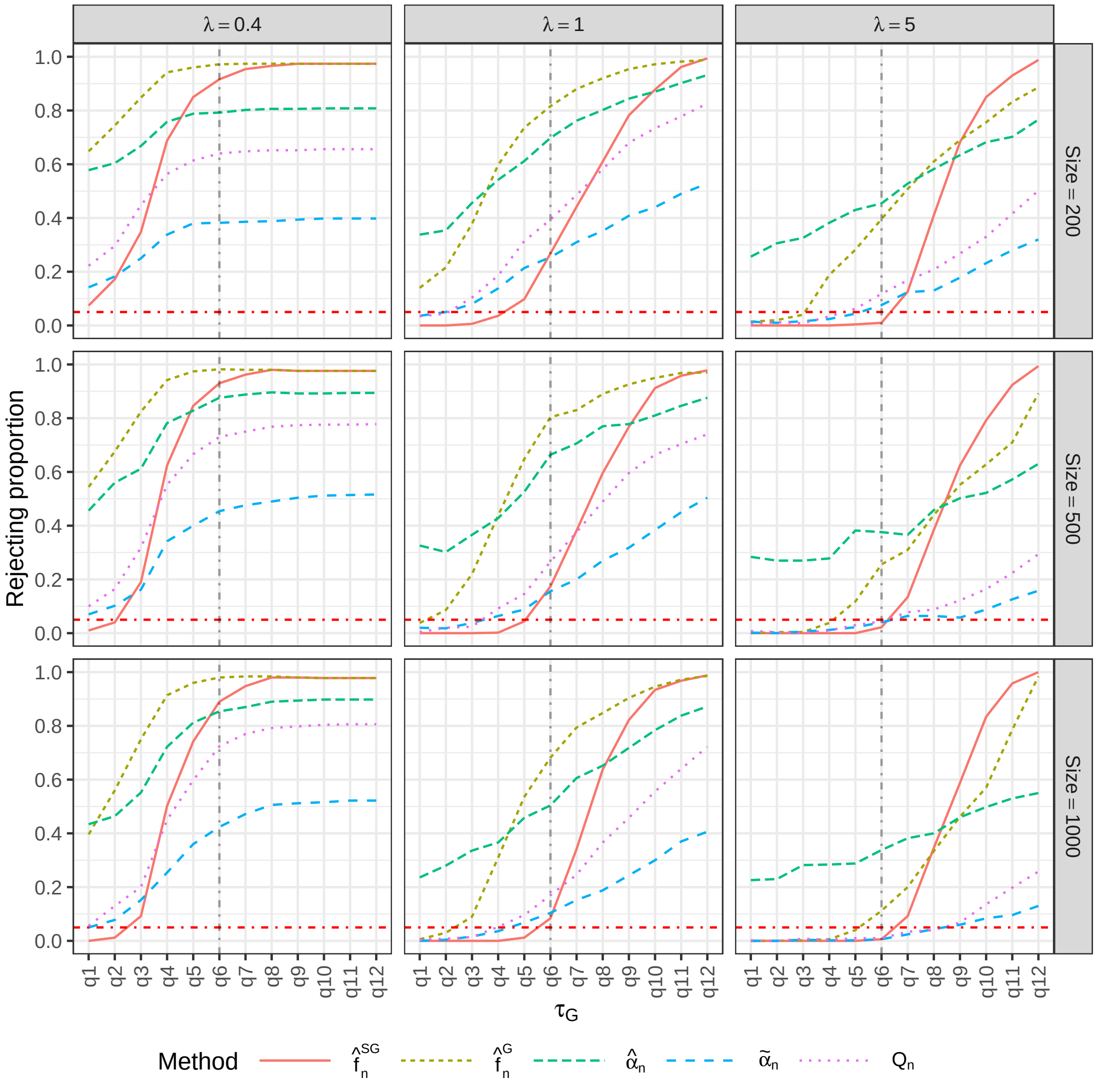

Figure S7: Rejection rate for Setting 2 when $p = 0.6$ (uncured fraction).

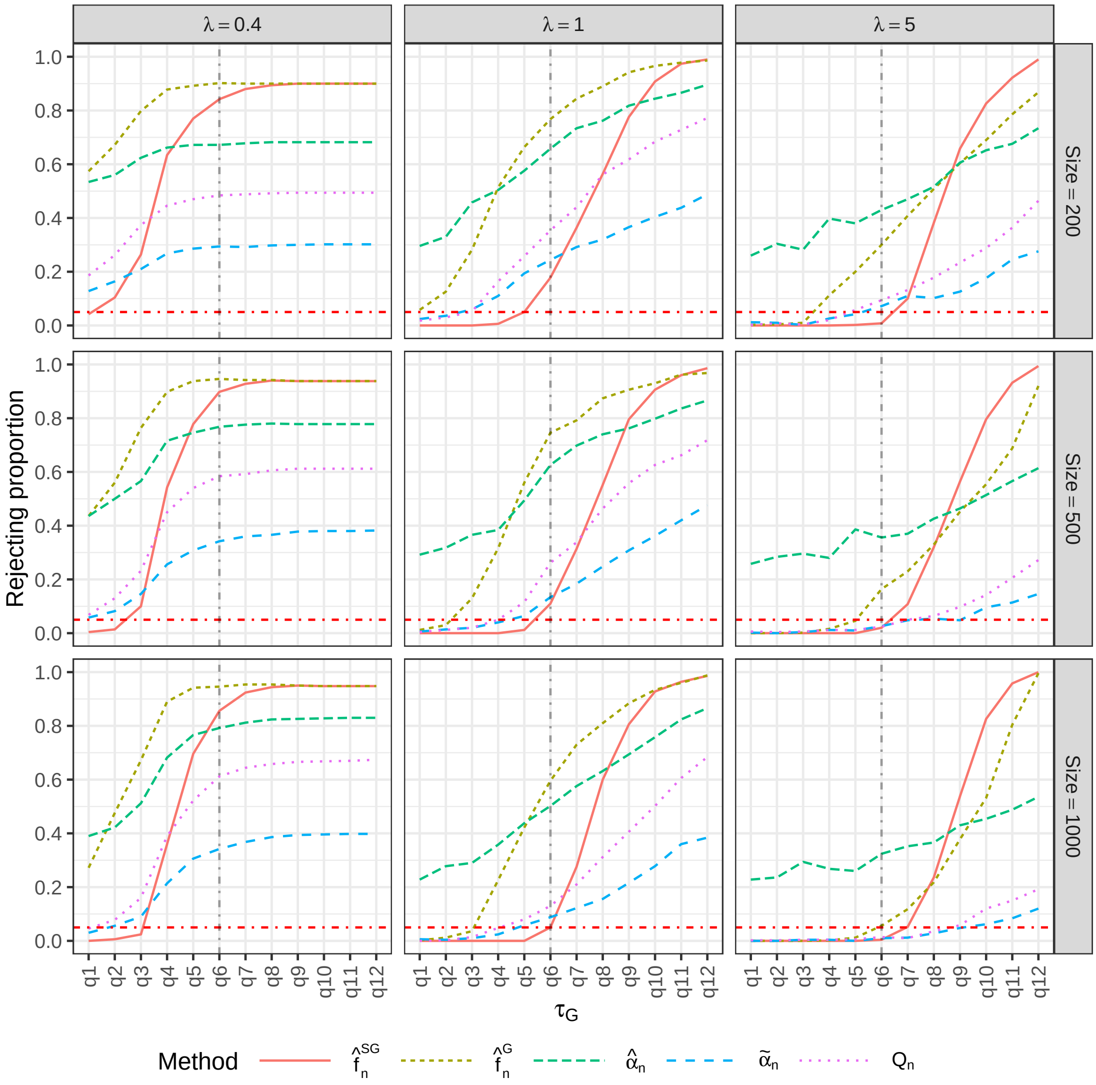

Figure S8: Rejection rate for Setting 2 when $p = 0.8$ (uncured fraction).

S1.2.3 Setting 3

Figures S9–S11 depict the rejection rate of insufficient follow-up against τ_G for Setting 3 when p is 0.2, 0.6, and 0.8. In this setting, the density f_u decreases faster and we observe a worse behaviour in terms of empirical level, particularly when p, n and $\Delta G(\tau_G)$ are smaller.

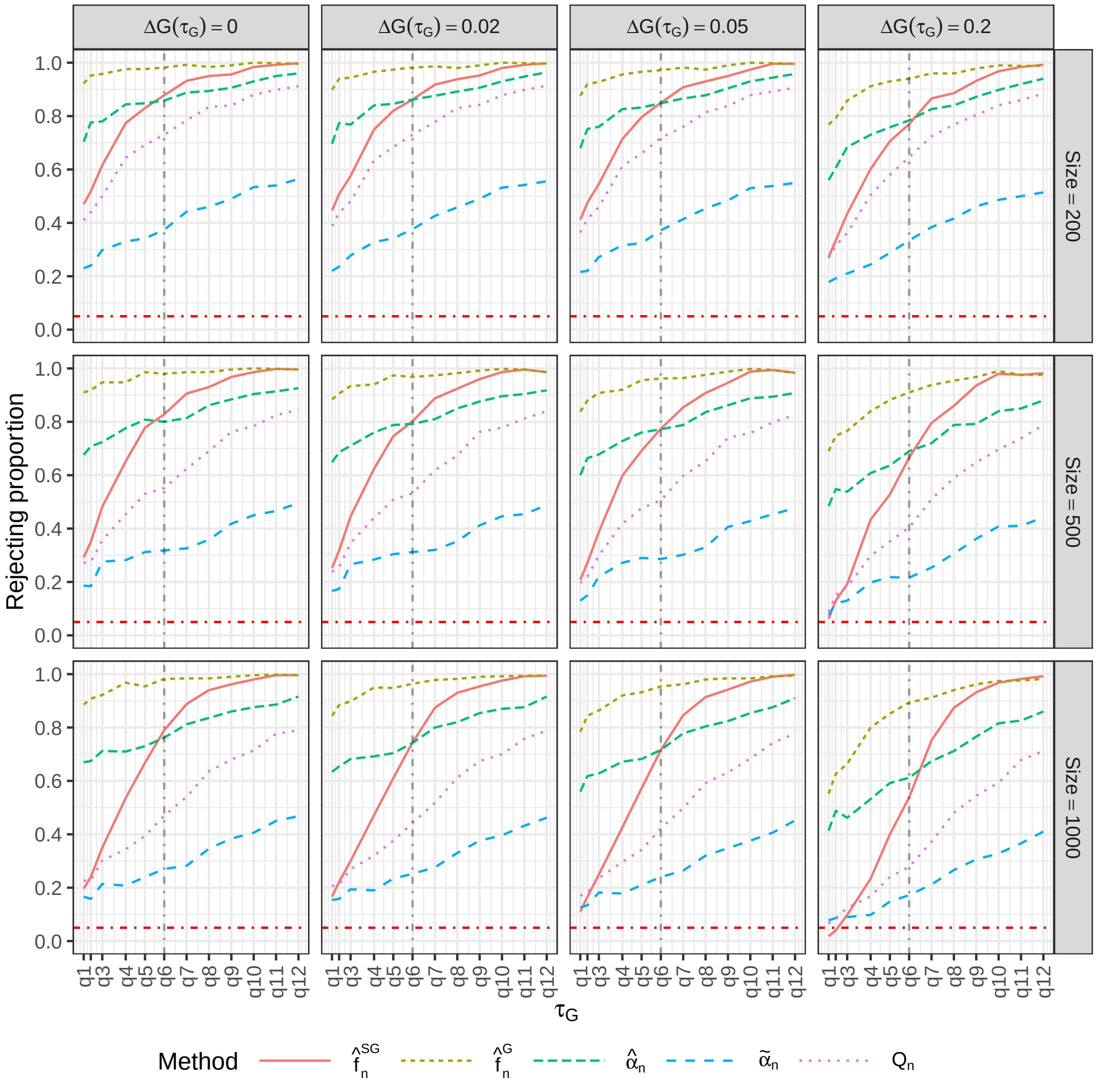

Figure S9: Rejection rate for Setting 3 when $p = 0.2$ (uncured fraction).

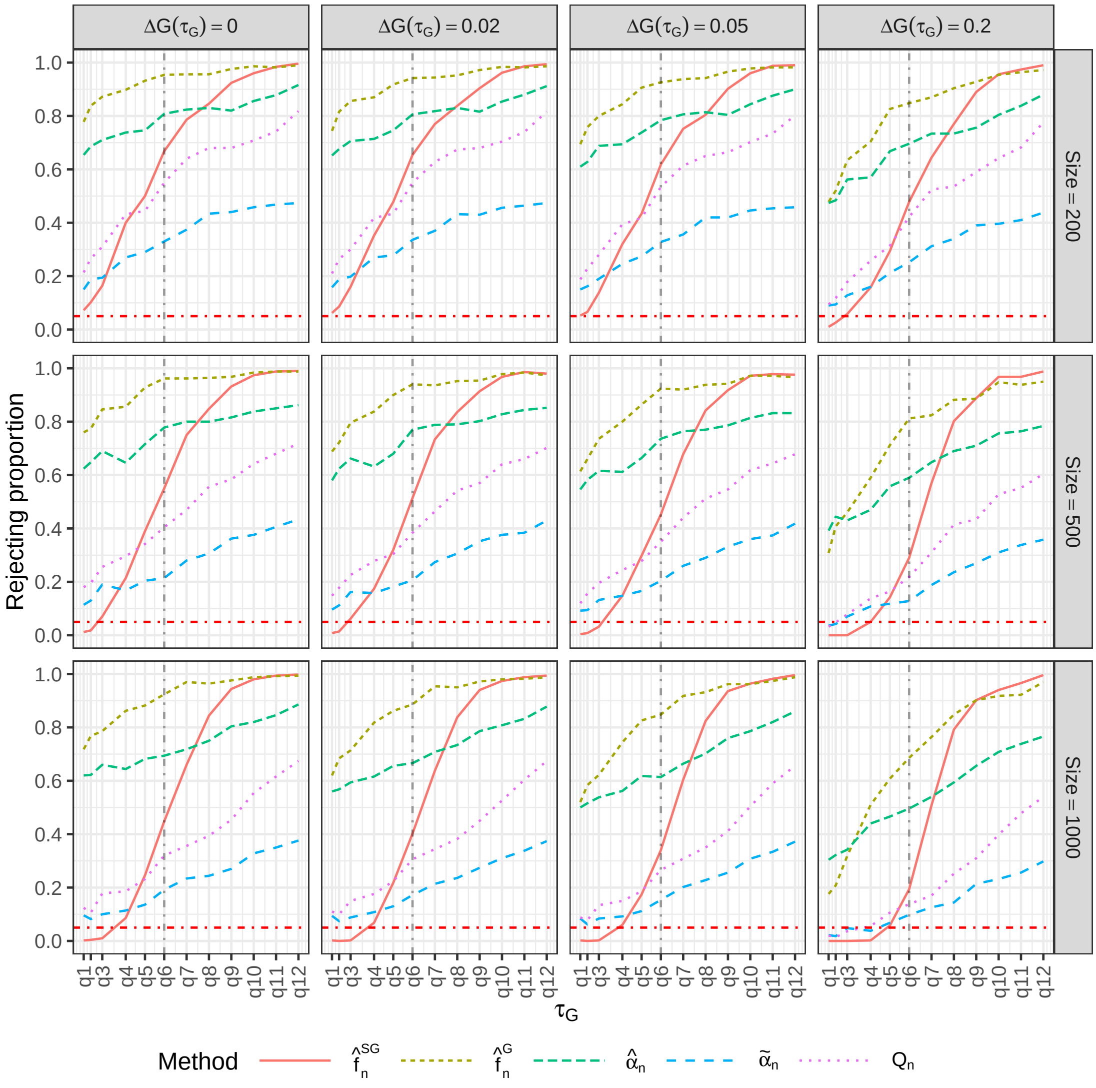

Figure S10: Rejection rate for Setting 3 when $p = 0.6$ (uncured fraction).

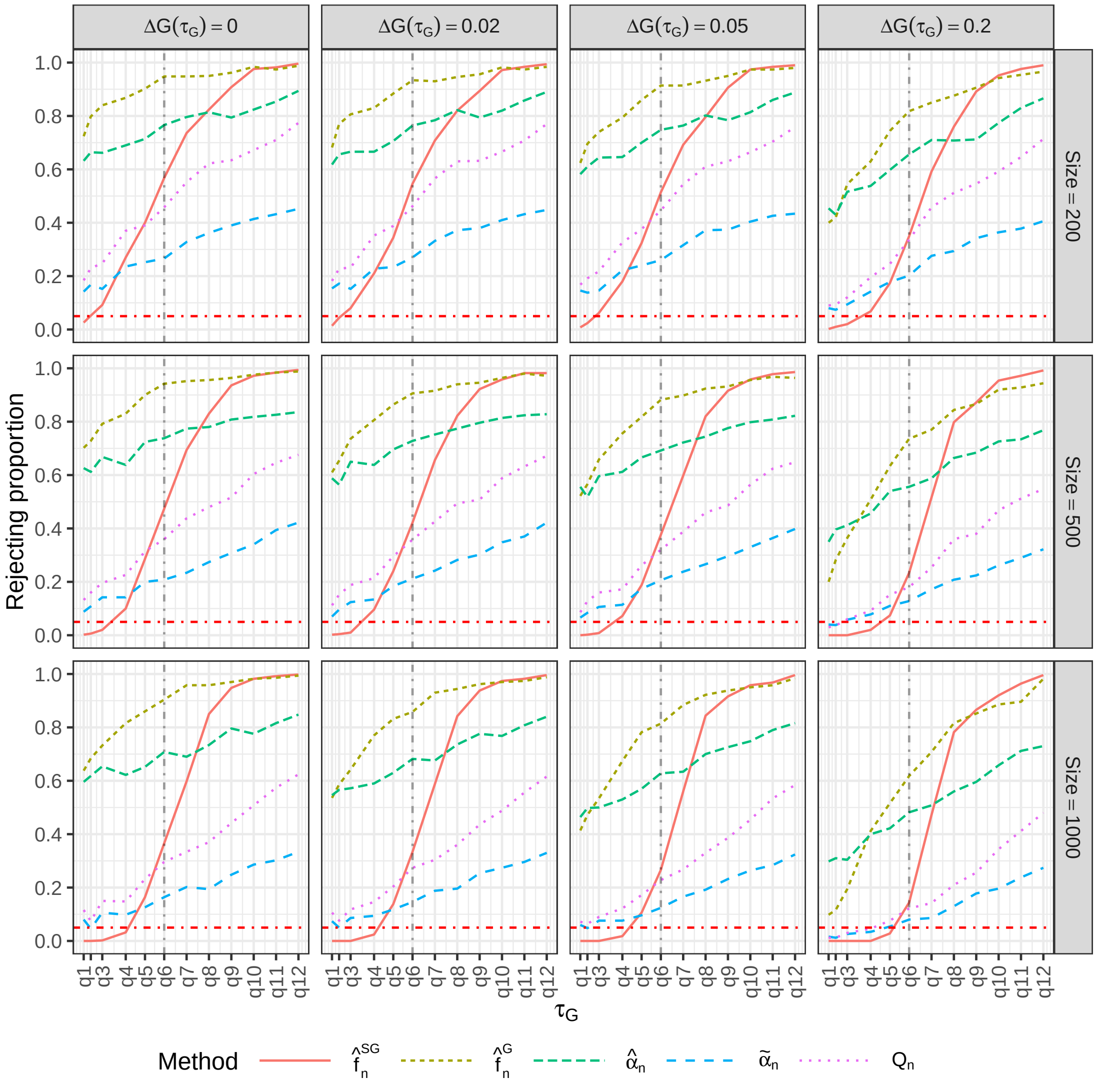

Figure S11: Rejection rate for Setting 3 when $p = 0.8$ (uncured fraction).

Changing ϵ from 0.01 to 0.005

Figure S12 shows the rejection rates of insufficient follow-up for $\hat{f}_{nh}^{SG}$, when $\epsilon = 0.005$ is used for $\tilde{H}_0 : \tau_G \leq q_{1-\epsilon}$, meaning that we consider the follow-up as insufficient when τ_G is less than or equal to the 99.5% quantile of F_u. Under such a stricter characterization of insufficient follow-up, the rejection proportion is lower compared to the results for $\epsilon = 0.01$. Note that the parameter τ is fixed to the 99.95% quantile of F_u for both cases.

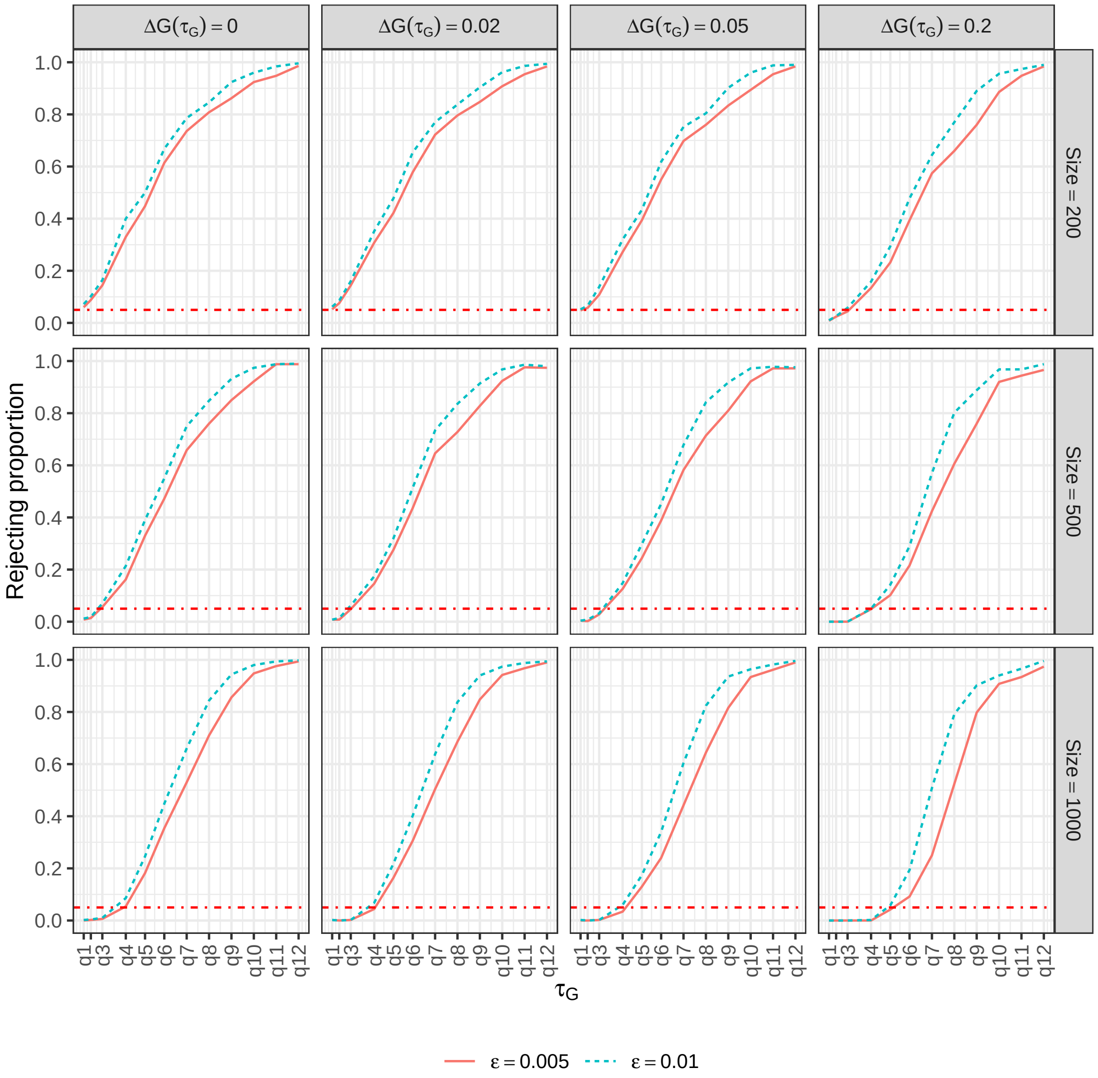

Figure S12: Rejection rate of insufficient follow-up for the test based on $\hat{f}_{nh}^{SG}$ in Setting 3 when $p = 0.6$ (uncured fraction). q_6 corresponds to $q_{0.99}$; and $q_{0.995}$ locates between q_7 and q_8.

S1.2.4 Setting 4

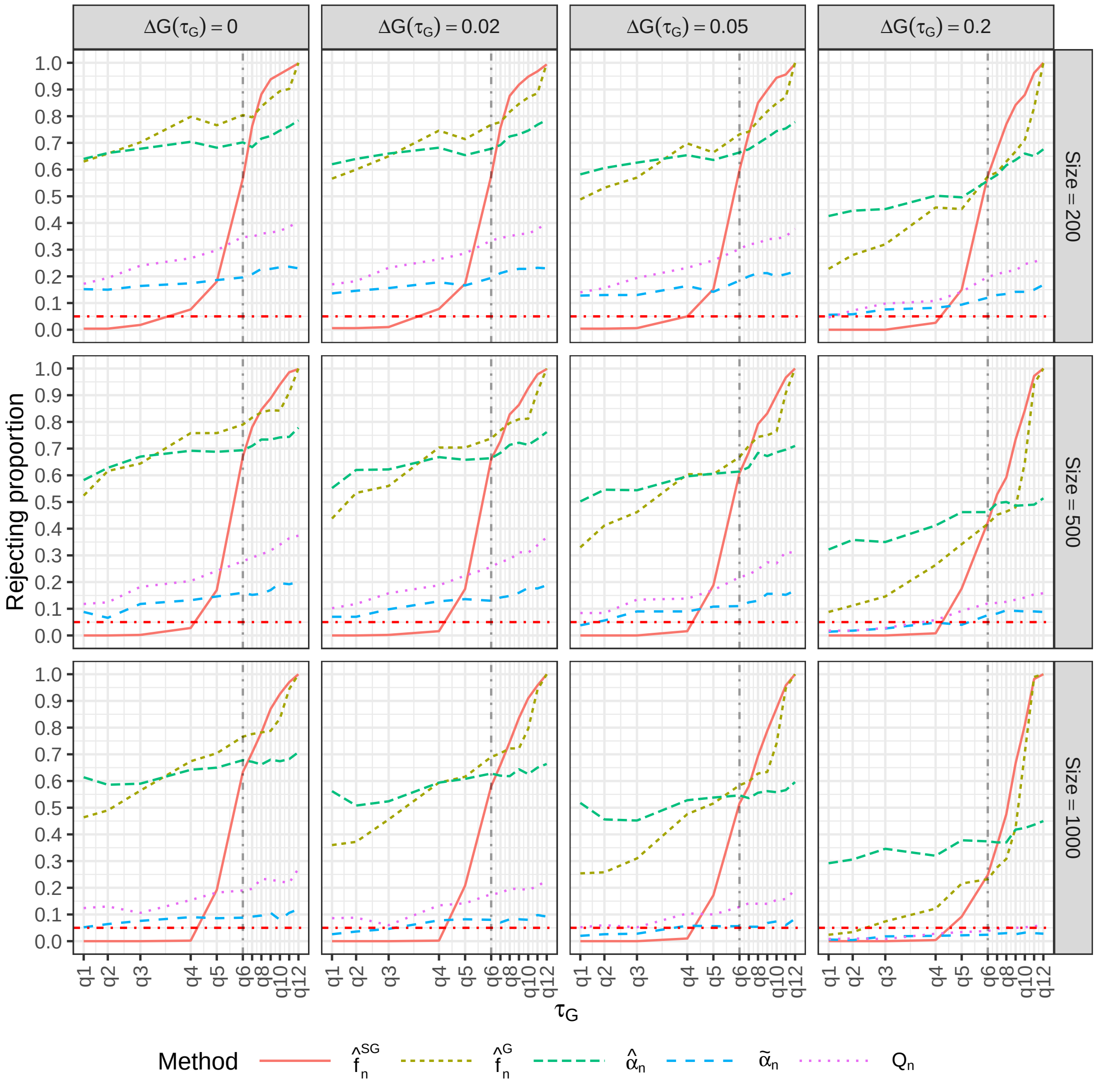

Figure S13: Rejection rate of insufficient follow-up for different methods in Setting 4 when $p = 0.6$ (uncured fraction).

S1.2.5 Setting 5

Figure S14 shows the rejection rate of insufficient follow-up against τ_G for Setting 5. The uncured subjects have a truncated exponential distribution with parameter 5 and the censoring time follows an exponential distribution with rate $\lambda_C \in \{0.5, 3\}$ and truncated at τ_G. The censoring becomes heavier when λ_C increases. $\hat{f}_{nh}^{SG}$ has a steeper rejection curve among the investigated methods, although the rejection rate of $\hat{f}_{nh}^{SG}$ is higher than the significance level when τ_G is between q_4 and q_6, and $\lambda_C = 3$. The Q_n test shows very little power to detect sufficient follow-up, particularly when the censoring rate is higher.

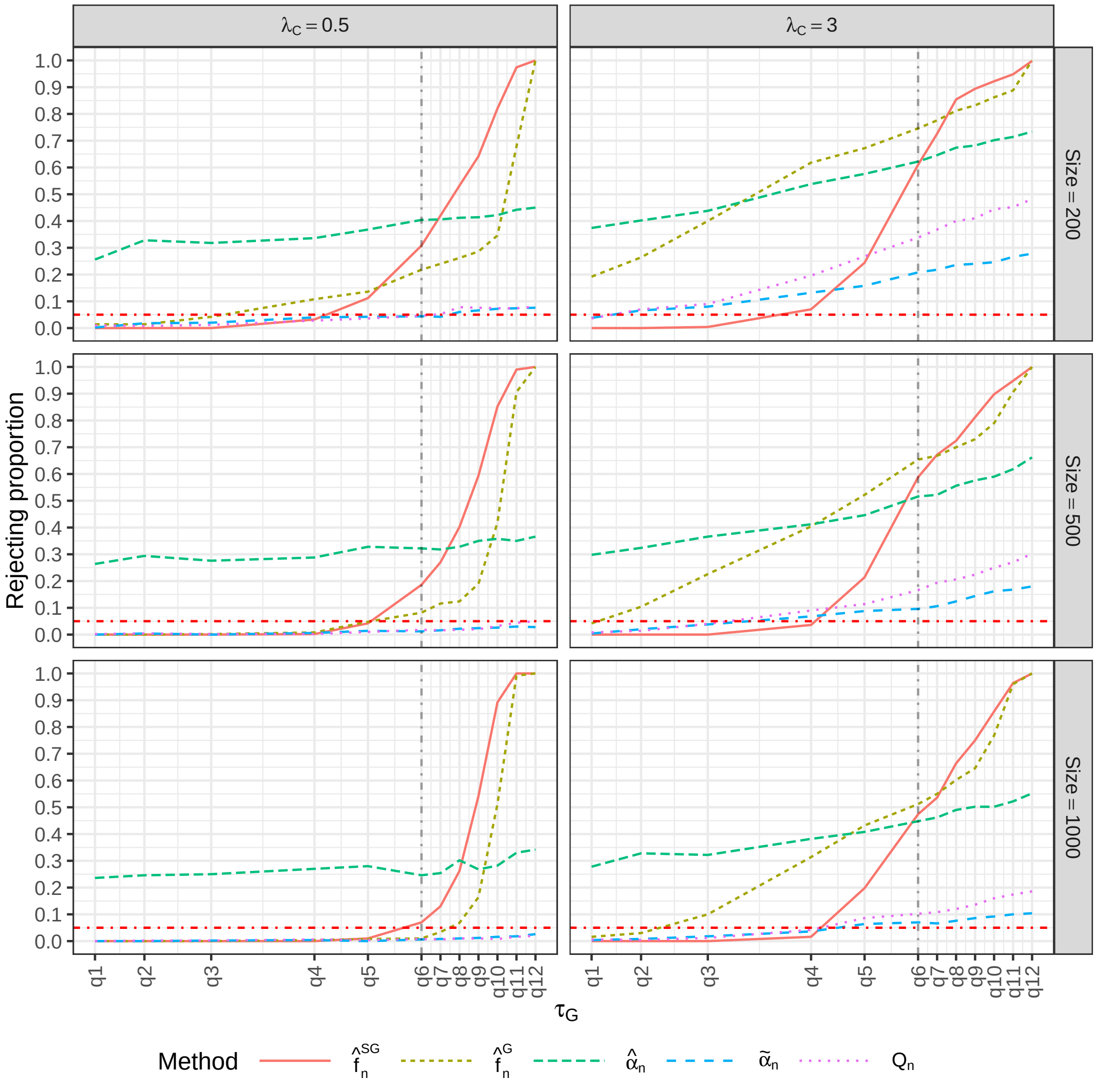

Figure S14: Rejection rate Setting 5 when $p = 0.6$ (uncured fraction).

S1.2.6 Setting 6

In Setting 6, the uncured subjects have an exponential distribution with rate of 1 and the censoring time follows an exponential distribution with rate of 0.5. The censoring rates, when $p = 0.2$, 0.6 and 0.8, are around 86%, 60% and 46%, respectively. In particular $\tau_{F_u} = \infty$ and $\tau_G = \infty$, and this setting is regarded as sufficient follow-up under $\check{H}_0 : \tau_{F_u} \le \tau_G$ but it is not sufficient follow-up under our formulation $\tilde{H}_0 : q_{1-\epsilon} \ge \tau_G$. Table S7 shows the rejection proportion of null hypotheses considered by the different tests. For the T_n-test, for which $\check{H}_0$ is true, the empirical level is around the significance level. For computation of our test statistics we use the maximum observed survival time $y_{(n)}$ instead of τ_G for each generated dataset, meaning that essentially, we consider the follow-up as insufficient when $q_{1-\epsilon} \ge y_{(n)}$. For the simulation with $p = 0.8$ and sample size of 200, there are 5 out of 500 replications that $q_{1-\epsilon} < y_{(n)}$. This is not observed for the remaining cases. The parameter τ is set to $y_{(n)} + \delta$, where $\delta \in \{1, 2\}$, which is considered reasonable given the range of values of $y_{(n)}$. The empirical power of the proposed methods using $\hat{f}_{nh}^{SG}$ and $\hat{f}_n^G$ are close to 1, among all different p's and sample sizes.

Table S7: Simulation result (in terms of rejection rate of the null hypotheses) for Setting 6. τ is set to $y_{(n)} + \delta$ for the tests based on $\hat{f}_n^G$ and $\hat{f}_{nh}^{SG}$.

			$\tilde{H}_0 : q_{1-\epsilon} \ge \tau_G$		$H_0 : \tau_{F_u} \ge \tau_G$			$\check{H}_0 : \tau_{F_u} \le \tau_G$
p	size	δ	$\hat{f}_n^G$	$\hat{f}_{nh}^{SG}$	α_n	$\tilde{\alpha}_n$	Q_n	T_n
0.2	200	1	1	1	0.998	0.814	0.996	0
		2	1	1				
	500	1	1	1	0.994	0.854	0.994	0.002
		2	1	1				
	1000	1	1	1	1	0.898	1	0.002
		2	1	1				
0.6	200	1	0.992	0.988	0.976	0.734	0.956	0
		2	0.992	0.984				
	500	1	1	1	0.992	0.79	0.98	0
		2	1	0.998				
	1000	1	1	1	0.994	0.876	0.99	0
		2	1	1				
0.8	200	1	0.974	0.942	0.948	0.624	0.876	0.008
		2	0.974	0.928				
	500	1	0.996	0.994	0.97	0.728	0.94	0.014
		2	0.996	0.986				
	1000	1	0.998	0.998	0.98	0.81	0.966	0.008
		2	0.998	0.998				

S1.2.7 Setting 7

In Setting 7, the uncured fraction p is 0.6, the censoring time follows the uniform distribution as in Setting 1 with $\tau_G = 6.5$ and $\Delta G(\tau_G-) = 0.02$. The uncured survival time follows a mixture of two log-normal distributions with the following distribution function:

$$F_u(t) = 0.7\Phi\left(\frac{\log(t) - \mu_1}{\sigma_1}\right) + 0.3\Phi\left(\frac{\log(t) - \mu_2}{\sigma_2}\right),$$

where $\mu_1 = 0$, $\sigma_1 = 1$, $\mu_2 = \log 8$, $\sigma_2 = 0.3$, and Φ is the cumulative distribution function of the standard normal distribution. The mean of the second log-normal distribution is $\exp(\mu_2 + \sigma_2^2/2) \approx 8.3682 > \tau_G$. The density f_u is non-monotone and therefore the non-increasing assumption of f_u for the proposed method is not satisfied. This setting is regarded as insufficient follow-up under $\tilde{H}_0 : q_{1-\epsilon} \geq \tau_G$ with $\epsilon = 0.01$ or $q_{1-\epsilon} \approx 14.44$. The censoring rate of this setting is around 67.06%. The simulation study is carried out using a sample size of 500 and 500 replications. The parameter τ for the proposed method is set to the 99.95% quantile of F_u, which is about 24.64. Table S8 shows the rejection rate of insufficient follow-up for different methods. For the proposed method using $\hat{f}_{nh}^{SG}$ and the Q_n test, the rejection rate is below the nominal level of 5%.

Table S8: Rejection rate of insufficient follow-up for different methods in Setting 7.

Method	Rejection rate
$\hat{f}_n^G$	0.390
$\hat{f}_{nh}^{SG}$	0.008
Q_n	0.038

S2 Technical lemmas and proofs

Recall that $\hat{F}_n$ is the Kaplan–Meier estimator of the distribution function F. Assume that F is continuous and G is right-continuous. Major and Rejtö (1988) showed that the process $\hat{F}_n(t) - F(t)$ can be approximated by a Gaussian process on the time interval $[0, t_0]$ with $t_0 < \tau_H$. Note that the right extreme of the observed event time τ_H can be expressed as $\tau_H = \tau_F \wedge \tau_G$. Note that $\tau_F = \infty$ in the presence of cured subjects. We therefore only consider the time up to τ_G. With an additional assumption on the censoring distribution that G has a jump at its right extreme τ_G, such result can be extended to the time interval $[0, \tau_G]$. This can be shown using the construction in Remark 3 of Major and Rejtö (1988).

Lemma 1. *Suppose the censoring distribution G has a jump at τ_G, i.e. $\Delta G(\tau_G) = 1 - G(\tau_G-) > 0$. The we have, for $x > 0$,*

$$\mathbb{P}\left[\sup_{t\leq\tau_G} n\left|\hat{F}_n(t) - F(t) - n^{-1/2}\left\{1 - F(t)\right\} W \circ L(t)\right| > K_1 \log n + x\right] < K_2 e^{-K_3 x},$$

where K_1, K_2 and K_3 are positive constants, W is a Brownian motion, and

$$L(t) = \int_0^t \frac{\mathrm{d}F(u)}{(1 - G(u-))\,(1 - F(u))^2}.$$

With Lemma 1, we apply the arguments in Lopuhaä and Musta (2017) to show the asymptotic normality of $\hat{f}_{nh}^{SG}(\tau_G)$ below. We denote the least concave majorant of the Kaplan–Meier estimator by $\hat{F}_n^G$.

Proof of Theorem 2. To show the asymptotic normality of $\hat{f}_{nh}^{SG}(\tau_G)$, we first decompose $\hat{f}_{nh}^{SG}(\tau_G) - f(\tau_G)$ into three parts:

$$\begin{aligned}
\hat{f}_{nh}^{SG}(\tau_G) - f(\tau_G) &= \int_{\tau_G - h}^{\tau_G} \frac{1}{h} k_{B,\tau_G}\left(\frac{\tau_G - u}{h}\right) \mathrm{d}F(u) - f(\tau_G) \\
&\quad + \int_{\tau_G - h}^{\tau_G} \frac{1}{h} k_{B,\tau_G}\left(\frac{\tau_G - u}{h}\right) \mathrm{d}(\hat{F}_n - F)(u) \\
&\quad + \int_{\tau_G - h}^{\tau_G} \frac{1}{h} k_{B,\tau_G}\left(\frac{\tau_G - u}{h}\right) \mathrm{d}(\hat{F}_n^G - \hat{F}_n)(u) \\
&= (I) + (II) + (III).
\end{aligned}$$

For (I), since f_u is twice continuously differentiable and by the properties of the boundary kernel, we have

$$\begin{aligned}
n^{2/5}(I) &= n^{2/5} \int_{\tau_G - h}^{\tau_G} \frac{1}{h} k_{B,\tau_G}\left(\frac{\tau_G - u}{h}\right) \{f(u) - f(\tau_G)\}\,\mathrm{d}u \\
&= n^{2/5} \int_0^1 k_{B,\tau_G}(v)\,\{f(\tau_G - hv) - f(\tau_G)\}\,\mathrm{d}v \\
&= n^{2/5} \int_0^1 k_{B,\tau_G}(v) \left\{-f'(\tau_G)hv + \frac{1}{2} f''(\xi_n) h^2 v^2\right\} \mathrm{d}v \\
&\to \frac{1}{2} c^2 f''(\tau_G) \int_0^1 v^2 k_{B,\tau_G}(v)\mathrm{d}v, \quad \text{as } n \to \infty,
\end{aligned}$$

where $0 < \tau_G - \xi_n < hv < h \to 0$ as $n \to \infty$.

For (III), with the strong approximation in Lemma 1, we can apply the argument in the proof of Lemma 4.3 in Lopuhaä and Musta (2017) to show that

$$\sup_{t \in [0, \tau_G]} \left| \hat{F}_n^G(t) - \hat{F}_n(t) \right| = O_P\left(\frac{\log n}{n}\right)^{2/3}. \tag{S1}$$

The idea is to verify the four conditions in Durot and Lopuhaä (2014) to establish such result. Therefore, $n^{2/5}(III)$ converges to zero in probability. Specifically, using the integration by parts and a change of variable, we have

$$\begin{aligned}
(III) &= \frac{1}{h} k_{B,\tau_G}\left(\frac{\tau_G - u}{h}\right) (\hat{F}_n^G - \hat{F}_n)(u) \Big|_{\tau_G - h}^{\tau_G} - \frac{1}{h} \int_0^1 (\hat{F}_n^G - \hat{F}_n)(\tau_G - hv) k'_{B,\tau_G}(v)\mathrm{d}v \\
&= O_P\left(\frac{1}{h}\left(\frac{\log n}{n}\right)^{2/3}\right),
\end{aligned}$$

where the last equality follows from the result in (S1) along with the boundedness of k and k', and consequently of k_{B,τ_G} and k'_{B,τ_G}. Therefore, $n^{2/5}(III) = o_P(1)$ follows from the assumption that $hn^{1/5} \to c \in (0, \infty)$ as $n \to \infty$.
For (II), we have

$$n^{2/5}(II) = \frac{1}{\sqrt{hn^{1/5}}} \int_0^1 k_{B,\tau_G}(v) \mathrm{d}\hat{W}_n(v),$$

where, for $v \in [0, 1]$,

$$\begin{aligned}
\hat{W}_n(v) = &\sqrt{\frac{n}{h}} \left\{ \hat{F}_n(\tau_G - hv) - F(\tau_G - hv) - n^{-1/2}(1 - F(\tau_G - hv))W \circ L(\tau_G - hv) \right\} \\
&- \sqrt{\frac{n}{h}} \left\{ \hat{F}_n(\tau_G) - F(\tau_G) - n^{-1/2}(1 - F(\tau_G))W \circ L(\tau_G) \right\} \\
&+ \sqrt{\frac{1}{h}} (1 - F(\tau_G)) \left\{ W \circ L(\tau_G - hv) - W \circ L(\tau_G) \right\} \\
&+ \sqrt{\frac{1}{h}} (F(\tau_G) - F(\tau_G - hv)) \left\{ W \circ L(\tau_G - hv) \right\}.
\end{aligned} \tag{S2}$$

Using the strong approximation in Lemma 1, together with the arguments from the proof of Theorem 4.4 in Lopuhaä and Musta (2017), it can be shown that the first two terms on the right-hand side of (S2) converges to 0 in probability, uniformly in v. The last term also converges to 0 in probability, uniformly in v, by applying the argument from the same proof in Lopuhaä and Musta (2017), which relies on the maximal inequality for Brownian motion. Therefore, $n^{2/5}(II)$ is dominated by the term

$$\frac{1}{\sqrt{hn^{1/5}}} \int_0^1 k_{B,\tau_G}(v) \mathrm{d}W_n(v),$$

where $W_n(v) = h^{-1/2} (1 - F(\tau_G)) \{W \circ L(\tau_G - hv) - W \circ L(\tau_G)\}$, for $v \in [0, 1]$. By scaling, time reversal, and symmetry of a Brownian motion, we have

$$\frac{1}{\sqrt{h}} \{W \circ L(\tau_G - hv) - W \circ L(\tau_G)\} \stackrel{d}{=} \tilde{W} \left(\frac{L(\tau_G) - L(\tau_G - hv)}{h} \right), \tag{S3}$$

where $\tilde{W}$ is a one-sided Brownian motion. By the uniform continuity of the one-sided Brownian motion on the compact interval $[0, 1]$, we have

$$\sup_{v \in [0,1]} \left| \tilde{W} \left(\frac{L(\tau_G) - L(\tau_G - hv)}{h} \right) - \tilde{W} \left(L'(\tau_G)v \right) \right| \xrightarrow{\mathbb{P}} 0, \tag{S4}$$

where $L'(\tau_G) = \frac{f(\tau_G)}{(1-G(\tau_G-))(1-F(\tau_G))^2}$. Using integration by parts, we have

$$\frac{1}{\sqrt{hn^{1/5}}} \int_0^1 k_{B,\tau_G}(v) \mathrm{d}W_n(v) = \frac{1}{\sqrt{hn^{1/5}}} \left\{ k_{B,\tau_G}(v) W_n(v) \Big|_0^1 - \int_0^1 W_n(v) \mathrm{d}k_{B,\tau_G}(v) \right\}.$$

Using (S3) followed by (S4), it can be shown that the term on the right-hand side in the above display converges in distribution to

$$\begin{aligned}&\frac{1-F(\tau_G)}{\sqrt{c}}\left\{k_{B,\tau_G}(v)\tilde{W}\left(L'(\tau_G)v\right)\Big|_0^1-\int_0^1\tilde{W}\left(L'(\tau_G)v\right)\mathrm{d}k_{B,\tau_G}(v)\right\}\\&=(1-F(\tau_G))\sqrt{L'(\tau_G)/c}\int_0^1 k_{B,\tau_G}(v)\mathrm{d}\tilde{W}(v),\end{aligned}$$

where we used the rescaling of a Brownian motion and integration by parts. Therefore we have

$$\begin{aligned}\frac{1}{\sqrt{hn^{1/5}}}\int_0^1 k_{B,\tau_G}(v)\mathrm{d}W_n(v)\xrightarrow{d}(1-F(\tau_G))\sqrt{L'(\tau_G)/c}\int_0^1 k_{B,\tau_G}(v)\mathrm{d}\tilde{W}(v)\\ \sim N\left(0,\frac{f(\tau_G)}{c[1-G(\tau_G-)]}\int_0^1 k_{B,\tau_G}(v)^2\mathrm{d}v\right),\end{aligned}$$

where we use that the integral with respect to the Brownian motion is a Normal distributed variable (see for example Durrett (2018)). □

Proof of Proposition 1. Let $\tau_G\le q_{1-\epsilon}$. We have

$$\begin{aligned}&\mathbb{P}\left[\hat{f}_n^G(\tau_G-cn^{-a})\le\frac{\epsilon\hat{F}_n(\tau_G)}{\tau-\tau_G}-A_1^{-1}n^{-(1-a)/2}Q_{1-\alpha}^G\right]\\&=\mathbb{P}\left[f(\tau_G)-\hat{f}_n^G(\tau_G-cn^{-a})\ge f(\tau_G)-\frac{\epsilon\{\hat{F}_n(\tau_G)-F(\tau_G)\}}{\tau-\tau_G}-\frac{\epsilon F(\tau_G)}{\tau-\tau_G}\right.\\&\qquad\left.+A_1^{-1}n^{-(1-a)/2}Q_{1-\alpha}^G\right]\\&\le\mathbb{P}\left[A_1n^{(1-a)/2}\{f(\tau_G)-\hat{f}_n^G(\tau_G-cn^{-a})\}\right.\\&\qquad\left.\ge Q_{1-\alpha}^G+\frac{A_1n^{(1-a)/2}p\eta}{\tau-\tau_G}-\frac{\epsilon A_1n^{(1-a)/2}\{\hat{F}_n(\tau_G)-F(\tau_G)\}}{\tau-\tau_G}\right].\end{aligned}$$

Here the last inequality follows from the fact that $\tilde{H}_0: q_{1-\epsilon}\ge\tau_G$ entails $f(\tau_G)\ge\frac{p(\epsilon-\eta)}{\tau-\tau_G}$, and $F(\tau_G)\le p$. By Theorem 1 and Lemma 1, the probability on the right hand side converges to α, which concludes the proof. Note that the upper bound on the rejection probability is still asymptotically bounded by α even if A_1 is replaced by a consistent estimator $\hat{A}_1$ since one would only have some additional terms involving $\hat{A}_1-A_1$, which would converge to zero. □

Proof of Proposition 2. Let $\tau_G \leq q_{1-\epsilon}$. We have

$$\begin{aligned}
&\mathbb{P}\left(\hat{f}_{nh}^{SG}(\tau_G) \leq \frac{\epsilon \hat{F}_n(\tau_G)}{\tau - \tau_G} + n^{-2/5} Q_\alpha^{SG}\right) \\
&= \mathbb{P}\left(\hat{f}_{nh}^{SG}(\tau_G) - f(\tau_G) \leq \frac{\epsilon\{\hat{F}_n(\tau_G) - F(\tau_G)\}}{\tau - \tau_G} + \frac{\epsilon F(\tau_G)}{\tau - \tau_G} - f(\tau_G) + n^{-2/5} Q_\alpha^{SG}\right) \\
&\leq \mathbb{P}\left(\hat{f}_{nh}^{SG}(\tau_G) - f(\tau_G) \leq \frac{\epsilon\{\hat{F}_n(\tau_G) - F(\tau_G)\}}{\tau - \tau_G} + \frac{p\eta}{\tau - \tau_G} + n^{-2/5} Q_\alpha^{SG}\right) \\
&= \mathbb{P}\left(n^{2/5}\{\hat{f}_{nh}^{SG}(\tau_G) - f(\tau_G)\} \leq \frac{\epsilon n^{2/5}\{\hat{F}_n(\tau_G) - F(\tau_G)\}}{\tau - \tau_G} + \frac{n^{2/5} p\eta}{\tau - \tau_G} + Q_\alpha^{SG}\right).
\end{aligned}$$

Here the inequality follows from the fact that $\tilde{H}_0 : q_{1-\epsilon} \geq \tau_G$ entails $f(\tau_G) \geq \frac{p(\epsilon-\eta)}{\tau-\tau_G}$, and $F(\tau_G) \leq p$. By Theorem 2 and Lemma 1, the probability on the right hand side converges to α, which concludes the proof. Note that the upper bound on the rejection probability is still asymptotically bounded by α even if Q_α^{SG} is replaced by a consistent estimator $\hat{Q}_\alpha^{SG}$ since one would only have an additional term $\hat{Q}_\alpha^{SG} - Q_\alpha^{SG}$, which would converge to zero. □

S3 Algorithm

Algorithm S1 Bootstrapping procedure

Require:

Orignal sample data $\{(y_i, \delta_i) : i = 1, \cdots, n\}$

Level of the test α

Number of bootstrap iterations B

Obtain a smooth KME of F by

$$\tilde{F}_{nh_0}(t) = \int_{(t-h_0)\vee 0}^{(t+h_0)\wedge\tau_G} \frac{1}{h_0} k^{(t)}\left(\frac{t-v}{h_0}\right) \hat{F}_n^G(v)\mathrm{d}v, \quad t \in [0, \tau_G],$$

where $\hat{F}_n^G$ is the least concave majorant (LCM) of the KME $\hat{F}_n$.

Compute the derivative $\tilde{f}_{nh_0}(\tau_G)$ of $\tilde{F}_{nh_0}$ at τ_G.

Estimate G by the reversed KME, denoted by $\hat{G}_n$, using $\{(y_i, \delta_i) : i = 1, \cdots, n\}$.

for $b = 1, \cdots, B$ **do**

Draw $(c^*_{b,1}, \cdots, c^*_{b,n})$ from $\hat{G}_n$;

Draw $(t^*_{b,1}, \cdots, t^*_{b,n})$ from $\tilde{F}_{nh_0}$;

▷ Set $t^*_{b,i} = \infty$ if $u_{b,i} > \sup_t \tilde{F}_{nh_0}(t)$, where $u_{b,i}$ is the generated standard uniform random variate.

Construct $\{(y^*_{b,i}, \delta^*_{b,i}), i = 1, \cdots, n\}$, where $y^*_{b,i} = t^*_{b,i} \wedge c^*_{b,i}$ and $\delta^*_{b,i} = \mathbb{1}_{\{t^*_{b,i} \leq c^*_{b,i}\}}$.

Obtain the bootstrap estimate $\hat{f}^{SG*}_{nh,b}(\tau_G)$ using $\{(y^*_{b,i}, \delta^*_{b,i}) : i = 1, \cdots, n\}$.

end for

Approximate the critical value of the test by the α-quantile of $\{\hat{f}^{SG*}_{nh,b}(\tau_G) - \tilde{f}_{nh_0}(\tau_G) : b = 1, \cdots, B\}$

S4 Assumptions of Q_n and T_n test

The assumptions needed for the Q_n test to obtain the asymptotic distribution of $n\hat{q}_n$ when $\tau_G < \tau_{F_u}$ are:

(a) $1 - G(\tau_G - x) = a_G(1 + o(1))x^\gamma$, as $x \downarrow 0$, where a_G and γ are positive constants;

(b) F_u has a density f_u in a neighborhood of τ_G, which are positive and continuous at τ_G.

The assumptions needed for the T_n test are:

(a) F_u is continuous at τ_{F_u} if $\tau_{F_u} < \infty$;

(b) $\int_0^{\tau_{F_u}} \frac{\mathrm{d}F_u(s)}{1-G(s-)} < \infty$;

(c) $\lim_{n\to\infty} n(1 - G(\tau_G - x/\sqrt{n})) = \infty$ for all $x > 0$;

(d) F_u has a finite second derivative F_u'' in $(\tau_{F_u} - \epsilon, \tau_{F_u})$ for some $\epsilon > 0$ and satisfies

$$\lim_{t\to\tau_{F_u}} \frac{F_u''(t)(1 - F_u(t))}{(F_u'(t))^2} = -1.$$

Bibliography

Durot, C. and Lopuhaä, H. P. (2014). A Kiefer-Wolfowitz type of result in a general setting, with an application to smooth monotone estimation. *Electronic Journal of Statistics*, 8(2):2479–2513.

Durrett, R. (2018). *Stochastic calculus: a practical introduction.* CRC press.

Lopuhaä, H. P. and Musta, E. (2017). Smooth estimation of a monotone hazard and a monotone density under random censoring. *Statistica Neerlandica*, 71(1):58–82.

Major, P. and Rejtö, L. (1988). Strong embedding of the estimator of the distribution function under random censorship. *The Annals of Statistics*, 16(3):1113–1132.

Maller, R. and Zhou, S. (1992). Estimating the proportion of immunes in a censored sample. *Biometrika*, 79(4):731–739.

Maller, R. and Zhou, S. (1994). Testing for sufficient follow-up and outliers in survival data. *Journal of the American Statistical Association*, 89(428):1499–1506.

Maller, R. and Zhou, X. (1996). *Survival Analysis with Long-Term Survivors.* Wiley series in probability and statistics: Applied probability and statistics. John Wiley & Sons, NY.

Selukar, S. and Othus, M. (2023). RECeUS: Ratio estimation of censored uncured subjects, a different approach for assessing cure model appropriateness in studies with long-term survivors. *Statistics in Medicine*, 42(3):209–227.
